# Combining Exercise Training and Testosterone Therapy in Older Women After Hip Fracture: The STEP-HI Randomized Clinical Trial

**DOI:** 10.1001/jamanetworkopen.2025.10512

**Published:** 2025-05-15

**Authors:** Ellen F. Binder, Jenna M. Bartley, Sarah D. Berry, Peter M. Doré, Steven R. Fisher, Richard H. Fortinsky, Camelia Guild, Douglas P. Kiel, George A. Kuchel, Robin L. Marcus, Christine M. McDonough, Kelly M. Monroe, Denise L. Orwig, Rocco A. Paluch, Dominic Reeds, Jennifer Stevens-Lapsley, Elena Volpi, Kenneth B. Schechtman, Jay S. Magaziner

**Affiliations:** 1 Division of General Medicine and Geriatrics, Washington University School of Medicine in St Louis, St Louis, Missouri; 2 UConn Center on Aging, University of Connecticut, Farmington; 3 Hinda and Arthur Marcus Institute for Aging Research, Hebrew SeniorLife, Boston, Massachusetts; 4 Department of Medicine, Beth Israel Deaconess Medical Center and Harvard Medical School, Boston, Massachusetts; 5 Division of Biostatistics, Washington University School of Medicine in St Louis, St Louis, Missouri; 6 Department of Physical Therapy, University of Texas Medical Branch, Galveston; 7 Department of Physical Therapy and Athletic Training, University of Utah, Salt Lake City; 8 School of Health and Rehabilitation Sciences, University of Pittsburgh, Pittsburgh, Pennsylvania; 9 Department of Epidemiology and Public Health, University of Maryland School of Medicine, Baltimore; 10 Jacobs School of Medicine, University at Buffalo, Buffalo, New York; 11 Biomedical Sciences, University at Buffalo, Buffalo, New York; 12 Division of Nutritional Science and Obesity Medicine, Washington University School of Medicine in St Louis, St Louis, Missouri; 13 Department of Physical Medicine and Rehabilitation, University of Colorado Anschutz Medical Campus, Denver; 14 Sam and Ann Barshop Institute for Longevity and Aging Studies, Division of Geriatrics, Gerontology and Palliative Medicine, University of Texas Health San Antonio, San Antonio; 15 San Antonio Geriatric Research, Education, and Clinical Center (GRECC), San Antonio, Texas; 16 Sealy Center on Aging, University of Texas Medical Branch, Galveston

## Abstract

**Importance:**

Despite receiving postacute rehabilitation services, many older women with a recent hip fracture repair are unable to return to their prefracture level of function. Whether testosterone therapy can enhance recovery after hip fracture in older women with persistent mobility impairments has not been fully examined.

**Objective:**

To evaluate the effects of a supervised exercise program combined with topical testosterone therapy on functional outcomes in older women with a recent hip fracture.

**Design, Setting, and Participants:**

This phase 3 double-blind, placebo-controlled randomized clinical trial, Starting a Testosterone and Exercise Program After Hip Injury (STEP-HI), was conducted at 8 US sites between December 2018 and August 2023. Participants were women aged 65 years or older with a recent surgical repair of a nonpathologic femur fracture, met objective criteria for mobility impairment, and were community dwelling after discharge from rehabilitation. During the first 14 months of the trial, participants were randomly assigned to 1 of 3 treatment groups: exercise plus topical testosterone gel, exercise plus placebo gel, or enhanced usual care. Statistical analysis, using a modified intention-to-treat approach, was performed from November 2023 to November 2024.

**Interventions:**

For 24 weeks, the 2 exercise groups underwent a supervised, multimodal high-intensity exercise program that included progressive resistance training with trained and certified exercise interventionists. Testosterone was provided in the form of a topical gel (generic 1.0% testosterone), and the placebo gel was chemically identical but without testosterone. The enhanced usual care group was prescribed a self-administered low-intensity home-based exercise program and a staff-led monthly health education session.

**Main Outcomes and Measures:**

The primary outcome was the between-group difference in the change in the 6-minute walking distance (6MWD) from baseline to 24 weeks.

**Results:**

Among the 129 women randomized (54 to the exercise plus testosterone group, 55 to the exercise plus placebo group, and 20 to the enhanced usual care group; mean [SD] age, 79.3 [8.4] years) included in the STEP-HI trial (122 (94.6%) provided primary outcome data for baseline and at least 1 time point (12 or 24 weeks). The mean (SD) change in the 6MWD between baseline and 24 weeks was not significantly different for exercise plus topical testosterone gel (n = 53; 42.7 [8.2] m) compared with exercise plus placebo gel (n = 51; 40.5 [8.4] m) and enhanced usual care (n = 18; 37.7 [14.8] m).

**Conclusions and Relevance:**

In the STEP-HI randomized clinical trial, supervised exercise training combined with testosterone therapy conducted in older women recovering from a hip fracture did not lead to significant improvement in 6MWD compared with supervised exercise alone. Adding testosterone therapy to exercise may not provide further benefits for long-distance mobility. Whether it can improve physical performance and mobility for short distances in this patient population warrants further study.

**Trial Registration:**

ClinicalTrials.gov Identifier: NCT02938923

## Key Points

**Question:**

Does exercise training combined with topical testosterone gel therapy improve mobility more than exercise training alone in older women after hip fracture?

**Findings:**

In this randomized clinical trial of 129 women with a recent hip fracture repair, 24 weeks of exercise training plus topical testosterone gel did not significantly increase 6-minute walking distance compared with exercise training plus placebo gel.

**Meaning:**

This finding suggests that topical testosterone therapy may not benefit long-distance mobility in this patient population.

## Teaser

This randomized clinical trial compares the benefits of adding topical testosterone gel to exercise vs exercise alone in older women recovering from hip fracture repair and experiencing mobility impairments.

## Introduction

Hip fractures represent a major public health problem. Annually in the US, an estimated 300 000 people aged 65 years or older sustain a hip fracture.^1^ As the number of older adults continues to increase, the burden of care for patients with hip fracture is expected to grow.^2^ Up to 76% of patients experience functional loss^3,4,5^ despite receiving rehabilitation as part of their fracture management.

Several studies have demonstrated the benefits of structured exercise programs for functional outcomes in older patients with hip fracture, although study outcomes and the magnitude of changes have varied widely.^6,7,8^ The addition of an anabolic agent has the potential to augment the effects of exercise on muscle function and thereby improve postfracture function.^9^ To date, most studies of testosterone therapy conducted in women have focused on ameliorating osteoporosis or low libido in menopausal women^10,11,12,13^ or anabolic muscle effects in younger, healthier women.^14,15,16,17^ A 2014 systematic review evaluating the use of anabolic steroids after hip fracture found that the quality of the studies was insufficient to draw conclusions on the effects and recommended further high-quality trials in the field.^18^ The aim of the Starting a Testosterone and Exercise Program After Hip Injury (STEP-HI) trial,^19^ was to evaluate the effects of a supervised exercise program combined with topical testosterone therapy on functional outcomes in older women with a recent hip fracture.

## Methods

### Study Design and Objectives

STEP-HI was a phase 3, multicenter, 3-group, double-blind, placebo-controlled randomized clinical trial conducted at 8 sites in the US between December 2018 and August 2023. The design of this trial was informed by previous unpublished pilot studies of testosterone therapy conducted in patients with hip fracture (ClinicalTrials.gov Identifiers: NCT00280267 and NCT00345969). The trial protocol is provided in Supplement 1. Participants provided written informed consent. Study procedures were approved by the Washington University in St Louis Institutional Review Board. We followed the Consolidated Standards of Reporting Trials (CONSORT) reporting guideline.

The study comprised 24 weeks of treatments and safety assessments. Over this time period, we compared 3 different intervention strategies in older women after a hip fracture: (1) supervised exercise plus topical testosterone gel, (2) supervised exercise plus placebo gel, and (3) enhanced usual care. Our primary hypothesis was that supervised exercise combined with topical testosterone therapy would result in greater gains in mobility as measured by the 6-Minute Walking Distance (6MWD) test compared with supervised exercise plus placebo.

### Participant Recruitment, Eligibility, and Baseline Assessments

Participants were recruited from sites of care for patients with hip fracture during the postsurgery recovery period, including skilled nursing facilities, home care programs, orthopedic practices, and outpatient physical therapy practices. Recruitment and screening strategies have been described elsewhere.^19^ An in-person screening visit was performed to confirm eligibility.

Inclusion criteria were female 65 years or older, surgical repair of a nonpathological fracture of the proximal femur, screening within 22 weeks of surgical repair date, community dwelling or in assisted living facility prior to the fracture, Modified Physical Performance Test score between 12 and 28 (indicating mild-to-moderate frailty),^20^ and total serum testosterone level of 60 ng/dL or less (to convert to nanomoles per liter, multiply by 0.0347). Exclusion criteria included patients with a Short Blessed Test^21^ score higher than 10 (indicating cognitive impairment) or inability to provide informed consent, factors affecting safety (eg, unstable medical conditions; history of breast, ovarian, or uterine cancer diagnosed within the previous 10 years), and potential confounders (eg, use of progestin- or androgen-containing compound within the previous 6 months; systemic corticosteroids [prednisone dose >5 mg daily for ≥90 days] in the previous 12 months). Women taking medications for osteoporosis were allowed to continue taking them. To enhance recruitment, adherence, and retention, we provided transportation to all study assessments and exercise sessions.

Reporting of race and ethnicity was mandated by the US National Institutes of Health, consistent with the policy of inclusion of women, minorities, and children as research participants. Study participants self-reported their race and ethnicity, which were categorized as American Indian or Alaska Native, Asian, Black or African American, Hispanic or Latino, Native Hawaiian or Other Pacific Islander, White, or other (1 participant specified Mexican) based on the National Institutes of Health policy on reporting race and ethnicity data.

Due to the onset of the COVID-19 pandemic, study recruitment was paused between April and July 2020. Study procedures implemented during that period have been described elsewhere.^19^

Baseline assessments included (1) primary and secondary outcomes and safety measures; (2) a standardized physical therapy evaluation to identify physical conditions that might limit the participant’s ability to perform the exercises and to guide the site exercise interventionist in implementing the protocol; (3) a standardized nutritional evaluation to calculate caloric and protein needs and to provide recommendations for optimizing their diet, tailored to their preferences and budget; and (4) a mammogram to rule out any breast neoplasms.

### Interventions

#### Supervised Exercise Program

Women in the exercise plus placebo gel and exercise plus topical testosterone gel groups performed a supervised, multimodal high-intensity exercise program that included progressive resistance training. It was conducted on 2 nonconsecutive days per week for 24 weeks at a dedicated exercise facility and was directly supervised by a certified exercise interventionist. The exercise program was provided in 2 successive phases.^19^ Phase 1 consisted of functional movements, flexibility, balance, and strengthening exercises. Phase 2 consisted primarily of high-intensity strength-training exercises. Participants in the 2 exercise groups were also expected to perform home exercises 3 other days per week that consisted of a progressive walking program and 3 exercises in the supervised program that varied over the intervention period.

Exercise interventionists were required to complete training and certification to administer the protocol. Their fidelity to the protocol was monitored using checklists completed during each exercise session, periodic direct observation of exercise sessions by a supervising physical therapist, and videos of sessions that were reviewed by the lead physical therapist (J.S.-L.) of the clinical coordinating center (CCC). Adherence to the exercise protocol was defined as the percentage of sessions completed out of a possible 48 sessions, whereby completion was defined as 70% or more of the exercises performed at the target number of repetitions at their 8 repetition-maximum weight.

#### Testosterone and Placebo Gel Administration

Testosterone was provided in the form of a topical gel (generic 1.0% testosterone). The placebo gel was chemically identical to the testosterone gel, except that it did not contain testosterone. Testosterone and placebo gels were distributed to the clinical sites by a central pharmacy in identical pump-dispenser bottles, with labeling that allowed the sites to maintain blinding of participants and staff. The study site pharmacy was responsible for receiving, storing, weighing, distributing, and destroying (after return) the testosterone and placebo bottles.

Women assigned to the exercise plus topical testosterone gel group were prescribed a dose of testosterone gel that was expected to achieve a slightly supraphysiologic serum testosterone level (target total testosterone level: 110-160 ng/dL; reference range: 12-78 ng/dL). Serum testosterone levels were measured at 2 weeks and 1 month after baseline and then monthly thereafter; dose adjustments were individualized by the unblinded physician (D.R.) at the CCC. To maintain blinding among STEP-HI staff, each time an adjustment was necessary for an individual in the exercise plus topical testosterone gel group, the unblinded physician at the CCC gave instructions for an individual in the exercise plus placebo gel group to adjust the placebo dose (number of pumps). Participants received detailed oral and written instructions for proper application of the gel and completed daily logs to record the skin location and timing of each application. Gel bottles were returned to the study site pharmacy where they were weighed. Adherence to the gel was measured using serum testosterone levels and bottle weights.

#### Enhanced Usual Care Group 

The intent of the enhanced usual care group was to evaluate secondary aims of comparing changes in outcome measures between the enhanced usual care group and each of the exercise groups and to replicate a similar intervention that was conducted previously.^22^ Women assigned to the enhanced usual care group were prescribed a low-intensity home-based exercise program to perform independently. Participants received an in-person instructional session, written instructions, and descriptive photos. The exercise interventionist met with participants monthly to observe performance of the exercises and to provide guidance. Participants maintained a standardized exercise log that was returned monthly. Adherence was measured as the percentage of self-reported exercise sessions completed out of a possible 72 sessions.

Study site staff made weekly contact with participants in the enhanced usual care group by telephone or email to encourage adherence. Additionally, staff provided a monthly educational session that addressed health issues unrelated to exercise.

#### Calcium and Vitamin D Supplementation

Because of the high prevalence of vitamin D deficiency in this population,^23^ vitamin D supplements were provided to all participants in the form of vitamin D_3_ capsules, 2000 IU daily. Participants were also provided with calcium carbonate, 1000 mg daily, in divided doses. Calcium and/or vitamin D were not provided to women with contraindications identified by the study site physician.

### Sample Size and Power

Data from previous unpublished studies led us to estimate the projected mean (SD) changes in 6MWD of 42.7 (69.2) m in the exercise plus placebo gel group and 82.2 (92.5) m in the exercise plus topical testosterone gel group. Assuming a dropout rate of 20%, the sample size requirements were determined to be 100 per group with a power of 0.86 for an α = .05. Because of lower recruitment and higher retention than anticipated, with permission from the study’s Data and Safety Monitoring Board, we performed revised power computations (blinded to all outcome data) and determined that a sample size of 120 (50 participants in each exercise group, and 20 in the enhanced usual care group) would provide power of 0.64 for a 5% dropout rate and 0.62 for a 10% dropout rate and that a sample size of 168 (70 participants in each exercise group, and 28 in the enhanced usual care group) would provide power of 0.79 for a 5% dropout rate and 0.77 for a 10% dropout rate.

### Randomization

During the first 14 months of the STEP-HI trial, eligible women were randomly assigned to 1 of the 3 treatment groups in a 1:1:1 ratio. In April 2020, the study’s Data and Safety Monitoring Board approved a reduction in the target sample size to a minimum of 120 participants and modification of the ratio of women assigned to the enhanced usual care group because comparisons with that group were not the primary aim of the study. In the revised randomization scheme, for every 9 women randomized, 4 were assigned to exercise plus topical testosterone gel, 4 were assigned to exercise plus placebo gel, and 1 was assigned to enhanced usual care.

### Outcomes

Descriptions of all outcome measures are provided in the trial protocol^19^ (Supplement 1). All outcomes were measured by assessors blinded to the gels and exercise. The prespecified primary outcome was change in 6MWD from baseline to 24 weeks. Prespecified secondary outcomes included change in the following measures: Short Physical Performance Battery (SPPB) score (score range: 0-12, with the highest score indicating best performance and physical function), Modified Physical Performance Test score (score range: 0-36, with highest score indicating best performance and physical function), appendicular and total lean body mass by dual-energy x-ray absorptiometry, self-reported performance of instrumental and basic activities of daily living, Hip Rating Questionnaire score (score range: 0-100, with the highest score indicating best hip function), Patient-Reported Outcomes Measurement Information System global health score (physical health and mental health score ranges: 0-100, with the highest score indicating best possible state of health), and bone mineral density of the nonfractured femur.

### Safety Measures

The following measures were obtained from a physical examination and laboratory testing at baseline, 12 weeks, and 24 weeks: hemoglobin level, hematocrit level, liver function panel, lipid panel, and the Ferriman-Gallwey hirsutism scale score (score range: 0-36, with the highest score indicating severe hirsutism).^24^ Testosterone levels were obtained monthly. To monitor for evidence of malignant neoplasm, a mammogram and transvaginal or transabdominal ultrasonography were performed at 24 weeks. The occurrence of falls was documented by participants using daily logs that were collected monthly.

### Statistical Analysis

**Figure 1.**
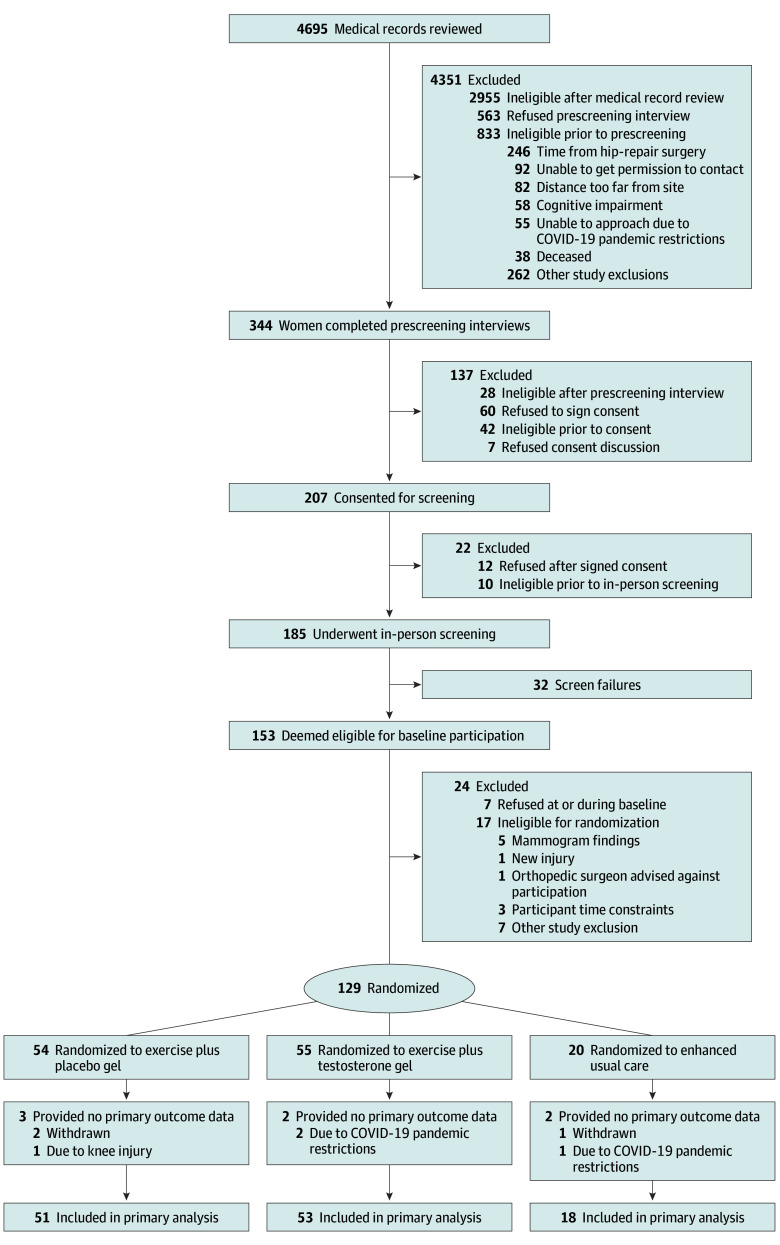
Trial Flow Diagram

Initial analyses included χ^2^, Fisher exact, and analysis of variance tests to compare baseline values of key demographic, clinical, and physiologic parameters. We restricted the analyses to participants who had baseline and at least 1 primary outcome measurement at 12 or 24 weeks (Figure 1).

The primary analysis used a modified intention-to-treat approach that excluded the 7 women who did not provide follow-up data. The analysis used a site-adjusted mixed-model analysis of variance that evaluated statistical contrasts to determine if the change from baseline to 24 weeks for exercise plus topical testosterone gel was significantly different from the change for exercise plus placebo gel. Additional contrasts were conducted to examine changes from 0 to 12 weeks and from 12 to 24 weeks. Covariance structures were selected based on best fit using bayesian information criteria and Akaike information criterion.^25,26^ Sensitivity analyses adjusted for baseline covariates, including 6MWD, body mass index (calculated as weight in kilograms divided by height in meters squared), Geriatric Depression Scale score (score range: 0-15, with the highest score indicating severe depression), fracture type, days since hip surgery, comorbidity index (total count of 33 items from a medical history questionnaire), lung disease, and age. The mixed-model approach was also applied to secondary outcomes.

Two-sided *P* < .05 indicated statistical significance. Data analyses were performed from November 2023 to November 2024 using SAS 9.4 (SAS Institute Inc).

## Results

### Participants

Between December 2018 and February 2023, 4695 medical records were reviewed, 344 women underwent telephone screening, and 185 underwent in-person screening. Recruitment ended after 129 participants were enrolled and randomized (55 to exercise plus topical testosterone gel, 54 to exercise plus placebo gel, 20 to enhanced usual care) (Figure 1) to allow sufficient time for completion of study procedures and preplanned analyses. Participants (Table 1) had a mean (SD) age of 79.3 (8.4) years and self-identified as Black or African American (6 [4.6%]), Hispanic or Latino (1 [0.8%]), White (120 [93.0%]), or multiracial (2 [1.6%]) individuals.

**Table 1.** Baseline Characteristics of Participants by Treatment Group

Characteristic	Participants, No. (%)		
	Exercise plus placebo gel (n = 54)	Exercise plus topical testosterone gel (n = 55)	Enhanced usual care (n = 20)
Demographic			
Age, mean (SD), y	78.3 (7.5)	79.4 (9.1)	83.0 (8.4)
Race^a^			
Black or African American	3 (5.6)	2 (3.6)	1 (5.0)
White	49 (90.7)	52 (94.6)	19 (95.0)
Multiracial	1 (1.9)	1 (1.8)	0
Other^a^	1 (1.9)	0	0
Hispanic or Latino ethnicity^a^	3 (5.6)	1 (1.8)	1 (5.0)
Married	15 (27.8)	18 (32.7)	3 (15.0)
Currently living alone	33 (61.1)	26 (47.3)	13 (65.0)
Place of residence in month prior to fracture			
House	36 (66.7)	36 (65.5)	14 (70.0)
Apartment, townhouse, or condominium	13 (24.1)	11 (20.0)	5 (25.0)
Retirement home or community	3 (5.6)	5 (9.1)	1 (5.0)
Other	2 (3.7)	3 (5.5)	0
Educational level			
<High school	3 (5.6)	1 (1.8)	0
High school diploma or GED	16 (29.6)	7 (12.7)	5 (25.0)
>High school diploma	35 (64.8)	47 (85.5)	15 (75.0)
Clinical			
Fracture type			
Intercapsular or femoral neck	22 (40.7)	25 (45.5)	3 (15.0)
Intertrochanteric	23 (42.6)	23 (41.8)	13 (65.0)
Subtrochanteric	9 (16.7)	7 (12.7)	4 (20.0)
Surgical repair type			
Hemi- or total hip arthroplasty	20 (37.0)	23 (41.8)	3 (15.0)
ORIF	34 (63.0)	32 (58.2)	17 (85.0)
Time from hip surgery to baseline, mean (SD), d	108.5 (33.4)	98.3 (32.5)	89.0 (21.6)
Time from hip surgery to randomization, mean (SD), wk	17.6 (5.0)	15.8 (4.5)	15.0 (3.0)
Serum total testosterone level, mean (SD), ng/dL	17.2 (12.5)	15.7 (9.2)	13.0 (5.8)
Serum vitamin D level, mean (SD), ng/mL	43.7 (15.1)	47.6 (16.6)	42.6 (20.6)
Prefracture IADL score, mean (SD)	11.3 (2.5)	11.5 (2.4)	11.4 (1.9)
Prefracture BADL score, mean (SD)	12.0 (2.0)	12.1 (1.7)	12.1 (1.8)
Use of assistive device to perform 6MWD	41 (75.9)	40 (72.7)	18 (90.0)
Comorbidities			
Hypertension	36 (66.7)	28 (50.9)	13 (65.0)
Arthritis	26 (48.2)	28 (50.9)	12 (60.0)
Heart disease	17 (31.5)	18 (32.7)	6 (30.0)
Lung disease	3 (5.6)	4 (7.3)	4 (20.0)
Diabetes	8 (14.8)	12 (21.8)	5 (25.0)
Short Blessed Test score, mean (SD)	1.9 (2.3)	1.8 (2.4)	2.3 (2.8)
Geriatric Depression Scale score, mean (SD)	2.4 (2.0)	1.8 (1.7)	3.2 (2.6)
Brief Resilience Scale score, mean (SD)	3.8 (0.6)	3.8 (0.7)	3.7 (0.7)
BMI, mean (SD)	26.9 (5.0)	25.6 (5.5)	24.4 (3.4)
Hip Rating Questionnaire pain rating item score	69.5 (10.3)	72.1 (12.4)	70.9 (11.9)
BMI			
<20	6 (11.1)	6 (10.9)	2 (10.0)
20 to <30	35 (64.8)	41 (74.6)	17 (85.0)
≥30	13 (21.1)	8 (14.6)	1 (5.0)
Taking calcium supplement	43 (79.6)	65 (83.6)	13 (65.0)
Taking vitamin D supplement	46 (85.2)	48 (87.3)	16 (80.0)
Taking medications for osteoporosis	7 (13.0)	11 (20.0)	4 (20.0)
Taking estrogen	2 (3.7)	3 (5.5)	0
Taking denosumab	0	3 (5.5)	2 (10.0)

Abbreviations: BADL, basic activities of daily living; BMI, body mass index (calculated as weight in kilograms divided by height in meters squared); GED, General Educational Development; IADL, instrumental activities of daily living; ORIF, open reduction and internal fixation; 6MWD, 6-Minute Walking Distance.

SI conversion factor: To convert testosterone to nanomoles per liter, multiply by 0.0347; vitamin D to nanomoles per liter, multiply by 2.496.

^a^ Participants self-reported their race and ethnicity. One participant did not specify race.

Among the 129 women randomized, 122 (94.6%) provided follow-up data for at least 1 time point and were included in the analysis. Among the 7 women who did not provide follow-up data (2 in exercise plus topical testosterone gel group, 3 in exercise plus placebo gel group, and 2 in enhanced usual care group), the reasons were COVID-19 pandemic restrictions (n = 3), unrelated leg injury (n = 1), withdrawal from the study (n = 1), and withdrawal by the site investigator (n = 2; refusal to use the gel [n = 1], and development of an eczematous rash from the gel [n = 1]).

### Receipt of Interventions and Exercise Fidelity

**Figure 2.**
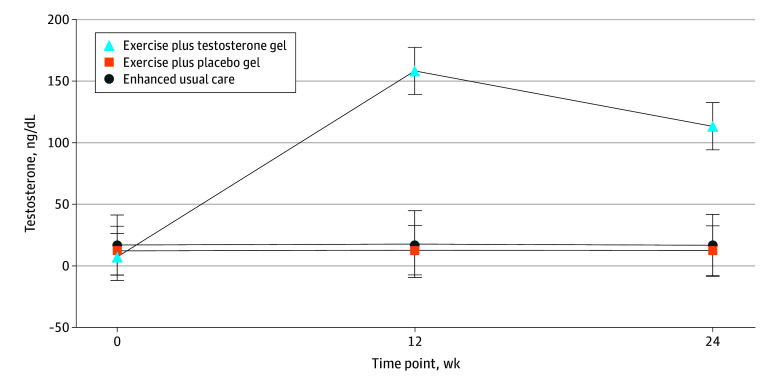
Serum Total Testosterone Levels at Baseline, 12 Weeks, and 24 Weeks Error bars represent 95% CIs.

Women assigned to the exercise plus placebo gel group attended a mean (SD) of 81.1% (18.7%) of the expected 48 sessions; those assigned to exercise plus topical testosterone gel attended a mean (SD) of 82.1% (18.7%) of the sessions. Adherence to prescribed sessions (attended ≥80%) was 64.2% (67 of 104) for the exercise groups combined and did not differ by group. Across all sites, 202 fidelity sessions were completed by supervising physical therapists. The mean (SD) score was 95.5% (3.6%) for phase 1 fidelity assessments (n = 53) and 93.8% (4.1%) for phase 2 fidelity assessments (n = 149). Women in the exercise plus topical testosterone gel group had a mean (SD) total testosterone level of 165.6 (18.3) ng/dL at 12 weeks after baseline and maintained their levels through 24 weeks after baseline (Figure 2).

### Primary and Secondary Outcomes

At 24 weeks, the mean (SD) increase in the 6MWD in the exercise plus topical testosterone gel group was 42.7 (8.2) m, 40.5 (8.4) m in the exercise plus placebo gel group, and 37.7 (14.8) m in the enhanced usual care group. These differences were not statistically significant (Table 2; eFigure 1 in Supplement 2). The confidence bounds on the 2.2-m between-group difference in the change in the 6MWD for exercise plus topical testosterone gel vs exercise plus placebo gel were −21.2 to 25.5 m. This range indicated that the potential magnitude of the effect on this measure was not clinically meaningful.

**Table 2.** Results of Mixed-Model Repeated-Measures Analyses for Site-Adjusted and Site Plus Covariate-Adjusted Models

Treatment groups compared	Mean (SEM) [95% CI]		Models, *P* value	
	Baseline	Change from baseline to 24 wk	Site adjusted^a^	Covariate adjusted^b^
**Outcome: 6MWD, m**				
Exercise plus topical testosterone gel vs exercise plus placebo gel				
Exercise plus topical testosterone gel	279.6 (15.9) [248.0 to 311.1]	42.7 (8.2) [26.3 to 59.0]	.85	.96
Exercise plus placebo gel	236.4 (16.5) [203.6 to 269.1]	40.5 (8.4) [23.8 to 57.2]		
Exercise plus topical testosterone gel vs enhanced usual care				
Exercise plus topical testosterone gel	279.6 (15.9) [248.0 to 311.1]	42.7 (8.2) [26.3 to 59.0]	.77	.63
Enhanced usual care	219.7 (20.9) [178.2 to 261.1]	37.7 (14.8) [8.3 to 67.0]		
Exercise plus placebo gel vs enhanced usual care				
Exercise plus placebo gel	236.4 (16.5) [203.6 to 269.1]	40.5 (8.4) [23.8 to 57.2]	.87	.66
Enhanced usual care	219.7 (20.9) [178.2 to 261.1]	37.7 (14.8) [8.3 to 67.0]		
**Outcome: Total testosterone level, ng/dL**				
Exercise plus topical testosterone gel vs exercise plus placebo gel				
Exercise plus topical testosterone gel	13.9 (2.0) [10.1 to 17.8]	105.1 (12.2) [81.0 to 129.2]	<.001	<.001
Exercise plus placebo gel	15.9 (2.0) [11.9 to 19.9]	0.7 (12.4) [−23.6 to 25.0]		
Exercise plus topical testosterone gel vs enhanced usual care				
Exercise plus topical testosterone gel	13.9 (2.0) [10.1 to 17.8]	105.1 (12.2) [81.0 to 129.2]	<.001	<.001
Enhanced usual care	12.9 (2.6) [7.8 to 18.0]	0.8 (21.6) [−41.8 to 43.4]		
Exercise plus placebo gel vs enhanced usual care				
Exercise plus placebo gel	15.9 (2.0) [11.9 to 19.9]	0.7 (12.4) [−23.6 to 25.0]	.95	.99
Enhanced usual care	12.9 (2.6) [7.8 to 18.0]	0.8 (21.6) [−41.8 to 43.4]		
**Outcome: Free testosterone level, pg/mL^c^**				
Exercise plus topical testosterone gel vs exercise plus placebo gel				
Exercise plus topical testosterone gel	0.3 (0) [0.2 to 0.3]	2.8 (0.9) [1.0 to 4.6]	.04	.001
Exercise plus placebo gel	0.2 (0) [0.2 to 0.3]	0 (1.0) [−2.0 to 2.0]		
Exercise plus topical testosterone gel vs enhanced usual care				
Exercise plus topical testosterone gel	0.3 (0) [0.2 to 0.3]	2.8 (0.9) [1.0 to 4.6]	.14	.006
Enhanced usual care	0.2 (0) [0.1 to 0.3]	0 (1.7) [−3.4 to 3.4]		
Exercise plus placebo gel vs enhanced usual care				
Exercise plus placebo gel	0.2 (0) [0.2 to 0.3]	0 (1.0) [−2.0 to 2.0]	.99	.99
Enhanced usual care	0.2 (0) [0.1 to 0.3]	0 (1.7) [−3.4 to 3.4]		
**Outcome: SPPB score**				
Exercise plus topical testosterone gel vs exercise plus placebo gel				
Exercise plus topical testosterone gel	7.0 (0.4) [6.2 to 7.8]	1.5 (0.2) [1.1 to 2.0]	.006	.009
Exercise plus placebo gel	6.7 (0.4) [5.8 to 7.5]	0.7 (0.2) [−0.3 to 1.1]		
Exercise plus topical testosterone gel vs enhanced usual care				
Exercise plus topical testosterone gel	7.0 (0.4) [6.2 to 7.8]	1.5 (0.2) [1.1 to 2.0]	.86	.78
Enhanced usual care	5.5 (0.5) [4.4 to 6.6]	1.5 (0.4) [0.7 to 2.2]		
Exercise plus placebo gel vs enhanced usual care				
Exercise plus placebo gel	6.7 (0.4) [5.8 to 7.5]	0.7 (0.2) [−0.3 to 1.1]	.09	.12
Enhanced usual care	5.5 (0.5) [4.4 to 6.6]	1.5 (0.4) [0.7 to 2.2]		
**Outcome: MPPT score**				
Exercise plus topical testosterone gel vs exercise plus placebo gel				
Exercise plus topical testosterone gel	21.8 (1.2) [19.4 to 24.2]	3.3 (0.6) [2.2 to 4.4]	.14	.23
Exercise plus placebo gel	21.4 (1.3) [18.9 to 23.9]	2.1 (0.6) [1.0 to 3.2]		
Exercise plus topical testosterone gel vs enhanced usual care				
Exercise plus topical testosterone gel	21.8 (1.2) [19.4 to 24.2]	3.3 (0.6) [2.2 to 4.4]	.76	.65
Enhanced usual care	18.6 (1.6) [15.4 to 21.7]	3.0 (1.0) [1.0 to 5.0]		
Exercise plus placebo gel vs enhanced usual care				
Exercise plus placebo gel	21.4 (1.3) [18.9 to 23.9]	2.1 (0.6) [1.0 to 3.2]	.46	.68
Enhanced usual care	18.6 (1.6) [15.4 to 21.7]	3.0 (1.0) [1.0 to 5.0]		
**Outcome: Maximum leg press, 1-repetition, lbs**				
Exercise plus topical testosterone gel vs exercise plus placebo gel				
Exercise plus topical testosterone gel	93.5 (9.1) [75.4 to 111.6]	62.6 (7.1) [48.5 to 76.7]	.95	.63
Exercise plus placebo gel	85.7 (9.5) [66.8 to 104.5]	61.9 (7.4) [47.3 to 76.9]		
Exercise plus topical testosterone gel vs enhanced usual care				
Exercise plus topical testosterone gel	93.5 (9.1) [75.4 to 111.6]	62.6 (7.1) [48.5 to 76.7]	.09	.07
Enhanced usual care	91.7 (12.3) [67.2 to 116.1]	38.2 (12.6) [13.2 to 63.2]		
Exercise plus placebo gel vs enhanced usual care				
Exercise plus placebo gel	85.7 (9.5) [66.8 to 104.5]	61.9 (7.4) [47.3 to 76.9]	.11	.15
Enhanced usual care	91.7 (12.3) [67.2 to 116.1]	38.2 (12.6) [13.2 to 63.2]		
**Outcome: Handgrip strength, left hand, kg**				
Exercise plus topical testosterone gel vs exercise plus placebo gel				
Exercise plus topical testosterone gel	16.5 (0.9) [14.6 to 18.3]	0.6 (0.5) [−0.3 to 1.6]	.54	.57
Exercise plus placebo gel	17.0 (1) [15.0 to 18.9]	1.1 (0.5) [0.1 to 2.0]		
Exercise plus topical testosterone gel vs enhanced usual care				
Exercise plus topical testosterone gel	16.5 (0.9) [14.6 to 18.3]	0.6 (0.5) [−0.3 to 1.6]	.89	.94
Enhanced usual care	16.7 (1.3) [14.1 to 19.2]	0.8 (0.8) [−0.9 to 2.4]		
Exercise plus placebo gel vs enhanced usual care				
Exercise plus placebo gel	17.0 (1.0) [15.0 to 18.9]	1.1 (0.5) [0.1 to 2.0]	.77	.74
Enhanced usual care	16.7 (1.3) [14.1 to 19.2]	0.8 (0.8) [−0.9 to 2.4]		
**Outcome: Handgrip strength, right hand, kg**				
Exercise plus topical testosterone gel vs exercise plus placebo gel				
Exercise plus topical testosterone gel	17.0 (1.0) [15.0 to 19.0]	0.3 (0.5) [−0.7 to 1.3]	.56	.69
Exercise plus placebo gel	18.1 (1.0) [16.0 to 20.2]	0.7 (0.5) [−0.3 to 1.8]		
Exercise plus topical testosterone gel vs enhanced usual care				
Exercise plus topical testosterone gel	17.0 (1.0) [15.0 to 19.0]	0.3 (0.5) [−0.7 to 1.3]	.78	.94
Enhanced usual care	17.9 (1.4) [15.1 to 20.6]	0 (0.9) [−1.8 to 1.8]		
Exercise plus placebo gel vs enhanced usual care				
Exercise plus placebo gel	18.1 (1.0) [16.0 to 20.2]	0.7 (0.5) [−0.3 to 1.8]	.50	.71
Enhanced usual care	17.9 (1.4) [15.1 to 20.6]	0 (0.9) [−1.8 to 1.8]		
**Outcome: Appendicular lean body mass, kg**				
Exercise plus topical testosterone gel vs exercise plus placebo gel				
Exercise plus topical testosterone gel	16.3 (0.5) [15.3 to 17.3]	0.4 (0.1) [0.2 to 0.7]	.93	.87
Exercise plus placebo gel	17.1 (0.5) [16.0 to 18.1]	0.4 (0.1) [0.1 to 0.7]		
Exercise plus topical testosterone gel vs enhanced usual care				
Exercise plus topical testosterone gel	16.3 (0.5) [15.3 to 17.3]	0.5 (0.1) [0.2 to 0.7]	.07	.02
Enhanced usual care	15.5 (0.7) [14.1 to 16.9]	−0.1 (0.3) [−0.7 to 0.4]		
Exercise plus placebo gel vs enhanced usual care				
Exercise plus placebo gel	17.1 (0.5) [16.0 to 18.1]	0.4 (0.1) [0.1 to 0.7]	.08	.03
Enhanced usual care	15.5 (0.7) [14.1 to 16.9]	−0.1 (0.3) [−0.7 to 0.4]		
**Outcome: Total lean body mass, kg**				
Exercise plus topical testosterone gel vs exercise plus placebo gel				
Exercise plus topical testosterone gel	37.9 (1.0) [36.0 to 39.7]	0.9 (0.3) [0.4 to 1.4]	.34	.25
Exercise plus placebo gel	39.1 (1.0) [37.2 to 41.1]	0.6 (0.3) [0.0 to 1.1]		
Exercise plus topical testosterone gel vs enhanced usual care				
Exercise plus topical testosterone gel	37.9 (1.0) [36.0 to 39.7]	0.9 (0.3) [0.4 to 1.4]	.09	.07
Enhanced usual care	36.0 (1.3) [33.5 to 38.5]	0.0 (1.1) [−0.1 to 0.9]		
Exercise plus placebo gel vs enhanced usual care				
Exercise plus placebo gel	39.1 (1.0) [37.2 to 41.1]	0.6 (0.3) [0.0 to 1.1]	.30	.33
Enhanced usual care	36.0 (1.3) [33.5 to 38.5]	0.0 (1.1) [−0.1 to 0.9]		
**Outcome: Femoral hip BMD**				
Exercise plus topical testosterone gel vs exercise plus placebo gel				
Exercise plus topical testosterone gel	0.7 (0.02) [0.7 to 0.8]	−0.0 (0.0) [−0.1 to 0.0]	.82	.70
Exercise plus placebo gel	0.7 (0.0) [0.7 to 0.8]	−0.0 (0.0) [−0.1 to 0.1]		
Exercise plus topical testosterone gel vs enhanced usual care				
Exercise plus topical testosterone gel	0.7 (0.0) [0.7 to 0.8]	−0.0 (0.0) [−0.1 to 0.0]	.79	.80
Enhanced usual care	0.7 (0.0) [0.7 to 0.8]	−0.0 (0.0) [−0.1 to 0.1]		
Exercise plus placebo gel vs enhanced usual care				
Exercise plus placebo gel	0.7 (0.0) [0.7 to 0.8]	−0.0 (0.0) [−0.1 to 0.1]	.67	.99
Enhanced usual care	0.7 (0.0) [0.7 to 0.8]	−0.0 (0.0) [−0.1 to 0.1]		
**Outcome: BADL total score**				
Exercise plus topical testosterone gel vs exercise plus placebo gel				
Exercise plus topical testosterone gel	12.2 (0.3) [11.6 to 12.8]	0.6 (0.2) [0.3 to 0.9]	.33	.41
Exercise plus placebo gel	12.0 (0.3) [11.4 to 12.7]	0.4 (0.2) [0.0 to 0.7]		
Exercise plus topical testosterone gel vs enhanced usual care				
Exercise plus topical testosterone gel	12.2 (0.3) [11.6 to 12.8]	0.6 (0.2) [0.3 to 0.9]	.59	.53
Enhanced usual care	12.2 (0.4) [11.3 to 13.0]	0.8 (0.3) [0.2 to 1.4]		
Exercise plus placebo gel vs enhanced usual care				
Exercise plus placebo gel	12 (0.3) [11.4 to 12.7]	0.4 (0.2) [0.0 to 0.7]	.21	.22
Enhanced usual care	12.2 (0.4) [11.3 to 13.0]	0.8 (0.3) [0.2 to 1.4]		
**Outcome: IADL total score**				
Exercise plus topical testosterone gel vs exercise plus placebo gel				
Exercise plus topical testosterone gel	11.9 (0.4) [11.1 to 12.7]	0.4 (0.2) [−0.1 to 0.8]	.69	.70
Exercise plus placebo gel	11.7 (0.4) [10.9 to 12.5]	0.5 (0.2) [0.1 to 1.0]		
Exercise plus topical testosterone gel vs enhanced usual care				
Exercise plus topical testosterone gel	11.9 (0.4) [11.1 to 12.7]	0.4 (0.2) [−0.1 to 0.8]	.33	.40
Enhanced usual care	11.7 (0.5) [10.6 to 12.7]	0.8 (0.4) [0.1 to 1.5]		
Exercise plus placebo gel vs enhanced usual care				
Exercise plus placebo gel	11.7 (0.4) [10.9 to 12.5]	0.5 (0.2) [0.1 to 1.0]	.49	.14
Enhanced usual care	11.7 (0.5) [10.6 to 12.7]	0.8 (0.4) [0.1 to 1.5]		
**Outcome: FSQ score**				
Exercise plus topical testosterone gel vs exercise plus placebo gel				
Exercise plus topical testosterone gel	23.9 (0.9) [22.0 to 25.7]	2.0 (0.7) [0.7 to 3.3]	.60	.64
Exercise plus placebo gel	22.5 (1.0) [20.6 to 24.4]	1.6 (0.7) [0.2 to 2.9]		
Exercise plus topical testosterone gel vs enhanced usual care				
Exercise plus topical testosterone gel	23.9 (0.9) [22.0 to 25.7]	2.0 (0.7) [0.7 to 3.3]	.45	.44
Enhanced usual care	21.6 (1.3) [19.1 to 24.1]	3.0 (1.1) [0.8 to 5.2]		
Exercise plus placebo gel vs enhanced usual care				
Exercise plus placebo gel	22.5 (1) [20.6 to 24.4]	1.6 (0.7) [0.2 to 2.9]	.26	.27
Enhanced usual care	21.6 (1.3) [19.1 to 24.1]	3.0 (1.1) [0.8 to 5.2]		
**Outcome: PROMIS physical health *t* score**				
Exercise plus topical testosterone gel vs exercise plus placebo gel				
Exercise plus topical testosterone gel	49.7 (1.2) [47.4 to 51.9]	1.9 (0.8) [0.2 to 3.5]	.60	.64
Exercise plus placebo gel	47.9 (1.2) [45.6 to 50.3]	1.3 (0.9) [−0.4 to 2.9]		
Exercise plus topical testosterone gel vs enhanced usual care				
Exercise plus topical testosterone gel	49.7 (1.2) [47.4 to 51.9]	1.9 (0.8) [0.2 to 3.5]	.18	.24
Enhanced usual care	45.4 (1.5) [42.4 to 48.4]	4.1 (1.4) [1.3 to 6.9]		
Exercise plus placebo gel vs enhanced usual care				
Exercise plus placebo gel	47.9 (1.2) [45.6 to 50.3]	1.3 (0.9) [−0.4 to 2.9]	.09	.13
Enhanced usual care	45.4 (1.5) [42.4 to 48.4]	4.1 (1.4) [1.3 to 6.9]		
**Outcome: PROMIS mental health *t* score**				
Exercise plus topical testosterone gel vs exercise plus placebo gel				
Exercise plus topical testosterone gel	52.0 (1.2) [49.7 to 54.3]	1.6 (0.9) [−0.2 to 3.3]	.08	.08
Exercise plus placebo gel	51.1 (1.2) [48.8 to 53.5]	0.8 (0.9) [−2.6 to 1.1]		
Exercise plus topical testosterone gel vs enhanced usual care				
Exercise plus topical testosterone gel	52.0 (1.2) [49.7 to 54.3]	1.6 (0.9) [−0.2 to 3.3]	.98	.78
Enhanced usual care	47.8 (1.6) [44.7 to 50.9]	1.6 (1.5) [−1.4 to 4.6]		
Exercise plus placebo gel vs enhanced usual care				
Exercise plus placebo gel	51.1 (1.2) [48.8 to 53.5]	0.8 (0.9) [−2.6 to 1.1]	.19	.12
Enhanced usual care	47.8 (1.6) [44.7 to 50.9]	1.6 (1.5) [−1.4 to 4.6]		
**Outcome: Hip Rating Questionnaire score**				
Exercise plus topical testosterone gel vs exercise plus placebo gel				
Exercise plus topical testosterone gel	73.2 (2.1) [69.2 to 77.3]	8.5 (1.5) [5.6 to 11.4]	.65	.68
Exercise plus placebo gel	71.0 (2.1) [66.8 to 75.2]	7.6 (1.5) [4.6 to 10.5]		
Exercise plus topical testosterone gel vs enhanced usual care				
Exercise plus topical testosterone gel	73.2 (2.1) [69.2 to 77.3]	8.5 (1.5) [5.6 to 11.4]	.39	.39
Enhanced usual care	71.1 (2.9) [65.4 to 76.7]	6.0 (2.5) [1.1 to 10.9]		
Exercise plus placebo gel vs enhanced usual care				
Exercise plus placebo gel	71.0 (2.1) [66.8 to 75.2]	7.6 (1.5) [4.6 to 10.5]	.60	.59
Enhanced usual care	71.1 (2.9) [65.4 to 76.7]	6.0 (2.5) [1.1 to 10.9]		

Abbreviations: BADL, basic activities of daily living; BMD, bone mineral density; FSQ, Functional Status Questionnaire (score range: 0-36, with the highest score indicating complete independence in performance of daily activities); IADL, instrumental activities of daily living; MPPT, Modified Physical Performance Test (score range: 0-36, with highest score indicating best performance and physical function); PROMIS, Patient-Reported Outcomes Measurement Information System (physical health and mental health score ranges: 0-100, with the highest score indicating best possible state of health); 6MWD, 6-Minute Walking Distance; SPPB, Short Physical Performance Battery (score range: 0-12, with the highest score indicating best performance and physical function).

^a^ Site-adjusted models accounted for clustering within site.

^b^ Site plus covariate-adjusted models included baseline level of each outcome, baseline 6MWD, body mass index, general depression scale score, fracture type, days since hip surgery, comorbidity index, lung disease, and participant age.

^c^ Free testosterone level was collected for 86 participants.

At 24 weeks, there was a statistically significant difference in the change in SPPB score between exercise plus topical testosterone gel and exercise plus placebo gel groups (1.5 [0.2] vs 0.7 [0.2]); the difference in the changes was 0.8 (0.3; *P* = .009). The differences between the changes in SPPB score in the enhanced usual care group with both exercise groups were not statistically significant (Table 2; eFigure 2 in Supplement 2). Changes in the other 9 secondary outcome measures were not statistically significant. The confidence bounds on the 0.83 between-group difference in the change in SPPB score for exercise plus topical testosterone gel vs exercise plus placebo gel were 0.21 to 1.44.

In a post hoc analysis of assistive device use among participants who required a walker or cane at baseline to perform the 6MWD, 15 of 38 (39.5%) in the exercise plus topical testosterone gel group did not require an assistive device at 24 weeks compared with 7 of 41 (17.1%) in the exercise plus placebo gel group and 3 of 17 (17.7%) in the enhanced usual care group (*P* = .053 for 3-way comparison; *P* = .03 for exercise plus placebo gel vs exercise plus topical testosterone gel).

### Safety

There were 11 participants (8.5%) who had 1 or more serious adverse events during the intervention period, with 7 (12.7%) in the exercise plus topical testosterone gel group, 3 (5.6%) in the exercise plus placebo gel group, and 1 (5.0%) in the enhanced usual care group (*P* = .48) (Table 3). The number of serious adverse events per participant and the severity, relatedness, and expectedness of the events were similar between the 3 groups. The most commonly reported serious adverse event was falls, which also did not differ by group (eTable 1 in Supplement 2). There was 1 unrelated death (1.8%) that occurred in the exercise plus topical testosterone gel group; the participant provided follow-up data at 12 weeks and therefore was included in the primary analysis. There were no significant group differences in the changes in hemoglobin levels, serum lipids or liver function tests, or in Ferriman-Gallwey scores (eTables 2-5 in Supplement 2). At 24 weeks, mammograms did not reveal significant findings in either exercise group. One participant in the exercise plus placebo gel group had endometrial hypertrophy.

**Table 3.** Participants With Serious Adverse Events by Treatment Group

Characteristic	Participants, No. (%)		
	Exercise plus placebo gel (n = 54)	Exercise plus topical testosterone gel (n = 55)	Enhanced usual care (n = 20)
Participants with SAE^a^	3 (5.6)	7 (12.7)	1 (5.0)
No. of SAEs per participant			
1	1 (1.9)	4 (7.3)	1 (5.0)
2	2 (3.7)	2 (3.6)	0
≥3	0	1 (1.8)	0
SAE severity^b^			
Moderate	0	2 (3.6)	0
Severe	3 (5.6)	4 (7.3)	1 (5.0)
Fatal	0	1 (1.8)	0
SAE relatedness^c^			
Not related	2 (3.7)	3 (5.5)	1 (5.0)
Possibly related	1 (1.9)	4 (7.3)	0
SAE expectedness^d^			
Unexpected	0	3 (5.5)	0
Expected	3 (5.6)	4 (7.3)	1 (5.0)

Abbreviation: SAE, serious adverse events.

^a^ *P* > .05.

^b^ Severity was classified according to the most severe adverse event.

^c^ Relatedness was classified according to significant relatedness status, with not related as least significant, possibly related as more significant, and definitely related as most significant.

^d^ Expectedness was classified according to significant expectedness status, with unexpected as least significant and expected as most significant.

## Discussion

In this multicenter STEP-HI trial including older women with a recent hip fracture repair and persistent functional impairments, 24 weeks of supervised exercise combined with testosterone gel did not result in significant improvements in 6MWD compared with supervised exercise plus placebo gel. In secondary analyses, the change in physical performance as measured by the SPPB was significantly greater for women in the exercise plus topical testosterone gel group than women in the exercise plus placebo gel group. There were no other significant improvements in secondary outcome measures. Our findings do not support the hypothesis that supervised exercise plus testosterone gel improves endurance or long-distance mobility in older women after a hip fracture.

While the a priori sample size calculations were powered to detect a clinically meaningful effect size for the change in the 6MWD, the observed effect size comparing the 2 exercise groups was more modest (2.2 m; 95% CI, −21.2 to 25.5 m). These confidence bounds mean that we are 95% certain that the difference in efficacy between exercise plus placebo gel and exercise plus topical testosterone gel is at most 25.5 m. Since a large clinically meaningful difference is approximately 50 m,^27,28^ this finding establishes with a high degree of confidence that there is not a large clinically important benefit associated with adding testosterone to exercise insofar as the primary outcome is concerned. The size of the enhanced usual care group was reduced because that group was not involved in the primary hypothesis of the STEP-HI trial. Because that sample size reduction resulted in larger SEs, we were unable to draw definitive conclusions from comparisons with that group.

There are several potential explanations for the null findings regarding the primary hypothesis. First, the 24-week treatment period, the target testosterone level, and/or the timing of the interventions may have been insufficient to induce the hypothesized changes in muscle strength or function necessary to improve long-distance walking.^29^ Second, the anabolic effects of testosterone may not have been strong enough to exceed the improvements in long-distance walking achieved through exercise training alone. The greater improvement in SPPB score in the exercise plus topical testosterone gel group compared with the exercise plus placebo gel group (increase of 0.8) is within the range considered to be clinically meaningful^28,30^ and suggests an independent effect of testosterone therapy on physical performance. The finding that participants assigned to testosterone therapy reduced their use of assistive devices also suggests a potential benefit for select populations of patients with hip fracture requiring devices. The improvements in SPPB score and assistive device use suggest that testosterone therapy may have more benefits for strength and functional movements rather than endurance activities, such as the 6MWD.

In addition to the null testosterone effects, no significant differences in outcomes were observed between exercise plus topical testosterone gel and exercise plus placebo gel compared with enhanced usual care. Because this was not the primary comparison of interest, the revised sample size of only 20 participants in the enhanced usual care group may not have been sufficient to permit the reliable testing of enhanced usual care–related hypotheses. The intensity of the supervised intervention in this study was similar to that in a prior trial,^22^ participants in the exercise group demonstrated high adherence and retention in the study, and the exercise interventionists were rigorously monitored with a high degree of fidelity to the protocol.

### Strengths and Limitations

This trial had several strengths. To our knowledge, it was the first randomized clinical trial of exercise and testosterone therapy conducted in older women with mobility limitations, which is an important target population for anabolic interventions. We were able to enroll women with a high burden of multimorbidity and functional impairment while maintaining high adherence and fidelity to a complex intervention. The testosterone therapy protocol was well tolerated, and there were no safety concerns related to gel treatment over the 24-week treatment period.

This trial had several limitations. Because the enhanced usual care group protocol included low-intensity exercises, participants may have exercised more than expected. Second, the results are not generalizable to the immediate recovery period after hip fracture repair or to patients with hip fracture who had more or less functional impairment, those with dementia, or those in racially and ethnically minoritized populations. Third, while the observed significant improvement in SPPB score in the exercise plus topical testosterone gel group compared with exercise plus placebo gel group is important, the finding should be interpreted with caution and suggests a need for future studies.

## Conclusions

Among older women with a recent hip fracture repair and persistent mobility impairments, exercise training combined with topical testosterone therapy did not result in improvements in 6MWD compared with exercise training alone, although improvements in physical performance were observed. These findings do not support prescribing testosterone therapy to women to enhance long-distance walking mobility after hip fracture. However, testosterone combined with exercise might benefit physical performance and mobility for short distances and warrants further study.

## Notes

Supplement 1.
Trial Protocol

Supplement 2. **eFigure 1.** Six-Minute Walk Distance at Baseline, Week 12, and Week 24 **eFigure 2.** Short Physical Performance Battery (SPPB) Score at Baseline, Week 12, and Week 24 **eTable 1.** Participants With Adverse Events from Falls by Treatment Group **eTable 2.** List of Serious Adverse Events by System Organ Class and Preferred Term by Treatment Group **eTable 3.** List of Serious Adverse Events by System Organ Class and Preferred Term by Severity **eTable 4.** Laboratory Test Results by Study Visit and Treatment Group **eTable 5.** Ferriman-Gallwey Hirsutism Scores by Study Visit and Treatment Group

Supplement 3.
Data Sharing Statement
