# Errors in the Author Affiliations

**DOI:** 10.1001/jamanetworkopen.2025.19679

**Published:** 2025-06-23

In the Original Investigation titled “Combining Exercise Training and Testosterone Therapy in Older Women After Hip Fracture: The STEP-HI Randomized Clinical Trial,”^1^ which was published on May 15, 2025, the affiliations for Dr Binder and Dr Magaziner were incorrect. Dr Binder is affiliated with the Division of General Medicine and Geriatrics, Washington University School of Medicine in St Louis, St Louis, Missouri. Dr Magaziner is affiliated with the Department of Epidemiology and Public Health, University of Maryland School of Medicine, Baltimore. This article was corrected.^1^
