# Supplementary material for: Combining Exercise Training and Testosterone Therapy in Older Women After Hip Fracture: The STEP-HI Randomized Clinical Trial
Source: JAMA Netw Open. 2025 May 15;8(5):e2510512. doi: 10.1001/jamanetworkopen.2025.10512 (PMC12082367; doi:10.1001/jamanetworkopen.2025.10512)
Supplement: Supplement 1. — Trial Protocol [file jamanetwopen-e2510512-s001.pdf]

Combining Testosterone Therapy and Exercise to Improve Function Post Hip Fracture

STEP-HI Project
Starting a **T**estosterone and **E**xercise **P**rogram after **H**ip **I**njury

IND - 066697
IRB - 201609077
Clinical Trials.gov – NCT02938923

Study Protocol
Version 12.5
November 15, 2022

Contact PI
Ellen F. Binder, MD
Washington University School of Medicine

Table of Contents

Acronyms Dictionary 9

Section 1: Executive Summary 11

Section 2: Study PIs and Participating Clinical Sites 13

- **2.1 STEP-HI PIs 13**
- **2.2 Participating Clinical Sites 13**

Section 3: Study Organization & Administration 16

- **3.1 Study Chair 17**
- **3.2 National Institute on Aging (NIA) 17**
- **3.3 Data and Safety Monitoring Board (DSMB) 17**
- **3.4 Executive Committee (EC) 18**
- **3.5 Steering Committee (SC) 18**
- **3.6 Clinical Coordinating Center (CCC) 19**
  - 3.6.1 CCC Oversight and Monitoring Responsibilities 19
  - 3.6.2 CCC Intervention Training and Fidelity Monitoring 20
  - 3.6.3 Ancillary Studies Committee 20
- **3.7 Data Coordinating Center (DCC) 20**
  - 3.7.1 Publications Committee 21
- **3.8 Single Institutional Review Board (sIRB) 21**
- **3.9 Central Pharmacy Services 21**
- **3.10 Centralization of Laboratory Testing 21**
- **3.11 Central DXA Imaging Center 21**
- **3.12 Clinical Sites 21**

Section 4: Study Objectives 23

- **4.1 Primary Objective 23**
- **4.2 Secondary Objectives 23**
- **4.3 Exploratory Objectives 23**

Section 5: Background 24

- **5.1 Consequences of Hip Fracture 24**
- **5.2 Rehabilitation and Exercise Strategies after Hip Fracture 24**
- **5.3 Decrements in Muscle Mass and Strength after Hip Fracture 25**
- **5.4 Testosterone Deficiency in the Elderly and in Hip Fracture Patients 25**
- **5.5 Rationale for the Interventions 26**
  - 5.5.1 Androgen Replacement 26

5.5.2 Rationale for targeting female hip fracture patients 28
5.5.3 Rationale for a 3-Group Design....................................28
5.5.4 Rationale for a 6-month intervention period 28
5.5.5 Preliminary Data/Efficacy and Safety of Testosterone Replacement after Hip Fracture 28
5.5.6 Safety Measures in Pilot Study 29
5.5.7 Addressing Safety Concerns....................................29
Section 6: Study Design....................................31
6.1 Overview of Study Design....................................31
6.2 Screening 32
6.3 Randomization 32
6.4 Interventions 32
6.5 Source Documentation....................................33
6.6 Efficacy and Safety Measures....................................33
6.7 Blinding of Study Staff 33
Section 7: Eligibility 35
7.1 Inclusion Criteria....................................35
7.2 Exclusion Criteria 35
7.3 Concomitant Medications 36
7.3.1 Prohibited medications....................................36
7.3.2 Pharmacologic Agents for Osteoporosis 37
Section 8: Recruitment and Retention 38
8.1 Identification of Hip Fracture Patients....................................38
8.2 Participant Recruitment and Pre-Screening 38
8.3 Informed Consent 39
8.4 Enrollment....................................39
8.5 Screening 40
8.6 Retention Promotion Efforts 40
8.7 Drop-out Prevention Efforts....................................41
8.8 Premature Withdrawal from the Study 41
8.9 Replacement of Subjects 42
8.10 Monitoring Recruitment and Retention 42
8.10.1 Monitoring and Quality Control of Recruitment and Retention....................................42
Section 9: Study Measures 43
9.1 Physical and Questionnaire Measures....................................43

9.1.1 Demographics....43
9.1.2 Medical History....43
9.1.3 Medications or Medication Updates....43
9.1.4 Height, Weight, and Vital Signs....43
9.1.5 Physical Examination....43
9.1.6 Short Blessed Test (SBT)....43
9.1.7 Activities of Daily Living (ADLs)....44
9.1.8 Geriatric Depression Scale – Short Form (GDS-SF)....44
9.1.9 Seattle Angina Questionniare-7 (SAQ-7)....44
9.1.10 Hip Rating Questionnaire (HRQ)....44
9.1.11 PROMIS® Global Health....44
9.1.12 Brief Resilience Scale (BRS)....45
9.1.13 Ferriman-Gallwey Scale....45
9.2 Physical Performance Measures....45
9.2.1 Six Minute Walking Distance (Primary Outcome Measure)....45
9.2.2 Leg Press 1-Repetition Max Test....45
9.2.3 Modified Physical Performance Test (mPPT)....46
9.2.4 Short Physical Performance Battery (SPPB)....46
9.2.5 Hand Grip Strength....46
9.3 Other Procedures....46
9.3.1 Laboratory Blood Tests....46
9.3.2 Electrocardiogram (ECG)....46
9.3.3 Dual Energy X-Ray Absorptiometry (DXA)....47
9.3.4 Mammogram....47
9.3.5 Transvaginal Ultrasound....47
Section 10: Assessment Schedule....48
Table 2: Schedule of Assessments....48
10.1 Screening Assessments....49
10.2 Baseline Assessments....49
10.3 Follow-up Assessments....50
10.4 Order of Study Procedures....51
Section 11: Standard Care Procedures for All Participants....52
11.1 Dietary Assessments....52
11.2 Vitamin D_3 and Calcium Supplementation....52
11.3 Physical Therapy Evaluation....53

Section 12: Randomization **54**
Section 13: Enhanced Usual Care (EUC) Group Activities **55**
13.1 Home Exercise Program **55**
13.1.1 Home Exercise Adherence 55
13.2 Health Education Sessions **55**
Section 14: Supervised Exercise Training Group Interventions **56**
14.1 Physical Therapy Exercises (Phase 1) **57**
14.2 Progressive Resistance Training (Phase 2) **57**
14.3 Remote Supervised Exercise Sessions **58**
14.4 Home Maintenance Exercise Prescription **58**
14.5 Scheduling Exercise Intervention Visits **58**
14.5.1 Missed Exercise Sessions 58
14.6 Adherence Measurements **59**
14.7 Fidelity Plan for Exercise Interventions **59**
14.7.1 Initial Training and Certification for Procedural Reliability 59
14.7.2 Ensuring Ongoing Competency 60
Section 15: Testosterone/Placebo Intervention **61**
15.1 Drug Distribution **61**
15.2 Dosing and Schedule **61**
15.3 Dose Adjustments **62**
15.3.1 Adverse Events and Symptom Monitoring 62
15.3.2 Medication Adherence 62
15.3.3 Fidelity Plan for Study Drug Procedures 63
Section 16: Safety Considerations **64**
16.1 Pre-Intervention Safety Screening **64**
16.2 Safety Considerations for Study Assessments **64**
16.3 Safety Considerations for Testosterone Therapy **64**
16.3.1 Evaluation of Hirsutism (Ferriman-Gallwey scale) 64
16.3.2 Adverse Event Assessment 64
16.3.3 Laboratory Testing 64
16.3.4 Transvaginal Ultrasound 65
16.3.5 Mammogram 65
16.4 Safety Considerations for Supervised Resistance Training Program **65**
16.5 Safety Considerations for Remote Exercise Program **66**
16.6 Safety Considerations for Home Exercise Program **66**

16.7 Participant Education about Potential Risks **66**

Section 17: Adverse Event Reporting **67**

17.1 Definitions **67**

17.2 Categorizing an Adverse Event **69**

17.2.1 Expectedness 69

17.2.2 Relatedness 71

17.2.3 Severity 71

17.3 Adverse Event Recording **71**

17.4 Adverse Event Reporting **72**

17.5 Emergency Unblinding **72**

17.6 Adverse Event Monitoring **72**

17.6.1 CCC Monitoring 72

17.6.2 Internal Safety Monitor (ISM) 72

17.6.3 Independent Data and Safety Monitoring Board (DSMB) 73

Section 18: Discontinuation **74**

18.1 Study Drug Discontinuation **75**

18.2 Study Exercise Intervention Discontinuation **75**

18.3 Early Withdrawal Visit **75**

Section 19: Sample Size and Statistical Power **76**

Section 20: Statistical Analysis **79**

20.1 Specific Aim 1 ..... **79**

20.2 Specific Aim 2 ..... **79**

20.3 Specific Aim 3 ..... **80**

Section 21: Clinical Coordinating Center (CCC) Activities **81**

21.1 Study Protocol, Manual of Procedures, and Informed Consent **81**

21.1.1 Study Protocol 81

21.1.2 Manual of Procedures 81

21.1.3 Informed Consent 81

21.2 Clinical Site Coordination **81**

21.2.1 Budgets 81

21.2.2 Regulatory 81

21.3 Clinical Site Visits **81**

21.3.1 Site Initiation 81

21.3.2 Site Monitoring 82

21.4 Communication with Clinical Sites **82**

21.5 Reportable Adverse Event (RAE) Reporting 82
21.6 Meetings and Training Sessions 82
21.6.1 Investigator Meeting 82
21.6.2 Steering Committee and DSMB Calls 82
21.6.3 Training Sessions 83
21.6.4 Clinical Site Conference Calls 83
21.7 Study Equipment and Website 83
21.8 Collaboration with the Data Coordinating Center 83
21.9 Central Lab, Pharmacy, and DXA Coordination 83
Section 22: Data Coordinating Center (DCC) Activities 84
22.1 DCC Responsibilities during the Study Start-Up Period 84
22.1.1 Forms Design 84
22.1.2 Training and Certifying Personnel 84
22.2 Data Management 85
22.2.1 The REDCap System 85
22.2.2 Testing the Data Management System at the DCC 85
22.2.3 Data Security and Confidentiality 86
22.2.4 The SAS Data Codebook 86
22.2.5 Data/Resource Sharing Plan 86
22.3 Quality Control 87
22.3.1 Ensuring Data Accuracy and Completeness 87
22.3.2 Data Queries 88
22.3.3 Data Audits 88
22.3.4 Quality Control of Forms and the Data Entry System 88
22.3.5 Summary Reports 89
22.3.6 Site Visits 89
22.4 WUSTL Box 89
22.5 Randomization 89
Section 23: Participant Rights and Confidentiality 90
23.1 Institutional Review Board (IRB) 90
23.2 Informed Consent Forms (ICF) 90
23.2.1 Disposition of Informed Consent Forms 91
23.2.2 HIPAA Authorization 91
23.3 Participant Confidentiality 91
Section 24: Research Publication Policy 93

Section 25: Ancillary Studies Policies ... 94

25.1 Concurrent Studies ... 94

Section 26: Skeletal Muscle Mass using Creatine Dilution Method .. 95

26.1 General Information ... 95

26.2 Background and Significance ... 95

26.3 Study Objectives ... 96

26.3.1 Specific Aim 1 ... 96

26.3.2 Specific Aim 2 ... 96

26.3.3 Specific Aim 3 ... 96

26.3.4 Specific Aim 4 ... 96

26.4 Participants ... 96

26.4.1 Inclusion Criteria ... 96

26.2.2 Exclusion Criteria ... 96

26.5 Study Design ... 97

26.6 Recruitment and Retention ... 97

26.6.1 Informed Consent ... 97

26.6.2 Enrollment ... 98

26.6.3 Study Withdrawal ... 98

26.6.4 Study Discontinuation ... 98

26.6.5 Replacement of Subjects ... 98

26.7 Study Measures – University of California-Berkeley ... 98

26.8 Safety ... 98

26.8.1 Risks ... 98

26.8.2 Adverse Event Reporting ... 99

26.8.3 Site Monitoring ... 99

26.9 Statistical Analysis ... 99

26.9.1 Approach for Specific Aim 1 ... 99

26.9.2 Approach for Specific Aim 2 ... 99

26.9.3 Approach for Specific Aim 3 ... 99

26.9.4 Approach for Specific Aim 4 ... 99

Section 27: References ... 101

Acronyms Dictionary

1-RM	One Repetition Maximum
6MWD	Six Minute Walk Distance
ADLs	Activities of Daily Living
AE	Adverse Event
aLBM	Appendicular Lean Body Mass
ASC	Ancillary Studies Committee
BMD	Bone Mineral Density
BRS	Brief Resilience Scale
CCC	Clinical Coordinating Center
cPI	Contact Principal Investigator
CRF	Case Report Form
CSA	Cross-Sectional Area
DCC	Data Coordinating Center
DSMB	Data and Safety Monitoring Board
DXA	Dual-energy X-ray Absorptiometry
E	Estrogen
EC	Executive Committee
ECG	Electrocardiogram
EP	Exercise Physiologist
ES	Endocrine Society
EUC	Enhanced Usual Care Group
EX	Exercise intervention treatment group with placebo gel
EX+T	Exercise intervention treatment group with testosterone gel
FDA	US Food and Drug Administration
FFM	Fat-Free Mass
FSQ	Functional Status Questionnaire
GDS-SF	Geriatric Depression Scale – Short Form
HRPO	Human Research Protection Office
HRQ	Hip Rating Questionnaire
HRT	Hormone Replacement Therapy
HSL	Hebrew SeniorLife
IADLs	Instrumental Activities of Daily Living
IDS	Investigational Drug Service
ICF	Informed Consent Form
IM	Intervention Monitor
IMAGE	Investigations in Metabolism, Aging, Gender and Exercise (University of CO)
ISM	Internal Safety Monitor
LBM	Lean Body Mass
MOP	Manual of Procedures
mPPT	modified Physical Performance Test
NIA	National Institute on Aging
OARS	Older Americans Resources and Services Questionnaire
OHRP	Office for Human Research Protections
PC	Publications Committee
PCP	Primary Care Provider
PD	Program Director

PHI	Protected Health Information
PI	Principal Investigator
Pitt	University of Pittsburgh Medical Center
PK	Pharmacokinetic
PRT	Progressive Resistance Training
PT	Physical Therapist
QC	Quality Control
QOL	Quality of Life
RAE	Reportable Adverse Event
RCT	Randomized Controlled Trial
ROM	Range of Motion
SAE	Serious Adverse Event
SAQ-7	Seattle Angina Quesitonnaire-7
SC	Steering Committee
sIRB	Single Institutional Review Board
SPPB	Short Physical Performance Battery
STEP-HI	Starting a Testosterone and Exercise Program after Hip Injury
T	Testosterone
UCD	University of Colorado - Denver
UConn	University of Connecticut Health
UMJH	University of Maryland/Johns Hopkins
UofU	University of Utah
UP	Unanticipated Problem
UTMB	University of Texas Medical Branch at Galveston
WU	Washington University in St. Louis

Section 1: Executive Summary

Hip fractures are common in older women and can have a devastating impact on their ability to remain independent. A significant functional decline following a hip fracture has been documented even among individuals who were functioning at high levels before the event. Such individuals may require continued supportive services and are at high risk for recurrent falls and institutionalization. High-risk patients include those with deficits in skeletal muscle strength during the post-fracture period. Age-associated androgen deficiency contributes to deficits in muscle mass, strength and power that are common in older women before a hip fracture, and are exacerbated thereafter. A pilot study of testosterone (T) therapy in elderly female hip fracture patients demonstrated the feasibility of T therapy in this population, and showed gains in lean body mass and muscle strength in response to the active drug, compared to placebo. It is unclear whether these improvements can translate into sustained improvements in physical function, or whether combining T therapy with exercise can augment the effects of both treatments. There is also very little information available about the effects of testosterone therapy in older women with sarcopenia.

Study Title
Combining Testosterone Therapy and Exercise to Improve Function Post Hip Fracture

Study Acronym - STEP-HI Project
Starting a **T**estosterone and **E**xercise **P**rogram after **H**ip **I**njury

Objectives
The primary goal of this study is to establish that improvements from baseline to post-intervention (6-month) on the Six Minute Walking Distance (6MWD) are significantly greater for the combination of testosterone therapy and exercise training relative to a control condition or exercise training alone. In addition, a number of secondary outcomes will be evaluated, including 1-RM strength, body composition and bone mineral density of the non-fractured hip, objective physical performance tests, and self-reported performance of activities of daily living and quality of life.

Design and Outcomes
A randomized, double-blind, placebo controlled, parallel group study in older adult female hip fracture patients will be conducted at multiple clinical sites. All participants will complete a 6-month treatment period. Participants will be randomly assigned to one of ***three study groups***:

1. **Enhanced Usual Care** (Home exercise + Health Education) ***(EUC)***
2. **Exercise** (Placebo gel + Supervised Exercise Training) ***(EX)***
3. **Combined** (Testosterone gel + Supervised Exercise training) ***(EX+T)***

Females age 65 and older with a recent hip fracture at the clinical sites will be evaluated for eligibility between 6-24 weeks following surgery. After consent to participate, eligible participants will undergo a comprehensive screening assessment. Those who complete the screening assessment and meet all eligibility criteria will be randomized to one of the three study groups after completing the baseline assessments. Randomization of between 120 and 168 participants with recent surgical repair of a hip fracture will take place within 24 weeks following surgery.

Primary and secondary study outcomes will be measured at baseline, 3 months and 6 months post-randomization. Safety measures and adherence measures will be collected monthly and a brief questionnaire may be administered at 9 months post randomization.

Section 2: Study PIs and Participating Clinical Sites

2.1 STEP-HI PIs

Contact PI, Program Director (**cPI/PD**)
Steering Committee Chair and Clinical Coordinating Center Director
Ellen Binder, MD
Washington University in St. Louis
660 South Euclid, Campus Box 8303
St. Louis, MO 63110
Phone: (314) 286-2707
Fax: (314) 286-2701
ebinder@wustl.edu

Study PI and Data Coordinating Center Director
Ken Schechtman, PhD
Washington University in St. Louis
660 South Euclid, Campus Box 8067
St. Louis, MO 63110
Phone: (314) 362-3606
kschechtman@wustl.edu

Study PI
Jay S. Magaziner, PhD, MSHyg
University of Maryland
660 West Redwood St., Suite, 200 Howard Hall
Baltimore, MD 21201
Phone: (410) 706-3553
Fax: (401) 706-4433
jmagazin@epi.umaryland.edu

2.2 Participating Clinical Sites

Boston (1)
Hebrew SeniorLife (HSL)/Beth Israel (BI)/Harvard Medical School

Douglas P. Kiel, MD, MPH
Clinical Site PI
1200 Centre Street
Boston, MA 02131
Phone: (617) 971-5373
Fax: (617) 971-5339
kiel@hsl.harvard.edu

Sarah Berry, MD, MPH
Clinical Site Co-Investigator
1200 Centre Street
Boston, MA 02131
Phone: (617) 971-5383
Sarahberry@hsl.harvard.edu

St. Louis (2)
Washington University in St. Louis (WU)
Ellen Binder, MD
Clinical Site PI
660 South Euclid, Campus Box 8303
St. Louis, MO 63110
Phone: (314) 286-2707
Fax: (314) 286-2701
ebinder@wustl.edu

Denver (3)
University of Colorado (UCD)
Kerry Hildreth, MD
Clinical Site PI
Campus Box: B-179
12631 East 17th Ave.
Aurora, CO 80045
Phone: (303) 724-1919
Fax: (303) 724-1918
Kerry.Hildreth@ucdenver.edu

Connecticut (4)
University of Connecticut (UConn Health)
George Kuchel, MD, CM, FRCP, AGSF
Clinical Site PI
263 Farmington Avenue
Farmington, CT 06030
Phone: (860) 679-6796
Fax: (860) 679-1307
kuchel@uchc.edu

Richard Fortinsky, PhD
Clinical Site Co-Investigator
263 Farmington Avenue
Farmington, CT 06030
Phone: (860) 679-8069
Fax: (860) 679-1307
fortinsky@uchc.edu

Baltimore (5) (UMJH)
University of Maryland Baltimore
Denise Orwig, PhD
Clinical Site PI
660 West Redwood Street, Suite 200
Baltimore, MD 21201
Phone: (410) 706-2406
Fax: (410) 706-4433
dorwig@som.umaryland.edu

Johns Hopkins University
Adrian Dobs, MD, MHS
Clinical Site Co-Investigator
1830 Monument Street, Suite 328
Baltimore, MD 21287
Phone: (443) 287-4000
Fax: (410) 955-8172
adobs@jhu.edu

Galveston (6)
University of Texas Medical Branch at Galveston (UTMB)
Elena Volpi, MD, PhD
Clinical Site PI
301 University Blvd
Rebecca Sealy Hospital - 6.610
Galveston, TX 77555
Phone: (409) 772-1977
Fax: (409) 772-8931
evolpi@UTMB.EDU

Salt Lake City (8)
University of Utah Health (UofU)
Robin Marcus, PT, PhD
Clinical Site PI
Department of Physical Therapy and Athletic Training
520 Wakara Way, Rm 125
Salt Lake City, UT 84108
Phone: (801) 581-4813
Fax: (801) 585-5629
robin.marcus@hsc.utah.edu

Pittsburgh (9)
University of Pittsburgh (Pitt)/ University of Pittsburgh Medical Center (UPMC)
Christine McDonough, PT, PhD
Clinical Site PI Bridgeside Point 1
100 Technology Dr.
Pittsburgh, PA 15219
Phone: (412) 383-4603
cmm295@pitt.edu

Section 3: Study Organization & Administration

This study is a multi-site, randomized, double-blinded, placebo controlled, parallel group study with a Clinical Coordinating Center (**CCC**) and Data Coordinating Center (**DCC**) at Washington University in St. Louis (WU), which will lead the clinical sites. In addition to these units, the project will have a Steering Committee (**SC**) that will provide the expertise needed to conduct this trial. A project Organization Chart is provided below. Each clinical site will be affiliated with a university and will be recruiting females aged 65 and older who have experienced a recent hip fracture repair. Most of the key personnel have collaborated on the design of this study; several will have multiple roles in its execution, based on individual areas of expertise.

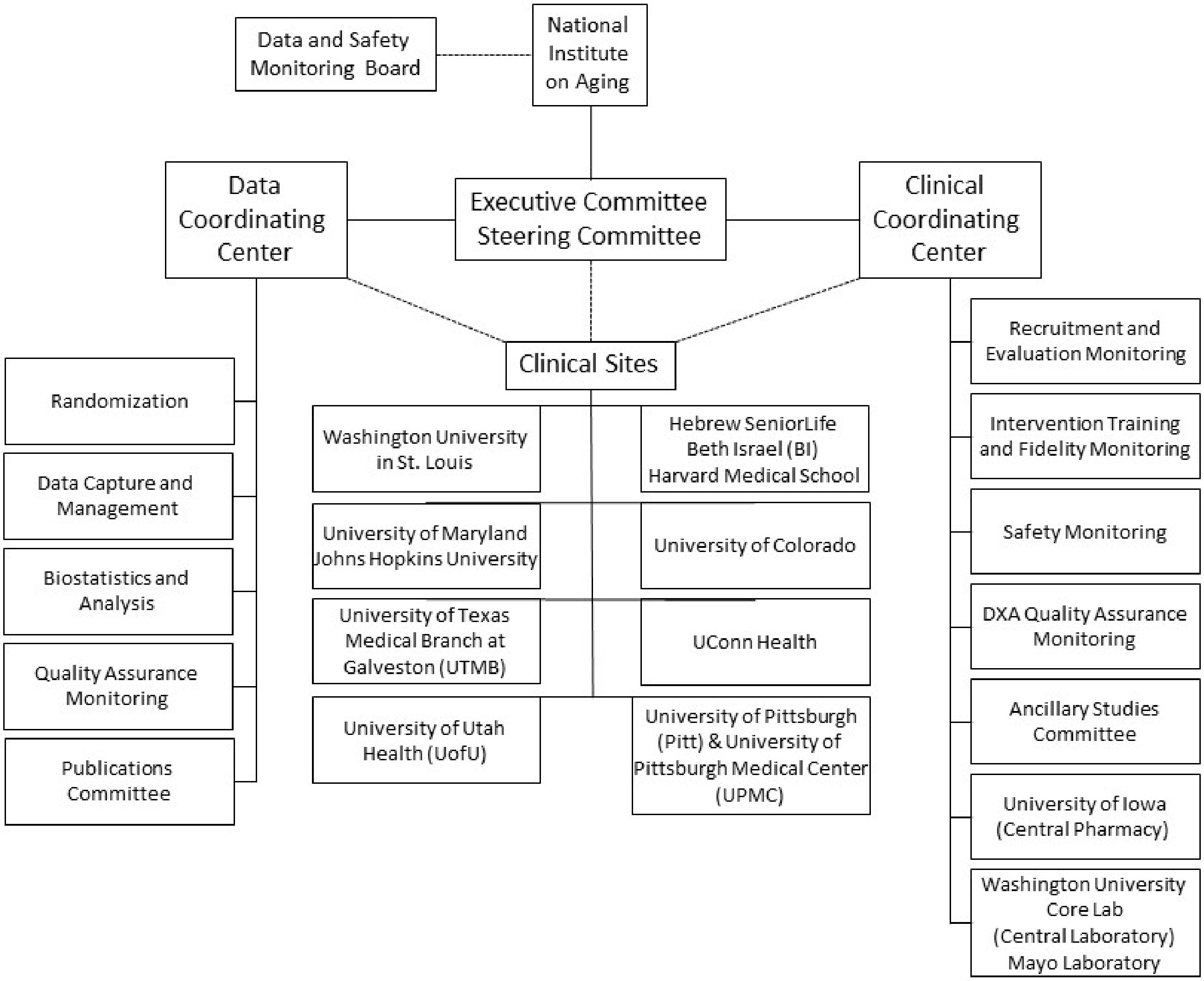

3.1 Study Chair

Dr. Ellen Binder, contact principal investigator (**cPI**) and Project Director (**PD**) of the grant awarded by the National Institute on Aging (**NIA**) will serve as the study chair. Responsibilities of the study chair will include:

- Providing overall organization and scientific direction of the trial
- Serving as chair of the SC
- Administering logistics for the Data and Safety Monitoring Board (**DSMB**) in consultation with the NIA program official
- Working with investigators and staff in the CCC, DCC, and clinical sites to maximize collaboration
- Providing updates on progress to the NIA
- Participating or supervising site visits to clinical sites to assess quality and assist with problems
- Defining analyses of study data
- Overseeing manuscript preparation

In the unlikely event the Study Chair becomes unable to serve, another Study PI will serve as the Study Chair.

3.2 National Institute on Aging (NIA)

This is an investigator-initiated project and funding is provided by an R01 grant. The funding agency is the NIA. The NIA will appoint members of the DSMB, who will review study data and safety presented by the study team and report to the NIA program official. The NIA program official will then report the outcome of the DSMB review to the NIA director following established internal procedures. The PI will also report study progress to the NIA on an annual basis unless asked to report at a different interval. According to PA-10-067, a Non-Competing Continuation Grant Progress Report (PHS 2590) will be completed by the PI annually and financial statements will be provided as required in the *NIH Grants Policy Statement*. A final progress report, invention statement, and Financial Status Report will be submitted by the study chair when the award is relinquished or when it is terminated.

3.3 Data and Safety Monitoring Board (DSMB)

Members of the DSMB will be appointed by and report to the NIA. They will monitor accruing data in order to confirm that the participants in the trial are being cared for safely. Responsibilities of the DSMB will include:

- Review of the research protocol, informed consent documents and plans for data and safety monitoring
- Advise the NIA on the readiness of the study investigators and staff to initiate recruitment
- Evaluate the progress of the trial, including periodic assessments of data quality and timeliness, recruitment, accrual and retention, participant risk versus benefit, performance of the trial sites, and other factors that can affect study outcome
- Consider factors external to the study when relevant information becomes available, such as scientific or therapeutic developments that may have an impact on the safety of the participants or the ethics of the trial

- Review, approve, and monitor ancillary studies
- Review study performance, make recommendations and assist in the resolution of problems reported by the PI
- Protect the safety of the study participants
- Report to the NIA on the safety and progress of the trial
- Make recommendations to the NIA and the cPI concerning continuation, termination or other modifications of the trial based on the observed beneficial or adverse effects of the treatment under study
- If appropriate, review the results of interim analyses in accordance with stopping guidelines, which are clearly defined in advance of data analysis and have the approval of the DSMB
- Ensure the confidentiality of the study data and the results of monitoring
- Assist the NIA by commenting on any problems with study conduct, enrollment, sample size and/or data collection

The DSMB will discharge itself from its duties when the study is complete. Study completion will be considered in consultation with the study chair and NIA after no further outcome data are being collected in the main study and the main paper reporting on the primary outcome has been published.

3.4 Executive Committee (EC)

The EC consists of the three study PIs (Contact PI, DCC Director, and Study Co-Investigator). Responsibilities will include:

- Supervising the overall conduct of the trial
- Serving as primary liaison body to the NIA, regulatory agencies, and the study DSMB
- Chairing Steering Committee Meetings

The Study Chair will chair Steering Committee Meetings. When the Study Chair is unavailable one of the other two Study PIs will chair the meeting.

3.5 Steering Committee (SC)

The SC will be charged with the overall governance of the study. Responsibilities will include:

- Approving the final protocol and Manual of Procedures
- Generating and approving study policies
- Considering modifications of the protocol and study operations
- Providing oversight of the CCC and DCC
- Problem-solving issues brought forward by the CCC, DCC, Site PIs, and coordinators
- Reviewing issues related to protocol deviations and making final determinations regarding continued participation
- Monitoring study performance
- Appointing and chairing sub-committees
- Implementing recommendations from the DSMB

Voting members of the SC will include the Contact PI, one member from each clinical site (Clinical Site PI or designee), and the other 2 Study PIs. If the contact PI also serves as the

Site PI then only one vote will be allowed; the 2 Study Co-Investigators will be allowed one combined vote (to ensure an uneven number of voting members). All major scientific decisions will be determined by a simple majority of SC voting members. The SC will meet at least monthly throughout the study.

3.6 Clinical Coordinating Center (CCC)

The CCC will be primarily responsible for managing study operations and ensuring adherence to the study protocol. Responsibilities will include:

- Finalizing the study protocol, Manual of Procedures (MOP) and any subsequent amendments
- Submitting study protocol, consent form, and any subsequent amendments to the WU Single Institutional Review Board (**sIRB**)
- Tracking protocol amendments and ensuring their implementation
- Procuring standardized equipment and materials for the clinical sites
- Providing standardized study materials to clinical sites
- Tracking protocol deviations and developing Corrective Action Plans
- Tracking the implementation of the Corrective Action Plans
- Staffing of an unblinded physician to monitor participant testosterone and CBC levels and recommend adjustments as necessary
- Organizing and providing staff support for meetings of committees
- Recording minutes from Steering Committee meetings, and sub-committee meetings, as indicated
- Organizing training sessions of site coordinators, evaluators, dieticians, and PTs/exercise interventionists
- Providing training related to clinical operation of study
- Producing documents and forms in collaboration with the DCC
- Reviewing reportable adverse events (**RAEs**) and submitting RAEs and Serious Adverse Events (**SAEs**) to the sIRB, DSMB, FDA, and NIA as per the protocol
- Developing monitoring and quality assurance plans
- Performance of monitoring and quality assurance visits to the clinical sites
- Monitor sites for human subjects training and certification records, including CITI training and GCP certification
- Interfacing with the Investigational Drug Service (IDS) to ensure proper distribution of study drug and placebo to each clinical site
- Interfacing with the Central Lab to ensure proper distribution of lab kits and facilitating the reporting of results to the appropriate individuals
- Interfacing with the Central DXA Imaging Center at UCD to ensure timely review of DXA scans and transfer of data to the DCC

3.6.1 CCC Oversight and Monitoring Responsibilities

The CCC will refine and optimize procedures and strategies for recruitment and retention of study participants and for maintaining adherence to the protocol. The CCC will oversee recruitment progress at all sites, intervene in cases of under-recruitment, and report recruitment progress to the SC. The CCC will oversee retention efforts and investigate and intervene when a site is having retention problems. The CCC will utilize reports from the study database which will allow monitoring of recruitment, retention, and data collection by clinical site. Another

responsibility of the CCC is ensuring that protocols are followed for data collection procedures and that each clinical site establishes standard operating procedures and maintains a current version throughout the study. The CCC will also monitor data collection for the STEP-HI study and approved ancillary studies.

3.6.2 CCC Intervention Training and Fidelity Monitoring

The CCC will finalize and refine the study Manual of Procedures (MOP) and work closely with the clinical sites to implement related training to provide the intervention while following Quality Control (**QC**) procedures. The CCC will be responsible for the procedures for training, and certifying all PTs and exercise interventionists involved in the intervention protocols, documenting any changes to procedures in the MOP, and distributing updates to the clinical sites.

3.6.3 Ancillary Studies Committee

The Ancillary Studies Committee (**ASC**) will review proposals for ancillary studies and provide a recommendation for/against approval to the SC. Members of the ASC will be appointed by the Study Chair. The ASC Chair will be responsible for distributing proposals and related materials to committee members, organizing meetings and recording minutes, and providing reports to the SC. See Section 25 for Ancillary Studies Policies.

3.7 Data Coordinating Center (DCC)

The DCC's primary responsibility will be to manage data operations, monitor adherence to the study protocol and implement the data analysis plan. Responsibilities will include:

- Developing, implementing, and monitoring the data management and randomization plans, and, in collaboration with the CCC, the data safety and monitoring plan.
- Overseeing training of clinical site staff with regard to data entry, data correction, and randomization procedures
- Assisting CCC on development of data collection form templates and overseeing the implementation of paper-based and web-based forms
- Monitoring completion and timeliness of case report form submissions and query responses
- Developing and implementing data edit specifications
- Monitoring distribution of data queries to clinical sites
- Monitoring sites' responsiveness to queries
- Participating in quality assurance visits to the clinical sites
- In collaboration with the CCC, preparing reports on study progress and study results for the DSMB (twice per year and upon request), the NIA (annually and upon request), sIRB (annually and upon request), and the SC (upon request)
- Preparing data files to be shared with any ancillary studies that are approved by the SC
- Developing and maintaining a file share site in WUSTL Box to ensure up-to-date study documents (e.g., data collection forms, manuals, protocol, study personnel contact information)
- In collaboration with the Publications Committee, Steering Committee, and CCC, preparing final data files and performing data analyses for publications and presentations

3.7.1 Publications Committee

The Publications Committee (**PC**) will review proposals for abstracts and manuscripts and provide a recommendation for/against approval to the Steering Committee. Members of the PC will be appointed by the Study Chair. The DCC Director will chair the PC, and will be responsible for distributing proposals and related materials to PC committee members, organizing meetings and recording minutes, and providing reports to the SC.

3.8 Single Institutional Review Board (sIRB)

A sIRB will provide oversight of this study as related to the protection of human subjects and compliance with related federal regulations. The Washington University Human Research Protection Office (**HRPO**) will serve as the sIRB. For more information see Section 23.

3.9 Central Pharmacy Services

The University of Iowa Pharmaceuticals will be responsible for the purchase, relabeling, and distribution of the active investigational drug. The University of Iowa Pharmaceuticals or a similar pharmacy contracted by the CCC will be responsible for packaging, labeling, and distributing the placebo gel to the clinical sites. Each clinical site will be responsible for maintaining thorough records on the STEP-HI Investigational drug accountability log showing receipt, distribution, and destruction (in the case of expired drug) for investigational drug at their site, through an Investigational Pharmacy or Drug Service as directed by the standard operating procedures at their institution.

3.10 Centralization of Laboratory Testing

Washington University Core Laboratory will provide centralized laboratory services for all clinical sites. The Central Lab will provide pre-assembled kits for blood draws based on assessment time point and supply shipping kits for return to the appropriate processing lab. The Central Lab will provide training and a MOP for all procedures related to laboratory testing. The central lab will provide results in a timely fashion to the DCC, individual site PIs, or Unblinded CCC physician as indicated.

3.11 Central DXA Imaging Center

The University of Colorado Denver Investigations in Metabolism, Aging, Gender and Exercise (IMAGE) center will be responsible for managing the dual energy X-ray absorptiometry (DXA) imaging component of the STEP-HI Project. They will be responsible for the DXA Acquisition Manual which will be included in the Manual of Procedures, oversee certification of DXA technicians, perform image review, analysis of DXA scans obtained using Hologic machines and software, and assist CCC staff with quality control for all STEP-HI clinical sites.

3.12 Clinical Sites

The study has collaborating clinical sites each with a clinical site PI and coordinator. The clinical sites are Washington University in St. Louis (**WU**), Baltimore-University of Maryland/Johns Hopkins (**UMJH**), University of Connecticut Health Center (**UConn**), Hebrew SeniorLife (**HSL**), University of Colorado - Denver (**UCD**), and University of Texas Medical Branch at Galveston (**UTMB**), University of Utah Health (**UofU**), and University of Pittsburg Medical Center (**UPMC**).

Responsibilities of the clinical site PI (with assistance from the coordinator) will include:

- Maintaining cooperation of study hospitals and ensuring that medical staff involved with the care of hip fracture patients are well informed about the trial
- Recruiting study participants according to the study protocol
- Ensuring retention and adherence of study participants
- Performing all study-related assessments (including complete tracking of outcomes during follow-up)
- Overseeing completion of data collection forms, enrolling participants using the automated randomization system, and entering data and processing data edit queries
- Training (in collaboration with the CCC) and supervising staff at the clinical site; assigning tasks to data collectors, PTs, and dieticians; and providing day-to-day supervision of their work
- Protecting participant safety and verifying that informed consent procedures are followed according to Good Clinical Practice guidelines
- Providing oversight to safety monitoring at their individual clinical site
- Properly maintaining all clinical site study materials and records
- Reporting all Reportable Adverse Events (RAEs) and protocol deviations
- Participating in the study sub-committees and manuscript preparation

Section 4: Study Objectives

4.1 Primary Objective

Primary Aim

<u>Specific Aim 1</u>: To determine whether improvements in functional status, as measured by the *Six Minute Walk Distance* (**6MWD**) from baseline to post-intervention (month 6), are significantly greater for **EX+T** relative to **EX** alone, and relative to **EUC**.

Hypothesis 1: Compared to **EX** and **EUC,** the **EX+T** group will have significantly greater improvements in 6MWD.

4.2 Secondary Objectives

<u>Specific Aim 2</u>: To determine whether improvements from baseline to post-intervention (month 6), are significantly greater for **EX+T** relative to **EX** alone, and relative to **EUC,** for the following outcome measures:

a) Whole body and appendicular lean body mass (LBM), measured using dual-energy x-ray absorptiometry (DXA);

b) Leg press strength measured by the 1-repetition maximum test (1-RM);

c) Functional performance measured by objective physical performance tests (modified Physical Performance Test Score (mPPT), Short Physical Performance Battery (SPPB); performance of Activities of Daily Living (ADLs) measured by self-report;

d) Quality of life measured by self-report (Hip Rating Questionnaire, PROMIS® - Global Health);

e) Bone mineral density (BMD) of the non-fractured proximal femur, measured by DXA.

Hypothesis 2 : Compared to **EX** and **EUC,** the **EX+T** group will have greater improvements in LBM, 1-RM strength, objective physical performance measures, self-reported ADLs, self-reported quality of life, and BMD of the non-fractured proximal femur.

4.3 Exploratory Objectives

<u>Specific Aim 3</u>: To obtain preliminary data regarding the sustainability of the effects of the **EX+T** and the **EX** interventions at 3 months after discontinuation of the active interventions (i.e. month 9 after randomization) on self-reported measures of ADL function and quality of life.

Section 5: Background

Hip fractures represent a major public health problem of increasing magnitude. Annual incidence in the United States is approximately 98 fractures per 100,000 population, and an estimated 300,000 Americans aged 65 years and over sustain a hip fracture each year[1, 2]. The incidence of hip fractures increases exponentially with age, so that 90% of such fractures occur after age 70[3] years. The majority of hip fractures occur in females over 60 years of age, and white females aged 85 years and older are the population at highest risk[4]. As this is one of the fastest growing segments of our population, the burden of care for hip fracture patients is anticipated to rise dramatically.

5.1 Consequences of Hip Fracture

Although advances in surgical techniques have reduced mortality after hip fracture repair, many patients remain unable to achieve full functional recovery. Studies have documented that between 22% and 76% of hip fracture patients do not recover to their pre-fracture ambulatory or functional status within 6 to 12 months of the fracture event[5-8]. It is notable that although poor pre-fracture functional status increases the risk of poor recovery, many previously high functioning patients have significant mobility and ADL deficits after a hip fracture. Up to 60% of previously high functioning patients required assistance in ADLs, and 83% required the use of an assistive device at 6 months post-fracture[9]. Magaziner et al. found that 25-40%[8, 10] of patients show a decline in walking ability, and 20% demonstrate a decline in instrumental activities of daily living (IADLs=shopping, laundry, etc.) between 6 and 12 months post-fracture. Patients who experienced such declines are more likely to experience re-hospitalization or a fall. Thus, despite standard rehabilitation techniques, many previously independent older patients require ongoing assistance with ADLs and IADLs between 6 and 12 months post-fracture. Interventions designed to reduce physical impairments post-fracture may thus be beneficial for long-term recovery, functional independence, and as a pathway to reduce health care costs.

5.2 Rehabilitation and Exercise Strategies after Hip Fracture

Information about the relationship between functional outcomes and the amount or intensity of rehabilitation services is limited, despite a growing body of literature in this area. A Cochrane Review of multidisciplinary rehabilitation for older people with hip fracture included 13 trials and could only conclude that multidisciplinary team management is not harmful and may improve functional recovery[11]. It did not address questions related to the intensity or duration of therapy services. A second Cochrane Review of interventions for improving mobility after hip fracture included 19 trials of “mobilization strategies”[12]. Twelve trials evaluated interventions soon after surgical repair, and found conflicting results regarding the efficacy of weight-bearing, strengthening, or intensive PT interventions on mobility. Of seven trials of interventions started after hospital discharge, including one by Binder, et al. [13], three included intensive PT or strengthening programs, and showed improvements in mobility outcomes. The authors concluded that there is still insufficient evidence to establish the best strategies to enhance mobility after hip fracture. There was a suggestion that intensive strengthening programs started after hospital discharge improve short- and long-term mobility outcomes, but more evidence is needed to support this conclusion. The exercise intervention described in this proposal is based on previous work by Binder, et al. [13], showing that intensive multi-component exercise is safe and effective for hip fracture patients with physical frailty and impaired mobility. A recently published trial of a home-based intervention[14] that was considerably less intensive,

demonstrated very modest improvements in physical function that were comparable to the improvements observed in the Control group in Binder's trial.

5.3 Decrements in Muscle Mass and Strength after Hip Fracture

Factors associated with poor post-fracture outcomes also may contribute to the etiology of the fracture itself and place the patient at very high risk for recurrent falls and fractures. Decrements in neuromuscular function contribute to the risk of hip fracture. Two studies have documented an association between reduced muscle strength and increased fracture risk[15, 16]. Fox, et al. [17] documented a 6% decline in lean body mass at 12 months post-fracture in a sample of elderly female hip fracture patients. Older hip fracture patients are at greater risk for decrements in muscle strength pre- and post-fracture because of age-associated changes in skeletal muscle. Decrements in muscle mass and muscle power are associated with functional impairments[18-21] and disability[22-24] in the elderly.

In a previous RCT of intensive exercise in frail hip fracture patients by Binder et al., clinically significant improvements in measures of physical performance, including muscle strength, were observed. However, significant group differences in the changes in fat-free mass (FFM) over the 6-month treatment period were not observed[13]. This raises a question as to whether anabolic therapies aimed at increasing FFM and muscle strength, when combined with intensive exercise, can enhance the anabolic effects of exercise and achieve greater improvements in physical function and health outcomes than exercise alone. This study aims to address this question.

5.4 Testosterone Deficiency in the Elderly and in Hip Fracture Patients

The major biologically active circulating androgen in males and females is testosterone (T). Evidence from a number of studies in men strongly suggests that total, free, and bioavailable T levels in serum decrease with advancing age [25-28]. T deficiency is associated with decreases in lean body mass[29] and bone mass[30] in young, hypogonadal men. In aging men, a decline in testosterone and an increase in pro-inflammatory markers such as IL-6 and TNFα are associated with decreased muscle and bone mass[31], and may contribute to physical frailty[32]. There is a paucity of information regarding the clinical significance of low testosterone T levels in elderly women[33]. After menopause, ovarian production of androgens (including T) persists, but declines[34]. Plasma T levels have been documented to decrease by approximately 50% following ovariectomy [35]. Results from cross-sectional studies of age-related changes in T levels in women are variable. The majority of studies suggest a gradual decline in T levels between age ~20 years and menopause, with levels in post-menopausal and elderly women 30% to 50% below that of pre-menopausal women[34, 36-40].

In women, low serum T levels have been associated with lower BMD [41, 42] and hip fracture [43-45]. In the Rancho Bernardo Study, bioavailable T levels in women were positively correlated with loss of vertebral height over 16 years [46]. In another cross-sectional study of men and women (aged 20-90 years), Khosla et al.[37] found that bioavailable T and estrogen (E) levels correlated with BMD of the spine and femur, though multivariate analysis showed that E was the only independent predictor of BMD. However, in both men and postmenopausal women E is produced in large part by conversion of T. Therefore, low T levels may contribute indirectly to the effects of low E levels on BMD. Dubin, et al. [47] found that women with hip fractures had lower total T levels at the time of fracture than healthy controls, though the difference did not

reach statistical significance. Women with a trochanteric fracture also had a significant decline in T, but not E or other androgen levels, over the 12 months following the fracture.

Age-associated decrements in skeletal muscle mass are well documented in women, although the underlying mechanisms are not well understood. Women lose fat-free mass with the onset of menopause[48, 49]. In 43-73 year old women, Hakkinen and Pakarinen [50] found associations between serum total and bioavailable T level, and muscle cross-sectional area (CSA) ($r=0.46$, $p<0.05$), and maximal muscle force production ($r=0.62$, $p<0.01$). Gower et al.[51] also found a significant correlation between free T levels, and total lean mass ($r=0.24$, $P<0.5$) and leg lean mass ($r=0.31$, $p<0.01$) in postmenopausal women ($n=70$). In 59-78 year old women using HRT for 2 years, Iannuzzi-Sucich et al.[52] found that total T, but not E level, was an independent predictor of DXA-derived appendicular skeletal muscle mass.

These studies suggest that low bioavailable T levels in women may: 1) be a risk factor for hip fracture; 2) contribute to age-associated declines in skeletal muscle mass and strength, and bone mass and; 3) contribute to declines in lean body and bone mass during the year following a hip fracture. However, data regarding the relationship between serum T-levels and age-associated declines in muscle mass and strength are very limited in women, and there is a need for more clinical studies to address this issue[31, 53].

5.5 Rationale for the Interventions

There have been very few studies of anabolic steroids in women focused on skeletal muscle strength and physical function, and most have been conducted with younger, healthier women. Hip fracture patients have a high prevalence of sarcopenia, which is an important risk factor for persistent mobility and ADL impairment. Treatment of elderly female hip fracture patients with short-term T therapy represents a novel treatment modality. In light of the modest effects of standard care exercise observed in hip fracture patients, the addition of an anabolic agent to intensive exercise has great potential to yield significant improvements in post-fracture functioning. Combining short-term T therapy and exercise, if demonstrated to be safe and efficacious, could lead to a major change in clinical practice for female hip fracture patients, and could have a huge impact on the approach to clinical care for this patient population, and other female patient populations after periods of hospitalization and immobility.

5.5.1 Androgen Replacement

Studies of the effects of androgen therapy have been performed primarily in hypogonadal and healthy elderly men; patients with chronic diseases associated with muscle wasting, such as AIDS, COPD, chronic renal failure; surgical wounds; and in women with postmenopausal osteoporosis. Several studies have demonstrated that T replacement increases fat-free mass and decreases fat mass in young eugonadal[54] and hypogonadal men[55-57], elderly hypogonadal men[58], and in middle-aged and elderly men with low T levels[27, 59, 60]. T replacement in hypogonadal men also increases maximum voluntary muscle strength [27, 59]. Studies in elderly men with low T levels are inconsistent with respect to effects on muscle strength[61, 62]. Studies that did not show positive effects on muscle strength enrolled men that were not uniformly hypogonadal, were healthier, and/or were more physically fit[27] or used dynamometry to assess muscle strength[58]. In a placebo-controlled study, Bakhshi et al.[63] observed improvements in grip strength and measures of ADL function in response to T therapy in frail elderly men admitted to a VA Geriatric Evaluation unit (GEM) for rehabilitation. Another small, placebo-

controlled trial of T therapy prior to elective knee replacement in older men showed significant improvements in transfer ability, and trends for decreased hospital LOS and improved mobility, in the T treatment group[64]. Oxandrolone, a synthetic analog of testosterone, has been shown to increase muscle protein synthesis rate in young men[65, 66] and has also been shown to increase body weight and lean body mass (LBM) in young men with HIV[67], surgical patients with non-healing wounds[68], elderly men and women with severe burns[69], and male and female COPD patients with weight loss[70]. Two recent RCTs of T therapy in older men with physical frailty[71, 72] showed significant increases in LBM, muscle strength and physical function, although a study by Basaria et al.[72] reported a higher incidence of adverse cardiac and respiratory events in the T group than placebo, raising some concerns about the safety of T therapy in frailer populations.

Studies of the effects of androgen therapy on LBM in women are both limited and inconsistent[31, 33]. Lovejoy[73] conducted a 9-month RCT comparing nandrolone, spironolactone, and placebo in 30 healthy, obese, postmenopausal women; they observed significant group differences in the changes in serum T levels, with ~2 kg increase in LBM in the nandrolone-treated group. Davis[74] conducted a 2-year RCT of 50 mg of estradiol (E) vs. or E+T implants (50mg), finding an increase total T levels to the upper range of normal for premenopausal women (Total T=2.3 nM/L). The E+T group also had increased fat-free mass (FFM) (~3 kg, vs. a decline in the E group) over 2 yrs. Dobs[75] conducted a 16-wk RCT of oral E (1.25 mg) vs. E+T (2.5 mg methyl-testosterone) in post-menopausal women, observing significant increases in FFM and lower extremity strength only in the E+T group. Recently Huang[76] conducted a 6-month RCT in 71 healthy women (mean age ~54 yrs.) with low serum T levels after hysterectomy +/- oophorectomy who received E+placebo vs. one of 5 doses of intramuscular T. Dose-dependent improvements were observed in FFM, chest-press power and loaded stair-climb power in the T-treated subjects. Women receiving the higher T doses achieved supraphysiologic serum T levels which were well tolerated during the 6-month study.

Miller[77] conducted a 12-week, RCT of 2 transdermal T doses in 54 women with AIDS wasting and low T levels. They found that while women on the physiologic T dose (total T levels ~100 ng/dl) had increased body weight, only those on the supraphysiologic dose (total T ~155 ng/dL) had increased FFM (+0.5 kg). Both T doses were well tolerated, with no differences in adverse events. Dolan[78] conducted a RCT of physiologic T replacement in 57 HIV-infected women with weight loss and low T levels. T therapy was well tolerated and resulted in significant increases in muscle mass, measured by urinary creatinine excretion, and several measures of muscle strength. Choi[79] conducted a RCT of physiologic T replacement in 52 HIV-infected women with weight loss, and observed no significant changes in FFM, leg strength or power, or quality of life.

Androgen or T therapy in younger women and HIV+ women can increase serum T levels and, in some studies, is associated with improvements in body composition and muscle strength. In these short-term studies, even supraphysiologic dose of T have been well tolerated. There are little or no data on changes in function, in older women or women post-hip fracture. This study will address these knowledge gaps.

5.5.2 Rationale for targeting female hip fracture patients

This study will focus on female hip fracture patients for several reasons. First, females comprise ~75% of elderly hip fracture patients, and therefore study findings will impact a large proportion of this patient population. Second, in our experience conducting a pilot study of testosterone therapy in older men with physical frailty, we found that a high proportion of male hip fracture patients had contraindications to testosterone therapy due to prostate disease or other medical issues. Third, because of concerns about potential sex differences in the response to testosterone therapy, it would be necessary to enroll a sufficient number of men to perform sub-group analyses, which would require a very large sample. Lastly, there are already more data available about testosterone therapy in older men with sarcopenia and mobility impairments.

5.5.3 Rationale for a 3-Group Design

We considered a 2x2 factorial design, which would allow us to investigate the effects of each intervention alone in comparison to placebo. The required sample size was ~400+ subjects, which would require more clinical sites and be more costly. Because hip fracture patients who undergo surgical repair all receive some form of exercise as part of their rehabilitation, we do not think that a testosterone alone arm has much “real world” applicability, and therefore planned a design that will allow us to evaluate the benefits of adding testosterone to an exercise program that has demonstrated benefits in the target population.

5.5.4 Rationale for a 6-month intervention period

Although a shorter intervention period has the advantages of lower costs and attrition rates, our previous studies of testosterone, and those of other anabolic agents conducted in older adults, suggest that a treatment duration of 6 months may be necessary to translate into clinically meaningful improvements in muscle mass, strength, and function.

5.5.5 Preliminary Data/Efficacy and Safety of Testosterone Replacement after Hip Fracture

Dr. Binder conducted a pilot study entitled *Testosterone therapy after hip fracture in elderly female hip fracture patients (**R21 AG023716 PI Binder**).* The study aims were to: 1) document the feasibility of recruiting frail elderly female hip fracture patients to participate in a 6-month RCT of topical testosterone therapy; 2) obtain preliminary information about the tolerability and safety of a supra-physiologic dosage of topical testosterone (T) therapy; 3) obtain preliminary information regarding the effects of 6 months of T therapy on a number of outcomes, including lean mass, muscle strength, physical functional capacity, and quality of life. Participants met inclusion criteria similar to those for this clinical trial. Women were randomly assigned to placebo or T therapy for 6 months, using a topical gel, at a supra-physiologic dose (target serum T level range 110-160 ng/dL). All women were prescribed a low-intensity flexibility home exercise program that was not intended to increase muscle strength, similar to standard of care upon discharge from a post hip fracture PT program. Adherence to therapy, based on monthly weights of the gel bottle, was 79% (83% placebo; 77% T gel). Only two women (1 T-gel, 1 placebo) dropped out; both determined to be unrelated to the study or medication.

Table 1. Baseline and Change in Outcomes after 6 months of Testosterone therapy

Variable	Time point	Placebo Group (n=5)	Testosterone Group (n=9)	p-value

Testosterone Level (ng/dL)	Baseline	20.2 ± 9.1	19.7 ± 11.3	
	Change	16.2 ± 10.3	145.2 ± 88.2	0.007
Lean Body Mass (kg)	Baseline	38.6 ± 2.2	38.0 ± 6.3	
	Change	-0.01 ± 0.9	1.6 ± 1.8	0.03
Knee Extension 1-RM (lbs.)	Baseline	63.1 ± 16.3	61.4 ± 22.0	
	Change	6.3 ± 9.2	12.8 ± 8.6	0.20
Hip Rating Questionnaire (HRQ) Score	Baseline	79 ± 13	77 ± 12	
	Change	0.8 ± 13	6 ±10	0.45

As shown in the table above, the target T level in the T-gel group was achieved. We observed a significant group difference in the changes in lean body mass, and trends toward greater improvements in leg strength and quality of life (HRQ score) in response to T therapy.

5.5.6 Safety Measures in Pilot Study

Despite careful monitoring for signs of hirsutism, endometrial hyperplasia, changes in hematocrit, serum LFTs and lipids, no differences were observed for any of the safety parameters or the frequency of reported symptoms. There was one report of increased arm hair growth (T-gel group) that resolved several months after discontinuation of the medication. Although the sample size was very small and insufficient to draw definitive conclusions about the safety of testosterone therapy in this population, it did provide valuable preliminary data on patient recruitment, effect size for outcome measures, and information that the proposed dose of topical T therapy is well tolerated.

5.5.7 Addressing Safety Concerns

The pilot study conducted at WU demonstrated the feasibility of administering topical testosterone gel to frail women with hip fractures, who tolerated the medication at the proposed dose well and demonstrated improvements in key outcome measures that will be used in the larger trial. These preliminary data were used to design this multicenter trial with a larger sample size.

In designing this larger study we have carefully considered safety concerns given recent studies that have raised questions about the safety of testosterone replacement in men(72), and the 2014 Endocrine Society (ES) Clinical Practice Guideline for Androgen Therapy in Women(80). A systematic review and meta-analysis of testosterone(81) and DHEA therapy in women performed for this guideline found a higher rate of androgenic events associated with testosterone, mainly increased non-scalp hair growth, and a neutral or slightly increased risk of breast cancer. Trials to date have been either of insufficient size to either determine or exclude a causal relationship for cancer risk. The ES guideline for women prescribed T-therapy recommended: 1) use of non-oral preparations; 2) monitoring T levels 3-6 weeks and 6 months after initiation of therapy; 3) cessation of therapy for women who have not responded by 6 months; and 4) clinical trials that will measure multiple safety and clinical endpoints. Recent systematic reviews of the efficacy and safety of transdermal testosterone in post-menopausal women have shown small increases in rates of acne and hair growth, but no differences from placebo in other androgenic effects, including alopecia and voice deepening, and no differences from placebo in total adverse events and serious adverse events.(82, 83)

The protocol proposed for this trial is consistent with the recent ES guideline and will provide very important and useful information about clinically meaningful outcomes and the safety of short-term testosterone replacement therapy prescribed for this target patient population. Alternative anabolic agents, such as oxandrolone and oral T have greater safety risks; SARMs and myostatin inhibitors are not clinically available and it will be several years before any are approved for use in the U.S.

Section 6: Study Design

6.1 Overview of Study Design

A randomized, double-blind, placebo controlled, parallel group study in elderly female hip fracture patients will be carried out at multiple clinical sites. All participants will complete an approximate 6-month treatment period and study follow-up visits. Participants will be randomly assigned to one of three study groups:

1. **Enhanced Usual Care** (Home exercise + Health Education) ***(EUC)***
2. **Exercise** (Placebo gel + Supervised Exercise Training) ***(EX)***
3. **Combined** (Testosterone gel + Supervised Exercise Training) ***(EX+T)***

Randomization of 120-168 participants with recent surgical repair of a hip fracture will take place within 6-24 weeks of surgery. Female hip fracture patients age 65 and older at the clinical sites will be evaluated for eligibility. Following consent to participate, eligible participants will undergo a comprehensive screening assessment. Participants who complete the screening assessment and meet all eligibility criteria will be scheduled for baseline visits and randomized to one of the three study groups at the conclusion of the baseline measurements. Figure 1 shows the sequence of participant contacts and study measurements from the time of surgery until the final assessment approximately 9 months post-randomization. Transportation to all study visits and exercise sessions will be offered and provided if desired by the study participant.

Figure 1. Timeline for recruitment, randomization, and follow-up.

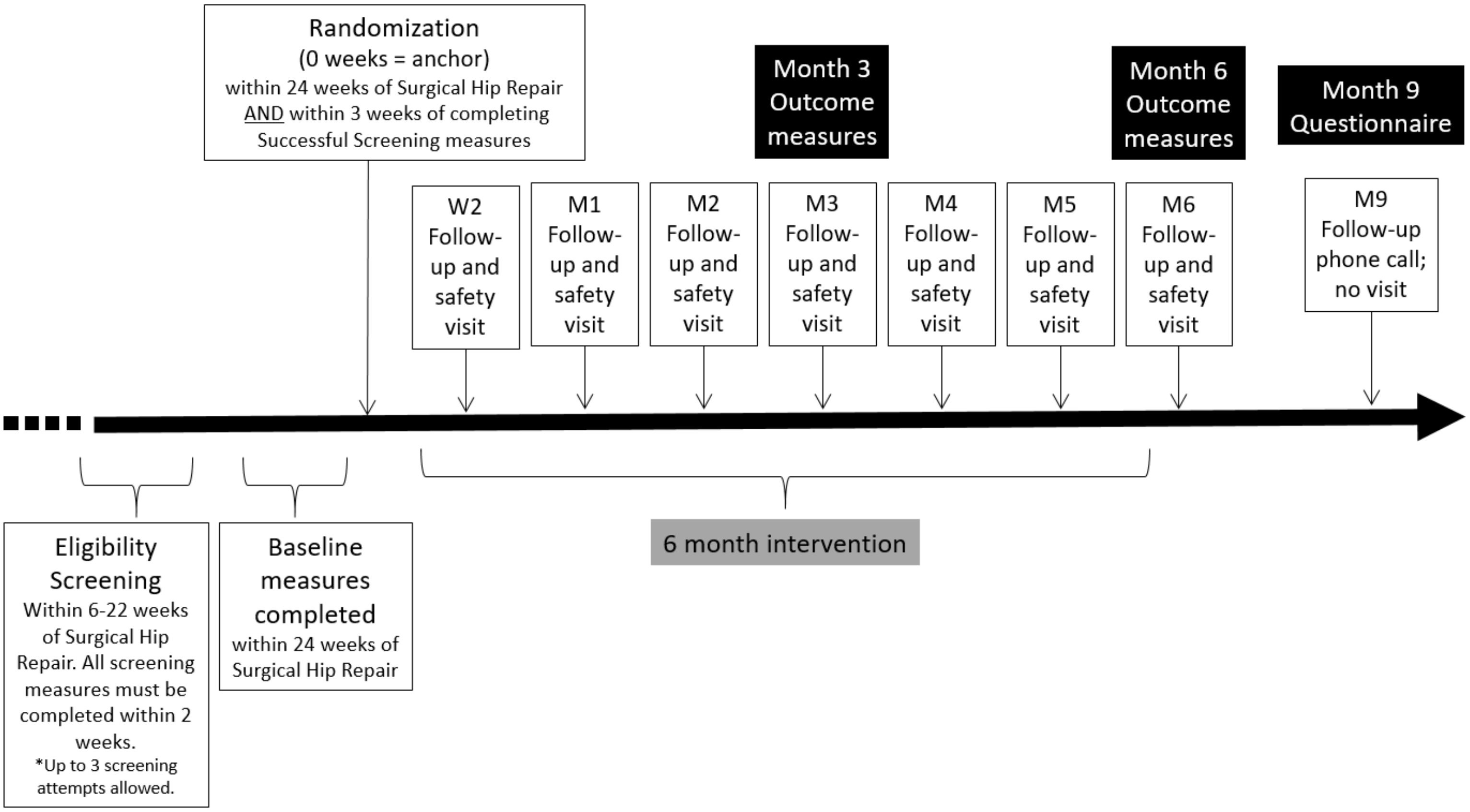

All participants will receive counseling by a dietician to facilitate adequate nutrient intake inclusive of a healthy diet. At the randomization visit, the EX and EX+T participants will receive their first monthly supply of study drug or placebo. For the EX and EX+T participants, initiation of the exercise interventions with an exercise interventionist, who is supervised by a physical therapist (PT), should take place as soon as possible, up to seven days following randomization. The EUC group will be given a home exercise program, contacted weekly (phone or email) by the study staff, and have monthly follow-up visits that will include a health education module.

6.2 Screening

Potential participants will undergo screening 6 to 22 weeks after hip fracture repair surgery. Participants who are beyond 22 weeks past their hip fracture repair surgery may be approached and screened with the approval of the CCC. Subject eligibility will be determined by the following procedures:

- Evaluation of medical history, concurrent illnesses, concomitant medications, and completion of the Angina Questionnaire (SAQ-7);
- Screening for cognitive status and depression using the Short Blessed Test (SBT) and Geriatric Depression Scale (GDS-SF);
- Resting 12-lead electrocardiogram (ECG);
- Physical exam by a site physician or Advanced Practice provider;
- Assessment of physical function using the mPPT; and
- Laboratory Tests: Complete metabolic panel, Complete blood count, TSH, HbgA1c, and Testosterone level.

6.3 Randomization

Randomization will occur between 6 to 24 weeks after the participant's hip fracture surgery. Only women that qualify for the study based on the screening process will be scheduled for baseline assessments and randomization. The study coordinator will assure that all eligibility criteria are documented in REDCap prior to randomization. For more information see Section 12.

6.4 Interventions

Participants in all intervention groups will receive nutritional counseling to promote adequate nutrient intake. Participants in all intervention groups will receive 2,000 IU vitamin D_3 and 1,000 mg of calcium daily for the duration of the 6-month intervention period.

The enhanced usual care group (EUC) will complete a home-based, low-intensity exercise program independently 3 times per week. They will also receive weekly contact by phone or email from the study staff to encourage adherence, answer questions, and keep contact. In addition, the EUC group will attend a health education session once a month. Whenever possible, the health education sessions will be combined with monthly study visits when transportation is provided. The EUC group will not use the testosterone or placebo gel.

Over an approximate 6-month intervention period, participants in the EX and EX+T study groups will complete a supervised multimodal exercise program that includes progressive resistance training sessions. Visits with exercise interventionists will take place at the study center or dedicated exercise facility.

Participants in the EX and EX+T study groups will also receive treatment with either placebo or testosterone gel, respectively. Testosterone and placebo gels will be administered in identical bottles to participants who will be blinded to medication group assignment. All participants will be instructed in proper application of the gels by the study staff.

6.5 Source Documentation

Source documents include original records of clinical observations, laboratory reports, DXA images, radiology reports, ultrasound reports, and medical records including: progress notes, clinic notes, hospital charts, and other records or reports of procedures performed during the study period. The clinical sites are required to retain all source documentation in the participant's research binder. When applicable, data entered into REDCap must be verifiable in the source documents maintained by the clinical sites.

6.6 Efficacy and Safety Measures

Primary and secondary outcome measures will be assessed at baseline, month 3, and month 6.

Primary efficacy measure: Six Minute Walking Distance (6MWD)[83-85]

Secondary efficacy measures: Total and Appendicular Lean Body Mass (LBM, aLBM) as measured by Dual Energy X-ray Absorptiometry (DXA), Total Body and Total Femoral Bone Mineral Density (BMD) of the unfractured limb as measured by DXA, 1-RM strength for leg press, Modified Physical Performance Test Score (mPPT)[86], Short Physical Performance Battery (SPPB)[87, 88], 50 ft. and 8 ft. Gait Speed (each performed as part of mPPT and SBBP, respectively), Functional Status Questionnaire (FSQ)[89], Older Americans Resources and Services (OARS)[90] questionnaire for Activities of Daily Living (ADL), Hip Rating Questionnaire (HRQ)[91], PROMIS® Global Health[92], and Brief Resilience Scale (BRS)[93].

To address Aim 3, which is exploratory, a limited number of secondary outcome measures will be obtained by telephone interview at month 9: 1) Functional Status Questionnaire (FSQ), Older Americans Resources and Services (OARS) questionnaire for Activities of Daily Living (ADL); 2) Hip Rating Questionnaire (HRQ); and 3) PROMIS® Global Health.

Safety measures: Participants will be carefully monitored for the development of side effects and potential health problems related to the interventions. Adverse Event (AE) assessments, physical examinations, and laboratory testing will be performed at prescribed intervals to monitor for exercise and medication side effects and will be reviewed by the clinical site physician. Following the intervention period, a mammogram and transvaginal ultrasound will also be performed to monitor for medication side effects.

6.7 Blinding of Study Staff

All study staff will be blinded to investigational drug treatment assignment. Because of the structure of the exercise programs and the importance of making the interventions accessible to the target population, it will not be feasible to blind study participants and all staff to the exercise

interventions. The staff responsible for administering the interventions will be blinded to participants' study outcomes. Clinical site PIs and clinical site coordinators who are responsible for assigning work and/or assessing for treatment fidelity of exercise interventionists in both groups will be unblinded to exercise intervention assignments, but not involved in assessments of study outcomes.

To the extent possible, the research staff administering the outcome assessments will be blinded to exercise intervention group. Blinded study staff performing follow-up assessments will be instructed not to ask about treatment (exercise) assignment when in contact with participants. Participants will be instructed not to discuss their treatment experience (including the identity of the exercise interventionist) during study visits and telephone calls conducted by blinded staff.

Only designated members of the DCC staff and members of the DSMB will see group-specific study results. In the event of inadvertent unblinding of study staff, a protocol deviation will be reported and a notation will be made in the participant binder specifying the circumstances of the unblinding event and a list of all persons unblinded.

Section 7: Eligibility

Following initial identification of participants by referral and/or chart review, participants will be evaluated for eligibility at two time points: a pre-screen and a study screening visit. Participants will be followed between pre-screen and screening to allow for additional assessment of eligibility prior to formal screening. Pre-screening will assess eligibility of the potential participant on a selection of the medical history inclusion and exclusion criteria. Pre-screening questions will be collected using a standardized interview that can be administered over the telephone or in-person. Other criteria will be reviewed from the participant's medical record. If the participant appears to meet the pre-screening criteria, a more detailed screening visit will involve assessment for medical, safety, and feasibility criteria. The pre-screening and screening visit should be completed within 22 weeks of surgical hip repair. Participants who are beyond 22 weeks past their hip fracture repair surgery may be approached and screened with the approval of the CCC.

7.1 Inclusion Criteria

1. Female 65 years and older.
2. Surgical repair of a non-pathologic fracture of the proximal femur (Including: femoral neck or intracapsular, intertrochanteric, and subtrochanteric fractures), with a surgical repair date that is within 24 weeks at randomization. If a revision of such a fracture is performed due to failure of the repair, that surgery revision date may be used to calculate the time frame for the screening and randomization dates.
3. Functional impairment at the time of screening, defined as a modified Physical Performance Score (mPPT) of 12-28.
4. Serum total testosterone level <60 ng/dL.
5. Community-dwelling or in assisted living prior to the hip fracture event.

7.2 Exclusion Criteria

1. Use of progestin or androgen containing compound within the previous 6 months.
2. Cognitive impairment or dementia of severity sufficient to interfere with ability to fully participate in the study or provide one's own informed consent, or a score of ≥11 on the Short Blessed Test of Orientation, Memory and Concentration[94, 95].
3. Visual or hearing impairments that interfere with following directions for research procedures.
4. Active or unstable cardiopulmonary disease (recent MI, unstable angina, class III or IV CHF) within prior 6 months, which would limit full participation in this study.
5. Uncontrolled hypertension, defined as a systolic BP > 160 mm Hg or diastolic BP > 95 mmHg, on at least two occasions.
6. Respiratory disease requiring chronic continuous oxygen therapy, or oxygen therapy during walking or exercise, which would limit full participation in this study.
7. History of: a) Breast, ovarian, endometrial or cervical cancer with diagnosis within the previous 10 years; b) Breast, ovarian, endometrial or cervical cancer of Stage 2 or higher.
8. Elevated liver transaminase or alkaline phosphatase levels ≥ 2.5 times above normal range.
9. Erythrocytosis defined as hematocrit ≥ 51% at all sites but UCD and UofU, or ≥ 52% at the UCD and UofU sites.
10. Severe anemia defined as Hgb < 7 gm/dL.
11. History of HIV or active viral hepatitis.

12. Uncontrolled diabetes defined as HgbA1C>10%.
13. GDS-SF score ≥ 12 at the screening assessment.
14. Recent history of alcohol or substance abuse, or current alcohol intake of ≥ 10 drinks/week.
15. Untreated or unstable thyroid disease, with serum TSH level ≥ 10 mIU/L or TSH level ≤ 0.4 mIU/L. Levels outside of the given range require site physician documentation addressing treatment or absence of thyroid disease and approval by the CCC.
16. Current use of aldactone, flutamide or leflunomide.
17. Treatment with systemic corticosteroids (daily dose > 5 mg prednisone or equivalent) for at least 90 days within the previous 12 months.
18. Musculoskeletal or neurological conditions that limit full participation in this study, could be made worse by exercise training, or not expected to improve with exercise.
19. End Stage Renal Disease on dialysis or GFR<15 ml/min.
20. History of idiopathic deep venous thrombosis or pulmonary embolus (i.e., not related to period or immobilization or surgery), any pulmonary embolus less than 12 weeks prior to the first screening visit, recurrent or multiple venous thrombi; history of a hypercoagulable state such as Factor V Leiden thrombophilia.
21. Lower extremity amputation other than toes.
22. Planned joint surgery during the intervention period.
23. Severe lower extremity pain or ulceration that could limit full participation in this study.
24. Allergy to gel components.
25. Residence too far from research center (specific distance to be determined by each site), or planned travel greater than 2 weeks within the next 9 months.
26. Anticipated to be permanently living in a nursing home at the time of randomization.
27. Site investigator’s judgment that the participant would not be able to complete research procedures or interventions.
28. Participation in another research study that in the site investigator’s judgment could interfere or conflict with STEP-HI research assessments or interventions.

7.3 Concomitant Medications

The name of any prescription and over-the-counter medication that the participant is taking at the time of enrollment as well as any additional prescription and over the counter medication used during the study will be documented in the participant’s study record. Medications will be reviewed with participants monthly at each study follow up visit.

7.3.1 Prohibited medications

Treatment with the following concomitant medications is prohibited during study:

- Androgen, progestin, or any other anabolic agent
- Aldactone
- Flutamide
- Leflunamide
- Treatment with systemic corticosteroids at a daily dose of >5 mg prednisone or equivalent for a duration of 90 days or longer within the previous 12 months

If a patient takes a prohibited medication during the study, the site must notify the CCC. The decision to discontinue or to keep the patient in the study will be made after consultation

between the site PI and the Study Chair. The dose and duration of the prohibited medication used will be a factor in the decision.

7.3.2 Pharmacologic Agents for Osteoporosis

Based on previous hip fracture studies, we anticipate that 30-50% of the patients enrolled will be taking bisphosphonates for osteoporosis, and some will be started on them during the intervention period. We will record medication use at baseline and monthly during the 6 month intervention period. The information will be used to interpret results. Since these medications should be distributed equally among the three study groups, we do not anticipate that they will affect the intention-to-treat analyses.

Section 8: Recruitment and Retention

8.1 Identification of Hip Fracture Patients

Potential participants will be identified from local site acute care hospitals (orthopedic and rehabilitation units), skilled nursing facilities, home care programs, and the community-at-large. The target sample size is between 120 and 168 randomized participants. In order to achieve this target, it will be necessary to identify more than 4000 hip fracture patients across all sites. This estimate is based on previous experience conducting clinical trials with the proposed interventions in hip fracture patients, with similar eligibility criteria to the current study.

8.2 Participant Recruitment and Pre-Screening

Each site PI and/or coordinators must ensure a systematic method to identify potential participants at their center. Specific strategies will include discussion of the study with the local orthopedic surgical team, screening at affiliated rehab centers, and gaining access to a hospital database by engaging a bioinformatics or information technology group to locate patient's status post hip surgery based on ICD 10 or CPT code all subject to IRB approval. Recruitment fliers will also be given to inpatients referred by hospital or rehab staff, physicians, or surgeons.

Those volunteers who may be eligible for participation in the study will be administered a brief pre-screening interview either by telephone or in person. Those who meet eligibility criteria after the pre-screening interview will be invited to undergo a more detailed screening evaluation near the end of her course of standard physical therapy.

Once a potential subject is identified, the investigator and/or coordinator will screen the medical history including medications and laboratory studies to confirm that the patient may be eligible. If the patient's physician or a research team member believes that the patient is cognitively impaired or otherwise unable to provide informed consent, the patient should not be invited to participate. All subjects should be able to understand the nature of the research and their participation, and show the ability to consider the alternatives including the option of non–participation. Patients who agree to consider participation should provide their contact information. Potential participants will then be contacted periodically through their course of standard rehabilitation to build rapport and determine when they are ready to be screened for the study.

To guide recruitment efforts and methods, participants who refuse to complete the pre-screening interview or refuse consent following the pre-screening interview will be asked reasons for refusal.

If the participant refuses participation, but agrees to be contacted further, the CCC will follow-up with a qualitative interview to gather more information about reasons for refusal. The information collected will be kept in an anonymous fashion and will not have any identifiers or patient data associated with it.

Recruitment will last for approximately 45 months, with a goal for each clinical site to randomize 1-2 participants per month.

8.3 Informed Consent

Written informed consent and appropriate HIPAA authorizations and/or waivers will be obtained in compliance with procedures reviewed and approved by the site IRBs and the sIRB at Washington University. Each of the clinical sites' IRBs will review and agree to rely on Washington University's sIRB prior to any data collection. Study staff will follow the Good Clinical Practice guidelines for informed consent. A copy of the informed consent form (**ICF**) will always be provided (either in person or by mail) to potential participants to allow for adequate review of the information and to allow review with family members. Prior to obtaining informed consent, time will be given to the potential participant to review the consent form and ask questions. Study staff will summarize all components of the ICF and remind potential participants that participation in the study is voluntary and that she has the right to withdraw at any time. It will also be explained that signing the consent form allows the study to confirm final eligibility before randomization to a treatment group. The consent process will be performed without the use of any coercive language or behavior, and with respect for the person's autonomy.

The goal of the informed consent process is to increase potential participants' understanding of the objectives and expectations of the study in order to better enable them to decide whether or not to enroll. Therefore, every effort will be made to help potential participants fully understand the research project. During the informed consent process, study staff will provide participants with adequate information concerning the study procedures, respond to questions and concerns, and ensure that each individual understands all the information provided.

Potential participants who choose to enroll will be asked questions about the main parts of the study to assess their understanding of the expectations, risks, and benefits. The researchers will ensure the potential participant has decisional capacity by asking the participant to reply to the following questions after the consent has been explained: 1) Why is this study being done?, 2) What are the risks of participating in this study?, 3) What are the potential benefits of participating in this study?, 4) Is the study voluntary?. Based on the responses to these questions, the person conducting the informed consent will determine whether the potential participant has decisional capacity. Any questions/concerns about a participant's ability to provide consent will be discussed with the site PI. Individuals who do not understand the study purpose, risks, and benefits are not able to provide their own informed consent and will not be eligible for participation. The informed consent process will be documented on the Documentation of Informed Consent Form. The form confirms that the study was explained, ample time was given to read and discuss the ICF, questions (if any) were answered, subject agreed to participate and signed the ICF and HIPAA Authorization (if separate), and a copy of the signed ICF was given to the participant. If necessary, a copy of the ICF can be left in the chart of the facility based on site policy. For women who provide informed consent, the original signed ICF will be maintained at the clinical site and a copy of the signed ICF will be given to the participant. The clinical site PI and/or designee will ensure the accuracy, completeness, legibility, and timeliness of the informed consent process conducted by the study staff before any screening data are collected.

8.4 Enrollment

Date of enrollment in this trial is defined as the date of informed consent.

8.5 Screening

After signing the informed consent form, screening assessments will be conducted with each participant. The purpose of the screening assessments is to identify any contraindications to exercise training or testosterone therapy and other exclusion criteria. Screening assessments may occur any time after informed consent is obtained and prior to randomization, but these tests should be performed as early in the visit window as possible. Re-screening will be allowed if the reason for screen failure can be realistically corrected within 22 weeks of the hip surgery.

Screening is performed in stages so that an individual with a contraindication to study participation does not undergo unnecessary tests. Screening assessments will include a review of all disqualifying medical conditions, assessment of cognitive status with the Short Blessed Test (SBT), and assessment of mood with the Geriatric Depression Scale Short-form (GDS-SF). Activities of Daily Living (ADLs) prior to the hip fracture will also be assessed. Blood will be collected at screening and the following tests performed: complete metabolic panel, complete blood count, TSH, hemoglobin A1c, and serum testosterone. An ECG and full physical exam will be performed. The study coordinator will notify the participant's primary care physician and orthopedic surgeon of her interest in the study, obtain answers to questions about the participant's medical history, and answer questions that the patient's physicians may have about the study.

The screening visit may be completed in up to two separate sessions with the first session occurring in the participant's home, remotely, or over the phone at the discretion of the clinical site coordinator. See section 10.1 for the list of measures and acceptable collection locations. An abbreviated mPPT may be completed in the participant's home if she appears too frail to meet eligibility criteria. If an abbreviated mPPT is completed, the full 9 item mPPT assessment must still be completed at the clinical center.

If the physician determines that performance of the mPPT or intensive exercise poses a cardiac risk for the participant, then she will be referred to her PCP for further testing (i.e. exercise stress test) and clearance. Participants may also be excluded if, in the investigator's opinion, there is evidence of cognitive impairment or dementia which is sufficient to interfere with informed consent or adherence to the study protocol. The clinical site physician must review all study eligibility documents and sign off on final eligibility for all potential participants prior to collection of baseline data. Section 10.1 Screening Assessments contains a detailed inventory of screening assessments that will be completed.

8.6 Retention Promotion Efforts

At the time of randomization, participants will receive written instructions in a study folder about the schedule of follow-up assessments, exercise or health education visits, dietary assessment, and phone contact numbers for site study staff. To maintain rapport, the same staff member for a given study role (coordinator, exercise interventionist, etc.) will, whenever possible, contact the participant throughout the study. Minimizing waiting time, providing transportation at no charge, streamlining visit activities, and providing comfortable waiting room facilities makes the visits more pleasant, thereby enhancing participant retention at follow-up assessments.

Participants in the EUC group will be contacted weekly by telephone in an effort to maintain rapport and provide some increased social contact. Regular phone contact also aids retention.

Participants will receive reminders prior to each follow-up assessment.

Clinical sites will keep detailed records of rescheduled and missed study assessment visits as well as missed intervention visits. Participant retention will be monitored, and efforts will be made to provide support and encouragement to participants who are at risk of being lost to follow-up.

8.7 Drop-out Prevention Efforts

The following procedures will be implemented to minimize and monitor missed follow-up assessments and intervention visits:

- Providing pre-visit reminders (e.g., letters and phone calls) for upcoming assessment visits.
- Calling participants the day before a follow-up assessment to remind them of the visit and the time for transportation pick-up, if appropriate.
- Providing transportation to assessment and intervention visits.
- Monitoring participation and attendance within each clinical site by the DCC, so that staff will be alerted to a missed intervention or assessment visit.
- Rescheduling the visit within the visit window, if possible.
- Monitoring exercise attendance weekly and contacting participants who miss sessions to discuss reasons and help them problem-solve barriers to adherence that are identified.
- Monitoring gel application adherence monthly (bottle weights), observing application technique monthly, and discussing any problems or barriers to adherence that are identified.

Some randomized participants may not actively participate in the study, either by declining the intervention visits and/or by not attending follow-up assessments. These participants will be followed until the end of the study unless they explicitly request not to be contacted. Also, considerable effort will be expended to collect data on the primary outcome at each of the follow-up assessments.

8.8 Premature Withdrawal from the Study

A subject has the right to withdraw from the study at any time, for any reason, and without repercussion.

Any participant who is randomized, should be encouraged to continue in the study for safety monitoring and outcome assessments at the specified time points in the protocol. If a participant does not wish to complete the remaining outcome assessments, it will be proposed to only complete the 6 month assessment or return to the research center for an early withdrawal visit. An early withdrawal visit would be scheduled as soon as possible and include prioritized outcome measures that allow the participant to stop at any point or skip any measures.

The site PI has the right to discontinue an intervention or a participant from the study in the event of an intercurrent illness, serious adverse event (SAE), protocol violation, or other reasons. See Section 18: Discontinuation for more information. An excessive rate of

withdrawals and discontinuations would render the study uninterpretable; therefore, unnecessary discontinuation of participants should be avoided.

8.9 Replacement of Subjects

Subjects who withdraw from the study or are prematurely discontinued from the study will not be replaced.

8.10 Monitoring Recruitment and Retention

The DCC, in collaboration with the CCC, will monitor recruitment and retention. The DCC will track screening and recruitment yields against the number expected per site. The DCC will work closely with clinical site PIs and the CCC to identify and reduce barriers to recruitment within either clinical sites or the study hospitals. The number of patients screened relative to the number of participants recruited in each facility will be reviewed quarterly.

8.10.1 Monitoring and Quality Control of Recruitment and Retention

The DCC will collect data to monitor recruitment and retention activities, the number of potential participants screened at each site, the yield at the various screening phases, the percent who are eligible, and the percent who are randomized. This monitoring will allow early detection of site recruitment and retention issues. Reports from the database can also be generated according to individual study staff member to identify possible areas for retraining. Retention will be monitored through completion rates of follow-up assessment visits and intervention visits. Regular reports will be available to clinical sites and the CCC. The CCC coordinator will maintain regular phone contact with clinic staff to:

1. Review recruitment goals and yields for clinical sites,
2. Review the recruitment plan and progress in achieving the objectives outlined in the plan,
3. Share successful and unsuccessful recruitment methods, and
4. Review retention.

If clinical sites encounter difficulties in recruitment or retention, the CCC (or a subgroup it designates) will provide a graduated set of assistance responses that are based on the degree of recruitment or retention shortfall. Solutions will be developed that are based on site-specific issues.

Section 9: Study Measures

9.1 Physical and Questionnaire Measures

Detailed instructions on administration and documentation forms can be found in the Manual of Procedures. These evaluations will be conducted during an in-person, or when feasible by remote video call, or phone interview and are not presented in order of administration.

9.1.1 Demographics

Basic demographic information will be collected from the participant by a study staff member and entered into the Demographics study form.

9.1.2 Medical History

A study staff member will record the participant's medical history through a series of yes or no questions about previous diagnosis and past or current health issues. The answers will be recorded on the Medical History Form. Input from the site physician can also be obtained to complete the questionnaire. Information will also be collected on the type of hip surgery for the Hip Fracture History Form. If questions arise, permission will be requested to obtain medical records from the participant to complete the information.

9.1.3 Medications or Medication Updates

Study staff will collect all medications (prescribed and regularly used over-the-counter) that the participants are currently taking and those in the recent past that may interfere with the active medication for the current study. Medications will be reviewed with the participant monthly so records can be updated.

9.1.4 Height, Weight, and Vital Signs

Study staff will record the height and weight of the participant to track for changes. Weight will be measured monthly in kilograms by scale. Height will be measured in centimeters at the baseline, month 3, and month 6 visits. Blood pressure and pulse will be measured at each monthly visit, as well as before and after center-based exercise sessions.

9.1.5 Physical Examination

A physical examination by the clinical site PI, a designated physician, or an Advanced Practice provider will be conducted at the study center for screening and follow-up purposes. The examination should be considered a routine physical examination but will include a detailed neurological and musculoskeletal evaluation. A manual breast examination is required at screening if the participant has had a mammogram within the past 6 months and therefore is not required to undergo a mammogram during baseline assessments (per Section 9.3.4).

9.1.6 Short Blessed Test (SBT)

All participants will have their cognitive function evaluated at screening using the Short Blessed test of Orientation, Concentration, and Memory (SBT). Those participants with a score of 11 or greater on the test will be excluded from the study. In the event that the consenting party has concerns about a participant's ability to consent, the SBT will be administered to measure cognitive function before proceeding with other screening measures.

9.1.7 Activities of Daily Living (ADLs)

Information about performance of activities of daily living (ADLs) will be collected using two scales that have been well validated for use with older adults in a variety of outpatient settings, and with which we have extensive experience in the hip fracture population: the Duke Older Americans Resources and Services (OARS) scales, and the Functional Status Questionnaire (FSQ). The two scales provide complementary information about ADL performance. An Assistive Devices Checklist (ADC) has also been developed to be administered with the ADL measures. A modified OARS instrument will be administered at screening to assess abilities prior to the hip fracture. The ADL questionnaires will be administered at baseline, 3 months, and 6 months to look for changes in ADLs. Based on clinical site resources and participant availability, the ADL questionnaire will be administered via a telephone call at 9 months.

9.1.7.1 Older Americans Resources and Services (OARS)

The OARS queries about requirement for assistance (human or assistive device) during daily activities.

9.1.7.2 Functional Status Questionnaire (FSQ)

The FSQ queries about amount of difficulty experienced during daily task performance during the past 4 weeks. Since the tasks are the same listed in the OARS the questionnaires have been combined to assess if a task was completed with assistance and how difficult it was to complete the task.

9.1.8 Geriatric Depression Scale – Short Form (GDS-SF)

Participants will be screened for depression using the Geriatric Depression Scale – Short Form (GDS-SF). Participants will be excluded and referred to their PCP if their GDS-SF score is ≥ 12 at screening (on or off treatment). The GDS-SF will also be administered at baseline, month 3, and month 6.

9.1.9 Seattle Angina Questionniare-7 (SAQ-7)

The SAQ-7 asks about limited activities and the amount of chest pain or tightness participants have had over the previous 4 weeks. This questionnaire will be administered at screening to assess if participants are at higher cardiac risk during exercise training.

9.1.10 Hip Rating Questionnaire (HRQ)

Participants will be administered the Hip Rating Questionnaire (HRQ) at baseline, month 3, month 6, and month 9. The HRQ is a 14 item inventory of patient reported pain level, mobility, and activities of daily living directly related to the hip fracture event.

9.1.11 PROMIS® Global Health

The 10-item PROMIS® Global Health instrument has Global Physical Health and Global Mental Health components to measure overall quality of life. Patient-Reported Outcomes Measurement Information System (PROMIS®) was recently developed and validated to be psychometrically accurate to be used with the general population, as well as individuals with chronic conditions. The measure will be administered at baseline, month 3, month 6, and month 9.

9.1.12 Brief Resilience Scale (BRS)

The Brief Resilience Scale (BRS) measures self-perceived psychological resilience, defined as the ability to bounce back after a stressful event. The BRS includes 6 statements related to the construct of bouncing back after a stressful event, and the 5 response options for each statement range from “strongly agree” to “strongly disagree”[(91)].

The role of psychological resilience among older women following hip fracture has received little scientific attention. We hypothesize that level of psychological resilience will have an independent effect on primary and secondary outcomes in the STEP-HI study. Administering the BRS at study baseline and month 6 will allow us to observe the degree to which resilience might change over time in relation to treatment group membership in the STEP-HI study. To our knowledge, the degree to which participation in a supervised exercise program and use of testosterone might improve level of psychological resilience in older women after hip fracture has not been studied.

9.1.13 Ferriman-Gallwey Scale

The Ferriman-Gallwey Scale will be utilized to look for hair growth that may result from testosterone gel usage. A limited physical exam will be done by the site physician or Advanced Practice provider to complete the scale at the baseline, 3 month, and 6 month assessments.

9.2 Physical Performance Measures

Detailed instructions on administration of measures are in the study Manual of Procedures. These measures will be administered by certified study staff that are blinded to the extent possible to exercise group assignment.

9.2.1 Six Minute Walking Distance (Primary Outcome Measure)

Walking endurance will be measured using a 6-Minute Walk Distance at baseline, month 3, and month 6. Participants will be asked to walk back and forth on a measured path marked clearly at both ends for turning purposes, while being told when each minute has passed, and receiving verbal encouragement (“you’re doing well” or “keep up the good work”) every 60 seconds.

9.2.2 Leg Press 1-Repetition Max Test

Muscle strength will be measured as the maximal amount of weight that the participant is able to lift for one repetition (1-RM). 1-RM will be obtained for leg press of the fractured and unfractured limb simultaneously, using identical weight-lifting equipment across clinical sites that will be purchased and distributed by the CCC. 1-RM assessments will be performed at baseline, month 3, and month 6.

Subjects will be asked to move against progressively heavier resistance loads until a repetition failure is achieved. A rest period will separate each repetition. A repetition failure will be defined as 1) the inability to move against the resistance to the required range of motion, or 2) not using proper technique, or 3) the subject not feeling safe in trying a heavier resistance. The 1-RM is defined as the greatest resistance that could be overcome through a defined range of motion using proper techniques.

9.2.3 Modified Physical Performance Test (mPPT)

A modified version of the Physical Performance Test (mPPT) will be used to measure physical function at screening. The modification substitutes a chair-rise task (See SPPB) and a balance task (See SPPB) for writing and eating tasks, in order to emphasize lower extremity function. The modified PPT includes nine standardized tasks. Seven tasks are timed: book lift, putting on a lab coat, picking up a penny from the floor, standing balance (progressive Romberg), standing up five times from a 16-inch chair, 50 foot walk including a turn, and timed stair climb for 10 steps. Two tasks are not timed: 360 degree turn and climbing 2 additional flights of steps. The score for each item ranges from 0 to 4, with 36 representing a perfect score. Test-retest reliability for the modified PPT score is 0.96[96, 97]. Because there is some overlap between the mPPT and SPPB items, we have integrated the two scales so that participant burden is minimized but it is still possible to obtain scores on each of the scales. Participants with a score of 12-28 are in the acceptable range to be included in the study; others will be excluded. The mPPT will also be completed at baseline, 3 months and 6 months.

9.2.4 Short Physical Performance Battery (SPPB)

The Short Physical Performance Battery (SPPB) is another well validated objective physical performance measure that has been used in a number of epidemiological and intervention trials in older adults to identify risk for disability[87, 88]. The SPPB will be completed at baseline, month 3, and month 6. It consists of three items, two of which are performed as part of the mPPT: a chair rise task and a progressive Romberg balance test. Gait speed for 4 meters is performed as a separate item from the mPPT tasks (see below).

9.2.4.1 Four Meter Walk (Gait Speed)

Gait speed will be measured over a 4 meter distance on a standard walkway at baseline, month 3, and month 6. Participants are timed on the speed at which they walk the 4 meter distance, both at their preferred or normal speed and for a “fast walk.” Use of walking aids is allowed.

9.2.5 Hand Grip Strength

A hand grip dynamometer will be used to measure the maximum isometric strength of the hand and forearm muscles at baseline, month 3, and month 6. Sites may use equipment available at their site, though a Jamar device is preferred.

9.3 Other Procedures

9.3.1 Laboratory Blood Tests

Blood will be drawn to check total blood count (CBC), blood chemistry (CMP), liver enzymes, thyroid hormone level (TSH), glucose (HgbA1c), Cholesterol (Total, LDL, and HDL), estradiol level, sex hormone binding globulin (SHBG) level, Vitamin D levels (serum 25-OH), and Testosterone (total and free) levels. Not all labs will be drawn at each time point. Labs will not be identical across all randomized groups; the EUC group will not have monthly testosterone draws. Only the testosterone level of the EX and EX+T group will be drawn at week 2. For further details, see **Table 2: Assessment Schedule**.

9.3.2 Electrocardiogram (ECG)

A standard 12-lead electrocardiogram (ECG) will be performed and evaluated locally at each clinical site at screening. The ECG will be performed at the study center. The ECG will allow

the investigator to assess patients for any abnormalities that, in the investigator's opinion, could increase the patient's risk of participating. ECGs will be interpreted by the clinical site PI or a qualified designee, who will document their review. ECG reports should be retained with source documentation.

9.3.3 Dual Energy X-Ray Absorptiometry (DXA)

Appendicular and total LBM and bone mineral density of the proximal femur of the unfractured limb will be determined by Dual Energy X-Ray Absorptiometry (DXA) scanning. Whole body measurements will be taken at baseline and month 6. Baseline scans should be completed prior to dispensing of study drug to participants. DXA scans should always be performed before exercise or 1-RM measurements.

All DXA imaging scans obtained will be transferred to the IMAGE center at UCD for quality control. DXA scans will be reviewed for proper positioning and artifacts within one week of image acquisition. Sites will be immediately notified about significant problems and a repeat scan will be scheduled. DXA imaging scans obtained using Hologic scanners will be analyzed by the IMAGE center.

9.3.4 Mammogram

A Mammogram will be completed at baseline if the participant hasn't had one in the past 6 months or if documentation of a mammogram in the past 6 months is not available. The mammogram will be repeated at month 6 as a safety assessment for the supervised exercise groups (EX and EX+T) only.

9.3.5 Transvaginal Ultrasound

For women who participated in the EX or EX+T group and have a uterus, a transvaginal ultrasound will be completed at the month 6 assessment. The ultrasound will measure the lining of the uterus. In cases where the measurement exceeds 4 mm, participants will be referred to their gynecologist for possible further assessment and treatment.

Section 10: Assessment Schedule

Table 2: Schedule of Assessments

Study procedure	Screening	Baseline	W2	M1	M2	M3	M4	M5	M6	M9
**Physical and Questionnaire Measures**										
Demographics	x									
Medical History	x									
Angina Questionnaire (SAQ-7)	x									
Medications or medication updates	x	x		x	x	x	x	x	x	
Height		x				x			x	
Weight, Pulse, Blood pressure	x	x		x	x	x	x	x	x	
Physical exam	x	x				x			x	
Short Blessed Test (SBT)	x									
Hip Rating Questionnaire (HRQ)		x				x			x	x
Brief Resilience Scale (BRS)		x							x	
Geriatric Depression Scale (GDS-SF)	x	x				x			x	
Pre-fracture Activities of Daily Living (ADLs)	x									
Older Americans Resources & Services (OARS)		x				x			x	x
Functional Status Questionnaire (FSQ)		x				x			x	x
Assistive Device (ADC)	x	x				x			x	x
PROMIS - Global Health		x				x			x	x
Dietary Assessment		x				x				
Ferriman-Gallwey Scale		x				x			x	
Adverse Event (AE) Assessment		x		x	x	x	x	x	x	
Fall Log		x		x	x	x	x	x	x	
Exercise Log		x		x	x	x	x	x	x	
**Functional Measures**										
Physical Therapy Exam		x								
Modified Physical Performance Test (mPPT)	x	x				x			x	
Six Minute Walk Distance (6MWD)		x				x			x	
Leg Press 1-RM		x				x			x	
4 Meter Walk (SPPB-Gait speed)		x				x			x	
Hand Grip Strength		x				x			x	
**Procedures**										
ECG	x									
DXA Scan		x							x	
Mammogram<br>(**only for EX & EX+T at M6)		x							x**	
Transvaginal Ultrasound<br>(only for EX & EX+T AND if uterus present)									x	
**Laboratory**										
Testosterone Total & Free Levels (EX & EX+T)	x		x	x	x	x	x	x	x	
Testosterone Total & Free Levels (EUC)	x					x			x	
Complete Metabolic Panel (CMP)	x					x			x	
Complete Blood Count (CBC)	x					x			x	
TSH	x									
HgbA1c	x									
Estradiol Level		x							x	
SHBG Level		x							x	
Cholesterol (Total, LDL, and HDL)		x							x	
25-OH Vitamin D Level		x							x	

10.1 Screening Assessments

The screening assessments are performed no earlier than six weeks and no later than 22 weeks after the hip fracture repair. Participants who are beyond 22 weeks past their hip fracture repair surgery may be approached and screened with the approval of the CCC. The screening visit can be divided into two visits and completed at the clinical site center or as noted below, some of the assessments may be able to be completed by phone, via remote video call, or in the participant's home depending on site resources and availability. The following items will be completed at screening (See **Table 2: Assessment Schedule**):

- Demographics (In home, by phone or video call, or onsite)
- Medical History (In home, by phone or video call, or onsite)
- Medications (In home, by phone or video call, or onsite)
- Weight & Vital Signs (Onsite)
- Complete Physical Exam (In home or onsite)
- Short Blessed Test (SBT) (In home, by phone or video call, or onsite)
- Pre-Fracture Older Americans Resources and Services (PFOARS) Questionnaire (In home, by phone or video call, or onsite)
- Geriatric Depression Scale (GDS-SF) (In home, by phone or video call, or onsite)
- Angina Questionnaire (SAQ-7) (In home, by phone or video call, or onsite)
- Modified Physical Performance Test (mPPT) (Onsite)
- ECG (Onsite)
- Blood Draw (In home or onsite) for the following labs:
    - CMP
    - CBC
    - TSH
    - HgbA1c
    - Serum Testosterone Level (Free and Total)

10.2 Baseline Assessments

The baseline assessments may be performed between 6 and 24 weeks after the surgical repair of the hip fracture, but must allow for randomization to occur within 24 weeks of the surgical repair. All baseline assessments must be completed within 3 weeks of the completion of the screening assessments. The CCC may approve a short extension (roughly 1 week) in cases where mammogram, DXA, blood results, or other tests need follow-up. Multiple visits will be allowed to complete the baseline assessments, but ideally all baseline components will be completed in 3 or fewer visits. As noted below, some of the assessments may be able to be completed by phone, via remote video call, or in the participant's home depending on site resources and availability. Randomization can occur when all baseline assessments are completed and eligibility is verified. Once a participant is randomized, that date becomes the anchor (day 0). The following measures will be assessed at the baseline visit(s) (see **Table 2: Assessment Schedule**):

- Medication updates (In home, by phone or video call, or onsite)
- Height, Weight and Vital Signs (Onsite)
- Six Minute Walk Distance (Onsite)
- DXA (Onsite)
- 1-Repetition Maximum (Onsite)
- Modified Physical Performance Test (mPPT) (Onsite)

- 4 Meter Walk (SPPB - Gait Speed) (Onsite)
- Hand Grip Strength (Onsite)
- Hip Rating Questionnaire (HRQ) (Onsite, if possible)
- PROMIS® Global Health Questionnaire (Onsite, if possible)
- Brief Resilience Scale (BRS) (Onsite, if possible)
- Older Americans Resources and Services (OARS) Questionnaire (Onsite, if possible)
- Functional Status Questionnaire (FSQ) (Onsite, if possible)
- Geriatric Depression Scale Short form (GDS-SF) (Onsite, if possible)
- Dietary assessment and consultation (In home, by phone or video call, or onsite)
- Mammogram (if not completed in the past 6 months) (Onsite)
- Physical exam and completion of Ferriman-Gallwey Scale (In home or onsite)
- Physical Therapy Exam (In home or onsite)
- Blood draw (In home or onsite) for the following lab tests:
    - 25-OH Vitamin D
    - Serum Estradiol
    - Serum SHBG
    - Serum Lipids

10.3 Follow-up Assessments

The following measurements will be collected as part of the follow-up visits. Follow-up visits will begin at 2 weeks (target 14 days) for the EX and EX+T participants and then at month 1 (target 28 days) through month 6 (target 168 days). The monthly visits are targeted for every 4 weeks or 28 days, but allow windows for scheduling. Participant and facility schedules will be taken into account when scheduling assessment visits and are not expected to be conducted on the exact date suggested by the visit title. As noted below, some of the assessments may be able to be completed by phone, via remote video call, or in the participant's home depending on site resources and availability. EUC participants will not have a 2 week visit, but will begin at month 1. The Month 6 assessment will begin after the exercise sessions are completed and will be scheduled as close to 25 weeks after randomization as possible. Not all of these assessments are performed at each visit (See **Table 2: Assessment Schedule**):

- Height, Weight, and Vital Signs (In home, by phone or video call, or onsite)
- Six Minute Walk Distance (Onsite)
- DXA (Onsite)
- 1-Repetition Maximum (Onsite)
- Modified Physical Performance Test (mPPT) (Onsite)
- 4 Meter Walk (SPPB - Gait Speed) (Onsite)
- Hand Grip Strength (Onsite)
- Hip Rating Questionnaire (HRQ) (Onsite, if possible)
- PROMIS® Global Health Questionnaire (Onsite, if possible)
- Brief Resilience Scale (BRS) (Onsite, if possible)
- Older Americans Resources and Services (OARS) Questionnaire (Onsite, if possible)
- Functional Status Questionnaire (FSQ) (Onsite, if possible)
- Geriatric Depression Scale Short Form (GDS-SF) (Onsite, if possible)
- Adverse Event Assessment (In home, by phone or video call, or onsite)
- Dietary assessment and consultation (In home, by phone or video call, or onsite)
- Physical Exam and completion of Ferriman-Gallwey Scale (In home or onsite)

- Mammogram (only women in the EX and EX+T groups) (Onsite)
- Transvaginal Ultrasound (only women in the EX and EX+T groups with a uterus) (Onsite)
- Blood draw (In home or onsite) for the following lab tests:
    - 25-OH Vitamin D
    - Serum Testosterone
    - Serum Estradiol
    - Serum SHBG
    - Complete Metabolic Panel
    - Complete Blood Count
    - Serum Lipids

10.4 Order of Study Procedures

Detailed information regarding ordering of study procedures is described in the Manual of Procedures.

Section 11: Standard Care Procedures for All Participants

11.1 Dietary Assessments

All subjects will undergo a nutritional assessment at baseline by a dietician to: 1) document the subject's caloric intake, primarily focusing on the macronutrient composition of the diet and; 2) evaluate for any recent weight loss. The dietitian will perform a 24-hour diet recall and enter the information into a web-based food record.

The dietician will use the information obtained from the food record, measurements of height and weight to make dietary recommendations consistent with standard of care after hip fracture repair [(98)]. A specific diet will not be prescribed, but rather, participants will be counseled on ways to enhance their protein and caloric intake, as indicated. We will encourage a diet that contains 1.2 gm/kg per day of protein[(99)]. Caloric needs will be estimated using the Mifflin-St. Jeor equation [(100)] and adding an activity correction factor of 1.4.

For any participant requiring supplementation of calories and/or protein intake, the dietary counseling will aim to optimize the participant's ability to implement this on her own.

Participants will be weighed monthly. The dietitian will administer the web-based food record again at month 3 and review the results with the participant. The dietician will work with participants on an individualized basis to adjust dietary intake, as indicated based on measures of body weight and macronutrient intake, as measured by the food diary. The study coordinator will notify the site PI and the dietician regarding any participant who loses greater than 3% of body weight over a month. Loss of 5% body weight between visits will trigger a referral to the dietician for follow-up.

11.2 Vitamin D_3 and Calcium Supplementation

Because of the high prevalence of Vitamin D deficiency in this population[(101)], and to minimize the potentially confounding effect of vitamin D myopathy[(102)], we will provide vitamin D supplementation to all participants enrolled in the study. In the WU hip fracture studies, the 25-OH vitamin D level at baseline was 16 ± 9 ng/mL and in the BHS studies it was 12 ng/mL. 25-OH Vitamin D levels will be measured at baseline and Month 6. At randomization all participants will be provided with Vitamin D_3 2,000 IU to be taken daily. Based on data from Gallagher[(103)], this should be sufficient to increase vitamin D levels into the normal range quickly and maintain them during the study. Participants will also be given calcium carbonate at 1,000 mg/daily in divided doses. Calcium and/or Vitamin D will not be given to those participants with contraindications identified by the site physician at screening.

At monthly assessment points, each site will provide randomized participants a monthly supply of the following:

- 2,000 IU Vitamin D_3, one tablet daily
- 500 mg Calcium, 1 tablet twice daily

Each site will prepare a label and directions for the use of all study supplements that complies with federal and state requirements. Each clinical site will be responsible for maintaining the STEP-HI drug accountability log for the Calcium and Vitamin D_3.

11.3 Physical Therapy Evaluation

All participants will undergo a clinical evaluation at baseline by a site Supervising Physical Therapist. The purpose of this evaluation is to identify the patient's physical limitations/impairments (e.g., ROM limitations, hip precautions, pain, assistive devices, etc.) that may limit their ability to perform specific exercises and/or require modifications. Information obtained from this evaluation will be used by the Supervising PT to provide guidance to exercise interventionists conducting the exercise programs.

Section 12: Randomization

The DCC will create an online password protected randomization system that will be used to facilitate the random assignment of subjects to the three study arms. Site pharmacists will be assigned a unique, nontransferable user ID that will be required to obtain random treatment allocations. The system will be created using the built-in randomization module of the REDCap system. The DCC will randomize participants in REDCap if all eligibility criteria are satisfied. Then the pharmacists will login to obtain the assignment to dispense the appropriate gel bottle if participants are randomized to the EX group.

At the outset of the study, participants were randomized equally into each study group. Following the presentation to the DSMB on April 22, 2020, the sample size was reduced from 300 to 120-168 participants. The numbers in each group will be adjusted as follows:

Randomized Participants	Participants in EX+T	Participants in EX+P	Participants in EUC
300	100	100	100
168	70	70	28
120	50	50	20

The randomization structure was altered to assure that each group would be fulfilled with roughly 1 in 9 participants being assigned to EUC and the other 8 being assigned to an EX group (equally divided between EX and EX+T). To avoid temporal bias, randomization will be blocked within clinical sites using random block sizes in order to preclude the possibility that investigators might know in advance the assignment of the last subject in a particular block.

To ensure that those performing evaluations remain blinded to treatment assignment, randomization assignments will only be viewed by appropriate unblinded site investigational pharmacists who have received training and certification on STEP-HI randomization procedures.

The date of randomization will mark the start of follow-up for each participant; a computer record is maintained for each attempt to randomize a participant. The clinical site coordinator will inform participants of their exercise group assignment. The coordinator will also provide the name of the exercise interventionist who will be contacting them. The coordinator will then alert the assigned exercise interventionist, who will contact the participant to schedule the first intervention visit.

Participants will be assigned to one of three treatment groups for 6 months:

1. **Enhanced Usual Care** (Home exercise + Health Education)***(EUC)***
2. **Exercise** (Placebo gel + Supervised Exercise Training)***(EX)***
3. **Combined** (Testosterone gel + Supervised Exercise training)***(EX+T)***

Subjects in the EX and EX+T groups will be blinded to medication group assignment with testosterone and placebo administered identically. Participants in the two Supervised Exercise Training groups (EX and EX+T) will receive supervised exercise training. The Enhanced Usual Care group (EUC) will receive a low intensity home-based exercise program and monthly health education sessions, but will not apply gel.

Section 13: Enhanced Usual Care (EUC) Group Activities

13.1 Home Exercise Program

Participants assigned to the EUC group will be prescribed an approximate 6-month low intensity home-based exercise program that mimics standard clinical care at the time of discharge from physical therapy after a hip fracture repair. The instructional sessions will be conducted at the clinical site, in your home, or over video call by an exercise interventionist, which is defined as a physical therapist, exercise physiologist, certified personal trainer, or an individual with an appropriate level of experience, as approved by the CCC. EUC participants will be prescribed a standard set of 9 exercises. The participants will be shown how to do the exercises and given written instructions with diagrams illustrating the exercises. Participants will be instructed to perform the exercises 3 times per week, performing each exercise for 10 repetitions. Participants will not be discouraged to perform the exercises more often. The exercise interventionist will meet with the participant monthly to review the exercises, observe their performance, and provide guidance for correct performance and standardized instructions for progressing some of the exercises.

13.1.1 Home Exercise Adherence

All participants will maintain a log of their exercise sessions on a log form. The forms will be turned in to the study coordinator on a monthly basis. Adherence will be measured as the percentage of exercise sessions completed out of a possible 72 sessions.

13.2 Health Education Sessions

Participants in the EUC group will attend a monthly health education session to address health issues unrelated to exercise[104, 105]. Health education sessions will be led by the study coordinator or other appropriately trained study staff, and will cover topics on healthy aging with use of publicly accessible content. The health education sessions will be presented using materials prepared with PowerPoint software. Education sessions will last approximately 30-40 minutes and will ideally be scheduled to coincide with the participant's monthly follow-up visit. The educational sessions may take place in the participants' home, via remote video call, or over the telephone.

Section 14: Supervised Exercise Training Group Interventions

The Supervised Exercise program is whole body, multi-modal program that is conducted in two phases: a light resistance, flexibility, and balance training program (Phase 1, approximately one month), followed by progressive resistance training (Phase 2, approximately five months). Participants will be asked to complete exercise training sessions 2 times per week for 6 months. Each session will last approximately 60 minutes, including rest periods. Priority will be given to onsite training sessions conducted at an exercise training facility and/or outpatient rehabilitation center specified at each of the clinical sites. If after starting the intervention, the participant refuses to attend onsite training sessions due to fear of COVID-19 or in the event the gym is closed due to COVID-19 restrictions, remotely supervised training sessions will be offered. The training sessions will be conducted by an exercise interventionist. Exercise interventionists will be supervised by a site PT and will also be monitored by the lead CCC PT. A clinical site physician will provide medical supervision.

This program will be modified as necessary to accommodate subjects with physical impairments that would make a given exercise difficult, inappropriate, or unsafe. Any modifications will be discussed with the Supervising PT and/or site PI. If possible, the remote supervised exercise sessions will be conducted using secure video calling to be able to demonstrate proper form and visualize the participant completing the exercises. As long as the participant has home wireless internet, additional equipment (such as tablet with camera and stand) can be loaned to assure technology is not a barrier.

Some equipment, such as weight training machines, may vary between clinical sites, and will be approved in advance by the CCC. Equipment used for remote sessions (such as blood pressure cuff, leg weights, or TheraBands) will be provided, if needed.

Because the first consideration in designing the individualized weight-training programs is participant safety and well-being, exercise prescriptions will be individualized and ongoing monitoring during each session will be performed using the modified Borg scale, with the parameters outlined in the Manual of Procedures. In addition, as a safety precaution, blood pressure and heart rate will be measured before and after each intervention session. All exercise interventionists will be familiar with the site institutional policies to provide or activate immediate care when faced with medical emergencies.

During the onsite sessions, the participants will perform all free-weight and machine-based resistance exercises at a target goal of 80% of a 1-repetition maximum. There is no need to perform an actual 1-RM to progress exercise using these criteria. A participant is exercising at 80% of a 1-RM when she can lift a weight 8 times against a given resistance, but is unable to complete the 9th repetition. During remote supervised exercise sessions, participants will be encouraged to complete as many repetitions as possible, since the weights and equipment available may not allow the onsite target of 80% of a 1-RM. The number of repetitions the participant is able to do will help calculate the dose received.

During the first exercise intervention visit, participants will be oriented to the exercise staff, the facility and some of the exercise equipment. The appropriate exercise intensity will be measured after participants have become familiar with the equipment. Each session must start with an active warm up activity (walking, arm circles) and end with an active cool down. The

preferred order of intervention is flexibility exercises, resistance training exercises, and function-based activities.

14.1 Physical Therapy Exercises (Phase 1)

The purpose of the Phase 1 exercise program is to promote improvements in balance, range of motion, muscular endurance, speed of movement, and coordination, and to prepare participants for the machine-based weight-training portion of the intervention. The Phase 1 exercises become progressively more difficult and advanced over the training period.

For a portion of the session, participants may perform individualized exercises designed to correct specific impairments that are detected during the baseline evaluation by the Physical Therapist (such as poor flexibility in a joint not included in the standard protocol that could impact function).

During Phase 1, onsite strengthening exercises will be performed at an 8-RM intensity. A participant is exercising at an 8-RM when she can lift a weight 8 times against a given resistance, but is unable to complete the 9th repetition appropriately. During remote sessions, participants will be encouraged to perform the exercise until they demonstrate failure. If the participant is able to lift the weight more than 8 times, weights will be increased if safe and feasible to do so. Failure is defined as the inability to complete final repetition through full, available range of motion without significant compensation. Signs of compensation are as follows: sudden increases in speed to overcome resistance, improper form, and/or increase in the level of assistance needed.

14.2 Progressive Resistance Training (Phase 2)

Phase 2 consists of five months of progressive resistance training (PRT). This is an essential component of this intervention because high-intensity weight-training programs have demonstrated the greatest effects on increasing muscle strength in older adults. PRT will be performed using weight-lifting machines and free-weights when onsite or free weights, TheraBands, and found objects during remote sessions.

Like Phase 1, Phase 2 onsite strengthening exercises will be performed at an 8-RM intensity. A participant is exercising at an 8-RM when she can lift a weight 8 times against a given resistance, but is unable to complete the 9th repetition appropriately. During remotely supervised exercise sessions, participants will be encouraged to perform the exercise until they demonstrate failure. If the participant is able to lift the weight more than 8 times, weights will be increased if safe and feasible to do so. Failure is defined as the inability to complete final repetition through full, available range of motion without significant compensation. Signs of compensation are as follows: sudden increases in speed to overcome resistance, improper form, and/or increase in the level of assistance needed. Functional resistance exercises should be progressed by increasing difficulty of the task.

Equipment for onsite weight training sessions includes free weight sets (barbells and dumbbells) and weight-lifting machines. Remote training sessions require ankle weights, Therabands, or other objects found around the home to offer appropriate resistance. Equipment is not needed for the functional resistance exercises.

14.3 Remote Supervised Exercise Sessions

Participants who are unable to present to the center due to COVID restrictions, will be asked to perform the supervised exercise sessions remotely through secure video calls with the exercise interventionist. Tablets with cameras and stands will be provided to participants who do not have the equipment necessary to participate in a video call. The stands will ensure the exercise interventionist can view the participant performing the exercises. Prior to the session, the study staff will review the procedures to log on to the video call with the participant. When the video call starts, the exercise interventionist will verify the participant's name and location to dispatch emergency personnel, if needed. The exercise interventionist will assess the environment visible on camera for safety concerns and conduct a symptom check and vital sign measurements with an electronic blood pressure cuff.

During the session, the exercise protocol will be delivered, which closely follows the Phase 1 and Phase 2 onsite exercise sessions, only excluding exercises that are unsafe without assistance or deemed inappropriate for the individual. Participants will be encouraged to perform the exercise until they demonstrate failure. If the participant is able to lift the weight more than 8 times, weights will be increased if safe and feasible to do so. The sessions are recorded by the exercise interventionist on the exercise logs.

14.4 Home Maintenance Exercise Prescription

Participants in the Supervised EX groups will also be given a set of a few functional-based exercises to complete on their own at home about 3 days per week on non-supervised exercise days and encouraged to perform a progressive walking program (for a goal of 10-20 minutes total a day). Participants will record home exercise sessions on a daily log sheet.

14.5 Scheduling Exercise Intervention Visits

General requirements for scheduling of exercise intervention visits:

1. The first exercise intervention visit should occur within 7 days of randomization, but preferably within 3 days.
2. Exercise intervention should occur two times per week, and onsite sessions should never occur on two consecutive days, even when moving from one week to the next.
3. Within the 6-month intervention period, unexpected missed visits can be made up as long as there is at least a day of rest scheduled between each exercise session. If the participant or research staff anticipate missed sessions due to 3-month testing or any other reason, visits can be made up the week prior as well.
4. During weeks with a study follow-up visit, exercise intervention visits may occur on the same day if the follow-up visit does not include any physical performance measures and provided that follow-up activities are completed first.
5. If a participant misses 2 consecutive exercise sessions or more than 2 visits in any month that are not made up, the site coordinator will be notified.

14.5.1 Missed Exercise Sessions

Participants who miss an exercise session (or anticipate missing one) will be allowed to make it up, preferably within the same (Monday-Friday) week, but could also be allowed to add a session any time prior to the 6 month assessment. As with scheduling exercise intervention visits in general, a make-up session may not be scheduled on a day preceding or following a scheduled onsite exercise session. The site coordinator must be notified of any participant who

misses 2 consecutive sessions or more than two sessions in a month, to consider what can be done to reduce missed sessions.

14.6 Adherence Measurements

Adherence to the supervised exercise program will be measured as the percentage of exercise sessions successfully completed out of a possible 48 sessions. We define a successfully completed exercise session as one in which 70% or more of the exercises are performed at the target number of repetitions. Exercise interventionists will complete a checklist and exercise log each visit documenting the number of sets, repetitions, weight used or other specifications of the exercise. If the participant was able to complete at least 5 repetitions, the set will be considered complete. Adherence to the home maintenance exercises will be measured with log forms similar to the EUC group.

14.7 Fidelity Plan for Exercise Interventions

Treatment fidelity[106, 107] for the interventions will be evaluated with regard to: (1) design, which focuses on whether the interventions are consistent with underlying theories, and whether the study is free of contamination such as treatment crossover or unintended motivational interventions; (2) training, which addresses skill acquisition and maintenance for PT/exercise interventionists; (3) delivery, or the assessment that the interventions were implemented as intended by study coordinators, dieticians, and physical therapists/exercise interventionists; (4) receipt, which focuses on whether or not the participant understood and received the interventions as intended. Overall, treatment fidelity data will provide information on the adherence of the exercise interventionists to the interventions, and the adherence of the participants to the prescribed activities. Monitoring treatment fidelity also will provide an opportunity to address potential study problems, such as drift from the intervention protocols that could threaten the study's ability to detect treatment differences. The Clinical Coordinating Center (CCC) will be responsible for oversight of treatment fidelity procedures.

14.7.1 Initial Training and Certification for Procedural Reliability

The Clinical Coordinating Center (CCC) Lead Physical Therapist and staff will serve as Intervention Monitors (IMs) and will train all clinical site staff in the onsite supervised exercise, remote supervised exercise, and home exercise intervention procedures. Knowledge of procedures will be tested by written examination. Following the written examination, the clinical site Supervising Physical Therapist will observe site exercise interventionists conducting at least one Phase 1 onsite session and at least one Phase 2 onsite supervised exercise session with a volunteer (mock) participant using standardized checklists. The sessions will also be video recorded. The structured checklist assesses data completeness, physical performance, qualitative observations, and verbal and non-verbal communication. The checklist will also document whether the participant completed the warm up exercises, necessary repetitions at the appropriate intensity and cool down exercises. If the exercise interventionist scores 90% or higher, the video file will be sent to the CCC PT/IM for review. If the score is less than 90%, the Supervising PT will proceed with retraining and remediation until the exercise interventionist scores 90% or higher on the fidelity checklist.

The CCC PT/IMs will review the Phase 1 and Phase 2 exercise session videos using the fidelity checklists. If the score is 90% or higher, the exercise interventionist is considered "STEP-HI certified" and the CCC will issue a certificate of completion. When a new staff member joins the

project, training will include viewing of a required training video and the procedures described above.

14.7.2 Ensuring Ongoing Competency

We will use a multi-faceted approach to ensure ongoing treatment fidelity. The approaches include direct observations by the site Supervising PT and CCC Lead PT, periodic review of exercise logs, mandatory monthly conference calls lead by the CCC Physical Therapist with site Supervising PTs and exercise interventionists, and face-to-face meetings with the intervention team when indicated for remediation that cannot be managed by other means.

14.7.2.1 Direct Observation of Exercise Procedures

The Supervising PT will directly observe exercise interventionists during onsite exercise training sessions or remote sessions, and document their observations using standardized checklists. Each exercise interventionist will be directly observed by the Supervising PT during the training of the first two or three participants; at least two sessions during Phase 1 and two sessions during Phase 2. The Supervising PT will use the checklist and their observations to provide specific feedback to the exercise interventionists about their implementation of the exercise protocol, any specified modifications, and other issues such as verbal and non-verbal communication with participants.

Site Supervising PTs will be responsible for observing exercise interventionists on a monthly basis, using the standardized checklists. Approximately every six months, a video recording of one Phase 1 exercise session or one Phase 2 session will also be sent to the CCC Lead PT to ensure appropriate ongoing fidelity to the exercise protocol. The CCC Lead PT will review video recordings using the standardized checklists. If the score on the checklist is less than 90%, the clinical site PI and site Supervising PT will be notified. A remediation plan will be proposed that will offer refresher training to ensure accurate understanding of the protocol, follow-up observation visits, and possible dismissal if warranted.

14.7.2.2 Review of Intervention Logs

The clinical site exercise interventionists will review participant home exercise logs monthly, either on site or electronically in REDCap. The clinical site study coordinator, in collaboration with the DCC, will monitor whether the supervised exercise intervention logs are complete for all sessions, whether exercises are recorded as complete, and whether any modifications to the intervention protocol are appropriately documented. The progression of the supervised exercise program will also be monitored by the site Supervising PT, and by the CCC Lead PT. This information will be available to the CCC PT for discussion with clinical site staff directly, and/or during monthly conference calls. Additional follow-up will be mandated if problems with administration of the interventions are identified. A remediation plan will developed and monitored if there is ongoing lack of progress.

14.7.2.3 Mandatory Conference Calls with PTs and Exercise Interventionists

The CCC IM/Lead PT will conduct monthly telephone calls with PTs and exercise interventionists. Minutes of the telephone meetings will be recorded. During these calls, the CCC PT will ask questions to identify problems with delivering the intervention, provide guidance, and assist with problem solving. If problems are identified, the CCC Lead PT may schedule an individual site telephone call and review of exercise logs.

Section 15: Testosterone/Placebo Intervention

15.1 Drug Distribution

Testosterone therapy will be provided in the form of a topical gel (generic 1.0% testosterone, like those manufactured by Perrigo or Activas/Teva). Topical T gel will be used because it is documented to provide continuous transdermal delivery of T for 24 hrs.[108-110]. The advantage of topical therapy over oral or intramuscular preparations is that it is easier to achieve and maintain stable physiologic levels of T, particularly in women, and is safer. The potential advantages of topical gel over a transdermal patch include ease of delivery and dose titration, better skin tolerability, and invisibility after application[33].

Placebo will be provided as an identical but inactive gel (hand sanitizer Purell™ or an equivalent generic) in identical bottles.

The CCC will arrange for both the active and inactive gels to be shipped to the Clinical Site Investigational Pharmacies. The investigational pharmacy at each clinical site will be responsible for receiving, storing, and distributing the testosterone and placebo bottles. Detailed logs showing the receipt, distribution, and destruction of the investigation drug will be kept. All pump bottles will be weighed by the local Clinical Site Investigational Pharmacist at the time that the bottle is dispensed. This information will be recorded and maintained by the Clinical Site Investigational Pharmacist. An Investigational Drug Accountability Log entry will be completed by the Clinical Site Investigational Pharmacist each time a gel bottle is received, dispensed, or destroyed. All expired, unused, or empty containers at the clinical centers can be disposed of per the institution's standard drug destruction procedure. Written documentation of what bottles were discarded, and the date is mandatory. The gel bottles must be stored at 20 to 25°C (68 to 77°F). Excursions between 15°C and 30°C (59°F and 86°F) that are experienced in pharmacies, hospitals, and warehouses, and during shipping are allowed. Provided the mean kinetic temperature does not exceed 25°C, transient spikes up to 40°C are permitted as long as they do not exceed 24 hours.

15.2 Dosing and Schedule

Women assigned to the active treatment group (EX+T) will be prescribed a dose of T gel that is anticipated to achieve a supra-physiologic serum T level (target T level 110 -160 ng/dl; reference range 12 - 78 ng/dl). The initial dosage of T gel will be 12.5 mg (1 pump depression per day of 1% testosterone gel) to achieve total testosterone levels in the target range. T-levels will be measured at 2 weeks, 1 month, and then monthly. Women with serum testosterone levels below the target range will have their dose increased by 12.5 mg (1 pump depression) increments, to a maximum of 37.5 mg (3 pump depressions). Women with levels above the target range will have their dose reduced to 12.5 mg (1 pump depression) every <u>other</u> day. Subsequent dose adjustments will be individualized by the CCC unblinded physician.

Dispensing of the first month's supply of study drug or placebo will occur on the day of randomization. Subsequent refills will occur at study follow-up visits or before, if needed.

Participants will meet with the study coordinator at the randomization visit to receive detailed oral and written instructions regarding the proper application of the gel. The study coordinator will observe the patient self-apply the first dose of gel. Participants will also be educated about

the precautions that should be taken to prevent other individuals from being exposed to the gel. These include washing hands with soap and water immediately after applying the gel and covering the application site with clothing after the gel has dried. Additionally, the application site should be washed thoroughly with soap and water prior to skin-to-skin contact with another person, which should be several hours after gel application.

15.3 Dose Adjustments

Peak serum total testosterone levels will be measured at 2 weeks post randomization and at the monthly visits. Doses will be adjusted by an un-blinded study physician in the CCC who is not involved in screening, baseline or follow-up testing. On the day of the follow-up visit, the participant will be instructed not to apply the gel until their appointment at the clinical research facility. The participant will apply the gel under the direct supervision of the study coordinator. A blood sample for peak testosterone levels will be obtained 2 hours after gel application. Testosterone levels will be reported only to the unblinded CCC physician. To maintain blinding, the unblinded CCC physician will direct dose adjustments and a member of the CCC or DCC will communicate changes in prescriptions to the clinical site coordinator. The CCC physician will direct a subject in the placebo (EX) group to be treated similarly to an active treatment (EX+T group) subject (e.g., have the gel dosage increased or decreased by one pump depression). Individuals with mild symptoms or signs of virilization who wish to continue in the study will have the dosage reduced. For the active T-gel participants we will aim to maintain the level within the bounds of the target range (110-160 ng/dl), but if this is not tolerated the dosage will be adjusted to the highest tolerable dosage.

15.3.1 Adverse Events and Symptom Monitoring

Adverse events (AEs) will be solicited on a monthly basis and documented if a participant volunteers an event to a study staff member. The study coordinator will review adverse events with the participants if additional information is needed and document the AE. Study staff will notify the site physician of any concerning symptoms as soon as possible. Women assigned to either the EX+P or EX+T groups who experience an adverse event due to an acute cardiovascular event such as a TIA, CVA, or cardiac ischemia will have their assigned gel treatment discontinued, but can continue other study interventions and procedures unless instructed not to by their health care providers.

15.3.2 Medication Adherence

Participants will maintain a daily log in which they will record the location on the skin and the time of day the gel was applied each day. The study coordinator will telephone the subject weekly during the first month to discuss any questions that arise in relation to gel application.

At monthly follow-up visits the pump bottle dispensed previously will be returned to the investigational pharmacy, documented, and weighed. For women prescribed a dose of less than one pump depression per month, the bottle may be returned after two months. The study coordinator will document the prescribed dosage/amount (number of pumps) and frequency that the subject is administering the gel. The investigational pharmacist will be responsible for weighing the returned pump bottle and completing any required regulatory documentation.

Adherence to the medication will be measured using bottle weights, serum testosterone levels, and monthly log reviews. Based on the prescribed dosage, adherence will be measured as the

percentage of the expected gel bottle weight. Adherence will also be measured as the percentage of testosterone levels within the target range over the 24 week treatment period, and the percentage of reported vs. expected gel applications.

15.3.3 Fidelity Plan for Study Drug Procedures

Fidelity to the testosterone and placebo procedures will be monitored through blinded reporting of bottle weight and testosterone level by clinical site. If bottle weights indicate consistent adherence of < 90% or sub-therapeutic testosterone levels in the active treatment group then the IM will conduct a site visit to review procedures with the site study coordinator. The DCC will generate these monthly reports for the CCC.

Section 16: Safety Considerations

16.1 Pre-Intervention Safety Screening

Potential participants will be excluded during screening if they have cardiovascular diseases or other conditions that would make it unsafe for them to participate in one or both of the study interventions. Information about these conditions will be obtained through chart review, interviews, and consultation with a medical professional familiar with the potential participant (e.g., primary care provider, orthopedic surgeon). The clinical site physician and PI will be responsible for giving permission for potential participants to be randomized, based on thorough review of all eligibility information.

16.2 Safety Considerations for Study Assessments

All study assessments will be done by certified staff, trained to perform the tests safely. If, during any physical testing or evaluations, a participant reports chest pain, tightness or pressure, significant shortness of breath or difficulty breathing, or feeling faint, lightheaded, or dizzy, the test will be stopped. All research staff who administer 1-RM testing or exercise procedures will be familiar with the site policies to provide or activate immediate care when faced with medical emergencies.

16.3 Safety Considerations for Testosterone Therapy

Participants will be rigorously monitored for the development of side effects and potential health problems. Participants will be monitored for signs of hirsutism, endometrial hyperplasia, changes in hematocrit, serum liver function tests and lipids, and cardiovascular outcomes.

16.3.1 Evaluation of Hirsutism (Ferriman-Gallwey scale)

The clinical site PI, a designated physician, or an Advanced Practice provider will complete a physical exam to assess for hirsutism using the Ferriman-Gallwey scale at baseline, month 3, and month 6.

16.3.2 Adverse Event Assessment

At baseline and at each monthly follow-up visit, study staff will ask participants about and document any new or worsening symptoms. Study staff will review symptoms with the clinical site PI and report all symptoms that are considered AEs as directed in Section 17.

16.3.3 Laboratory Testing

Screening laboratory testing will include a complete metabolic panel (CMP), liver function (with complete metabolic panel), complete blood count (CBC), TSH, HgbA1c, and testosterone levels (serum free and total). Baseline laboratory testing will include other hormone levels (serum estradiol and SHBG), Serum 25-OH vitamin D level, and cholesterol levels (Total, LDL and HDL). All of these laboratory tests (with the exception of TSH and HgbA1c) will be repeated on all participants at month 6. In the interim, all participants will have a CMP, CBC, and total testosterone level measured at the month 3 visit. Testosterone levels will be measured in the EX and EX+T groups at week 2, 2 weeks later (month 1), and then monthly. LFT results will be reviewed by the Site Physician or a designee within 3 days, but preferably sooner, of the date that the test is reported. Following screening, testosterone levels and CBC results will be reviewed by the unblinded CCC physician within 3 days, but preferably sooner, of the date that

the test result is reported. The unblinded CCC physician will instruct each clinical site coordinator to make dose adjustments as necessary.

16.3.4 Transvaginal Ultrasound

Women in the EX and EX+T groups with a uterus will undergo a transvaginal ultrasound after completion of the interventions at 6 months. Women who develop abnormal or unexpected vaginal bleeding over the course of the intervention will also be asked to undergo a vaginal ultrasound. Ultrasounds will be read locally by a designated physician. The clinical site PI will review the results and document this in the participant record. Women who have significant endometrial hypertrophy, defined as an endometrial wall thickness > 4 mm will be referred to their gynecologist or primary care physician for follow-up.

16.3.5 Mammogram

Mammograms will be performed on all participants at baseline (if they haven't had one within the past 6 months) Women in the EX and EX+T groups will undergo an additional mammogram at the end of the 6-month intervention period. Mammograms will be read locally by a designated physician. The clinical site PI will review the results and document this in the participant record. Abnormal test results will be discussed with the participant's primary care physician.

16.4 Safety Considerations for Supervised Resistance Training Program

There is no expectation that the EX Program will evoke serious cardiovascular responses; however, participants will be warned of a possible risk. Cardiac events are rare, with estimates of one event per 60,000 participant-hours in aerobic exercise programs[(111)]. No significant cardiac events were reported after performing 1-RM testing for over 6600 healthy subjects[(112)]. The American Heart Association's guidelines for resistance training for adults with and without cardiovascular disease reports the safety of high intensity resistance training and testing in persons with coronary disease which found an absence of anginal symptoms, ischemic ST-segment depression, abnormal hemodynamics, complex ventricular dysrhythmias, and cardiovascular complications[(113, 114)]. Less serious risks may include chest pain, fainting, hypotension, or muscle strain. We have minimized risk to participants by following the guidelines suggested by Gill et al.[(115)] Blood pressure and heart rate will be monitored using a standard blood pressure cuff and palpation of peripheral pulse.

Another concern is the presence of osteoporosis and the risk of inducing a compression fracture or a lower extremity fracture. The exercises have been designed to minimize this risk. The risk of inducing a compression fracture will be minimized because the exercises are performed in supine, sitting, and upright standing positions with minimal to no trunk flexion[(116)]. The PT will also remind the participant to minimize flexion to the spine during all standing exercises. Delayed onset muscle soreness is a common occurrence after the initiation of an exercise program. The soreness occurs in the muscle belly 1-3 days after the initiation of exercise and lasts 2-3 days. There is no effective way to eliminate the risk of delayed onset muscle soreness, but it is hoped that the orientation process and gradual increase in intensity will reduce the risk. The participants will be informed about the condition, what it feels like, how long it lasts, and suggested ways of decreasing the pain including the use of superficial heat or ice. If a participant reports continued discomfort, the site physician will discuss this with the participant and contact their primary care provider (PCP) as necessary.

16.5 Safety Considerations for Remote Exercise Program

The remotely supervised exercise sessions will utilize precautions similar to the onsite supervised program. Participants will monitor their blood pressure and heart rate before and after each exercise session. Exercise interventionists will monitor the participant for proper form during the sessions and will instruct participants in the proper use of equipment. If an adverse event occurs during the session, the exercise interventionist will report back to the coordinator or PI and in severe circumstances, will call on emergency services.

16.6 Safety Considerations for Home Exercise Program

The exercises in the EUC program are similar to those prescribed by physical therapists at the end of standard treatment for a hip fracture and therefore are similar to "usual care" for community-dwelling hip fracture patients, except that subjects enrolled in the study are monitored on a monthly basis in person and will receive weekly contact for encouragement. Participants will be instructed in the proper execution of all assigned exercises by certified staff.

16.7 Participant Education about Potential Risks

Potential risks associated with study-related activities and interventions will be explained to each participant by trained study personnel during the informed consent process and during study orientation. Each participant will be instructed to report the occurrence of a new symptom, medication, or health event to appropriate study staff at scheduled data collection times, to PTs or exercise interventionists administering the intervention, or spontaneously at any other time. Participants also will be encouraged to report concerns about the safety of participating in the study.

Section 17: Adverse Event Reporting

Each site PI has primary responsibility for the safety of participants at his/her clinical site. An Internal Safety Monitor (**ISM**) will review decisions made by site PIs and the categorization of events. In addition, an Independent Data and Safety Monitoring Board (**DSMB**) will monitor the study safety and any potential treatment-related adverse events (AEs), serious adverse events (SAEs), and reportable adverse events (RAEs) to protect the safety of study participants.

Many definitions used in this section are published in the January 2007 OHRP *Guidance on Reviewing and Reporting Unanticipated Problems Involving Risks to Subjects or Others and Adverse Events, OHRP Guidance* and can be found at: http://www.hhs.gov/ohrp/policy/advevntguid.html.

17.1 Definitions

Adverse Events (AEs)

An adverse event (AE) is any unfavorable or untoward medical occurrence in a human study participant, including any abnormal sign (e.g., abnormal physical exam or laboratory finding), symptom, or disease, temporally associated with the participant's involvement in the research, whether or not considered related to participation in the research.

An AE includes any abnormal sign such as abnormal findings from routine exam or laboratory tests, or any disease condition that is present during the participant's involvement in the research. Some examples of adverse events include:

- Any new and pre-existing symptoms or conditions that have become worse either in frequency or severity or both, even if the event was not caused by the study treatment or procedure.
- Any recurrence of previously resolved condition whether or not it is related to the study treatment or procedure.
- Any clinically significant findings from the study-related or non-study related procedures such as ECG findings, laboratory findings, vital sign measurements, physical exams and any other procedures.

Serious Adverse Events (SAEs)

An AE is considered "serious" if it results in any of the following outcomes:

- Death
- Is life threatening, or places the participant at immediate risk of death (e.g., MI, stroke/TIA)
- Requires or prolongs hospitalization
- Causes significant disability or incapacity (e.g., torn muscle or ligament)
- Requires medical or surgical intervention to prevent significant disability (e.g., hip fracture)

Unanticipated Problems (UPs)

Unanticipated problems include any incident, experience, or outcome that meets **all of the following** criteria:

1. Unexpected, in terms of nature, severity, or frequency, given (a) the research procedures that are described in the protocol-related documents, such as the IRB-approved research protocol and informed consent document; and (b) the characteristics of the study population;

2. Related or possibly related to participation in the research (possibly related means there is a reasonable possibility that the incident, experience, or outcome may have been caused by the procedures involved in the research); and
3. Suggests that the research places participants or others at a greater risk of harm (including physical, psychological, economic, or social harm) than was previously known or recognized. (Note per OHRO guidelines, any AE that is serious (i.e. a SAE), unexpected, and related or possibly related to participation in the research automatically meets this third criterion.

Reportable Adverse Events (RAEs)

RAEs are events that have potential implications for participant safety and require individual reporting to the CCC. If the event falls into **at least one** of the following categories it will be considered an RAE:

- Serious Adverse Events (SAEs)
- Unanticipated Problems (UPs)

Adverse Event Reporting Period

Prior to randomization, only adverse events that are a direct result of study procedures or occur while the participant is under study staff supervision will be documented. From randomization until 30 days after the participant's final assessment visit, all AEs (regardless of relatedness) will be documented.

Preexisting Condition

A preexisting condition is one that is present at the start of the study. A preexisting condition should be recorded as an adverse event if the frequency, intensity, or the character of the condition worsens during the study period.

General Physical Examination Findings

At screening, any clinically significant abnormality should be recorded as a preexisting condition, even if it occurs intermittently. At the final study visit, any new clinically significant findings/abnormalities that meet the definition of an adverse event must also be documented.

Post-study Adverse Event

All unresolved adverse events should be followed by the site PI until the events are resolved, the subject is lost to follow-up, or the adverse event is otherwise explained. At the last scheduled visit, the participant should be instructed to report any subsequent event(s) that the participant, or the participant's physician, believe might be reasonably related to participation in the study. Sites should document any death or adverse event occurring at any time after a participant has discontinued or withdrawn from study participation that may reasonably be related to the study.

Abnormal Laboratory, Vital Signs, or Electrocardiogram Results

A clinical laboratory abnormality or abnormal objective test finding should be reported as an AE if **any one** of the following conditions is met:

- The test result is associated with accompanying symptoms;
- The test result requires additional diagnostic testing or medical surgical intervention; OR
- The test result leads to a change in dosing (outside of protocol-stipulated dose adjustments), discontinuation from the study, significant additional concomitant drug treatment, or other therapy.

Repeating an abnormal test, in the absence of any of the above conditions, does not constitute an AE. Any abnormal test result that is determined to be an error does not require reporting as an AE.

Surgery, Hospitalization, or Prolonged Hospitalization
Any adverse event that results in surgery, hospitalization, or prolonged hospitalization should be documented and reported as a serious adverse event unless:

- The hospitalization or prolonged hospitalization is for diagnostic or elective surgical procedures for a preexisting condition. Surgery should not be reported as an outcome of an adverse event if the purpose of the surgery was elective or diagnostic and the outcome was uneventful.
- The hospitalization is for respite care
- The hospitalization is planned or for routine maintenance of a device in place before informed consent and the outcome was uneventful.

17.2 Categorizing an Adverse Event

17.2.1 Expectedness

Each AE should be evaluated as to whether it is expected or unexpected.

Expected Adverse Events
An AE can be deemed as expected if it can be:

- Anticipated on the basis of prior experience with the intervention under investigation;
- Attributed to the underlying condition of the participant being studied; OR
- Attributed to the patient population being studied.

The following are expected adverse events that will be listed in the ICF:

MEDICATIONS
Testosterone Gel:

- mild edema in legs
- facial acne
- hair growth
- scalp hair loss
- enlarged clitoris
- change in blood lipids
- uterus lining thickening
- vaginal bleeding
- allergic reaction characterized by:
    - rash
    - shortness of breath
    - wheezing
    - difficulty breathing
    - sudden drop in blood pressure
    - swelling around the mouth, throat, or eye
    - fast pulse
    - sweating

Calcium Supplements:

- constipation

Vitamin D Supplements:

- elevated calcium level in blood

- kidney stones
- frequent urination
- irregular heart beat

STRENGTH TESTING, PHYSICAL ASSESSMENTS, and EXERCISE SESSIONS:

- muscle soreness
- joint stiffness
- foot, knee, hip, or back pain
- fatigue
- pulled muscles
- heart problems (following exercise)
- fall (with or without injury)
- increased heart rate
- chest pain
- shortness of breath
- headache
- nausea

SAFETY ASSESSMENTS

Blood Draws:

- bleeding
- bruising
- pain
- dizziness
- fainting
- infection

ECG:

- skin redness at patch site
- skin itching at patch site
- skin discomfort at patch site
- hair loss with patch removal

Mammogram:

- discomfort

Transvaginal Ultrasound:

- discomfort

Unexpected Adverse Events

An AE is considered unexpected if the event:

- Is not anticipated on the basis of information associated with resistance training in the elderly, the investigational drug package insert, or the current IRB approved consent.
- Cannot be attributed to the underlying disease, disorder, or condition of the participant being studied or to the patient population; OR
- Exceeds the frequency or severity anticipated.

17.2.2 Relatedness

The potential relationship of the event to the study intervention and/or participation is assessed by the site PI and/or study clinician and reviewed by the ISM. The categories are:

- Definitely Related: The adverse event is clearly related to the investigational agent/procedure – i.e., an event that:
  - follows a reasonable temporal sequence from administration of the study intervention,
  - follows a known or expected response pattern to the suspected intervention,
  - is confirmed by improvement on stopping and reappearance of the event on repeated exposure and
  - could not be reasonably explained by the known characteristics of the subject's clinical state.
- Possibly Related*:* An adverse event that follows a reasonable temporal sequence from administration of the study intervention, follows a known or expected response pattern to the suspected intervention, but that could readily have been produced by a number of other factors.
- Not Related*:* An adverse event clearly unrelated to the investigational agent/procedure - i.e., another cause of the event is most plausible; and/or a clinically plausible temporal sequence is inconsistent with the onset of the event and the study intervention; and/or a causal relationship is considered biologically implausible.

17.2.3 Severity

The following guidelines will be used to rate the severity of RAEs:

- Mild: Participant is aware of signs or symptoms that are transient in nature, easily tolerated; do not require therapy or medical evaluation and are only minor irritants that cause no loss of time from normal activities.
- Moderate: Events introduce a low level of inconvenience or concern to the participant and may interfere with daily activities. Symptoms may require minimal, local, or noninvasive medical intervention only.
- Severe: Events interrupt the participant's normal daily activities and are usually incapacitating. Significant symptoms may require hospitalization or invasive medical intervention.
- Life threatening: Events that may involve acute, life-threatening metabolic or cardiovascular complications (such as circulatory failure, hemorrhage, sepsis) or life-threatening physiological consequences. Intensive care or emergent invasive procedure is required to prevent death or disability.
- Fatal: Events result in death.

Severity is not synonymous with seriousness. A severe headache may not necessarily have a severity rating of "severe". However, mild chest pain may result in a day's hospitalization and be classified with a rating of "severe".

17.3 Adverse Event Recording

At each monthly visit, study staff will seek information on adverse events by specific questioning and, as needed, by examination. If, at an intervention visit or during a phone conversation, participants volunteer that an event occurred, adverse events will be recorded by study staff. All adverse events should be reported on the case report form (CRF) and the appropriate source

documentation should be filed in the participant's binder. All clearly related signs, symptoms, and abnormal diagnostic procedure results should be recorded, but should be grouped under one diagnosis.

All adverse events occurring during the adverse event reporting period must be recorded. The clinical course of each event should be followed until resolution. Serious adverse events that are still ongoing at the end of the adverse event reporting period must be followed up to determine the final outcome. Any serious adverse event that occurs after the adverse event reporting period and is considered to be possibly related to study participation should be recorded and reported immediately.

17.4 Adverse Event Reporting

Clinical site PIs will be responsible for reviewing AEs and assuring accurate and timely reporting of RAEs. Clinical sites will be responsible for reporting RAEs to their respective IRBs and risk management offices according to local requirements. The clinical site physician or designee will be available for consultation and participant evaluation (if needed) during time periods when participants are engaged in interventions or clinical assessments. The clinical site PI will also review RAEs recorded at the site. The CCC will report AEs to the appropriate groups and agencies within the required timeframe as defined in the Manual of Procedures. The CCC will also communicate information back to the sites and make changes to the ICF as requested by the monitoring groups and agencies.

17.5 Emergency Unblinding

The unblinding of treatment assignment for a subject may be necessary due to a medical emergency or any other significant medical event. Subject safety must always be the first consideration in making such a decision. The site PI should make every effort to contact the Study Chair prior to unblinding to determine whether it is necessary. If unblinding is deemed crucial, the site PI will contact the site investigational pharmacist or the unblinded CCC physician for the treatment group assignment. The site PI will detail the reason for the decision and include appropriate source documents. The investigator will inform the CCC coordinator that the subject has been unblinded. If an investigator is unblinded, the participant may remain in the study after obtaining approval from the Study Chair.

17.6 Adverse Event Monitoring

17.6.1 CCC Monitoring

The CCC, with the help of DCC reports, will monitor site recording and reporting procedures and unresolved AEs at site visits and remotely as described in Section 21.5: Reportable Adverse Event (RAE) Reporting.

17.6.2 Internal Safety Monitor (ISM)

CCC physicians who are not affiliated with conducting the study will serve as ISMs to review the final status of RAEs reported to the CCC on a rotating basis. After a RAE is finalized by a clinical site and reported to the CCC, it will then be forwarded to the on-call ISM. The ISM will be responsible for:

- Confirming or refuting the final classification of an event as an RAE;
- Confirming or refuting the classification of expectedness of the RAE;

- Confirming or refuting the classification of relatedness to the study intervention and/or participation of the RAE;
- Confirming or refuting the classification of severity of the RAE; and
- Recommending changes to the protocol or consent form, if he/she believes changes are warranted.

17.6.3 Independent Data and Safety Monitoring Board (DSMB)

The DSMB will receive safety reports and meet roughly every 6 months to monitor study safety and progress. They will make recommendations regarding study initiation, continuation, and protocol changes. The DSMB may meet at any time needed to review the study relevant information, such as occurrence of RAEs, scientific or therapeutic developments that may have an impact on the safety of the participants or the ethics of the trial. The DSMB will make recommendations to the NIA and the cPI, concerning continuation, termination, or other modifications to the study based on the observed beneficial or adverse effects of the treatment under study. Because the DSMB may be required to review unblinded analyses, their names and contact information are not included in this document and will not be made available to the study staff members.

Section 18: Discontinuation

Discontinuation as used herein refers to (1) the discontinuation of the treatment in particular individuals because of adverse events; (2) the discontinuation of the study because of adverse events, demonstrated efficacy, or futility; and (3) the discontinuation of a single arm of the study because of adverse events, or demonstrated futility associated with the particular study arm.

Certain RAEs may result in a temporary interruption or early discontinuation of trial assessments and interventions or components of these assessments and interventions in an individual participant. After such RAEs occur, a participant may resume the trial intervention when the clinical site physician and the primary care provider (if participant has one) agree that it is appropriate. Women assigned to either the EX+P or EX+T groups who experience an adverse event due to an acute cardiovascular event such as a TIA, CVA, or cardiac ischemia will have their assigned gel treatment discontinued, but can continue other study interventions and procedures unless instructed not to by their health care providers. For mild problems that require temporary cessation of intervention, the clinical site PI, in consultation with the clinical site physician and participant, will confirm that the participant can resume the study intervention.

At any time, the DSMB may recommend discontinuation of any component or intervention group of the study for any of the following reasons:

1) **Adverse events:** Compelling evidence from this or any other study of an adverse effect of the study intervention(s) that is sufficient to override any potential benefit of the interventions to the target population.
2) **Demonstrated efficacy:** Compelling evidence from this or any other study of a significant beneficial effect of the study intervention(s), such that its continued denial to other study group(s) would be unethical. The possibility of an interim futility analysis will be discussed with the DSMB.
3) **Demonstrated futility:** A very low probability of addressing the study goals within a feasible time frame.

The site PI may recommend discontinuation of all components of the intervention in a given participant for the following reasons:

1) Participant becomes lost to follow-up.
2) To protect a patient from excessive risk, as per the safety alert and action tables (See MOP).
3) To maintain study integrity if the participant is found to be untruthful.
4) Participant has a change in cognitive status causing safety concern.

Procedures for assessment of excessive risk or change in cognitive status are discussed in more detail in the MOP.

The participant may also withdraw voluntarily from participation in the study at any time and for any reason. If the participant decides to withdraw from intervention participation, she will be encouraged to continue in the study for safety monitoring and outcome assessments at the specified time points in the protocol. If she declines, we will request that she return to the research center for a final early withdrawal visit. Prioritized outcome measures will be performed at this study withdrawal visit allowing the participant to stop at any point.

Participants who choose to withdraw early or are discontinued by the study will not be replaced.

18.1 Study Drug Discontinuation

Subjects who permanently discontinue study medication and who do not withdraw from the study will be asked to return to the clinic for all remaining study visits per the visit schedule. Particular emphasis will be placed on encouraging participants who discontinue study medication to return for the final study visit.

18.2 Study Exercise Intervention Discontinuation

Participants who permanently discontinue the weekly exercise interventions and do not withdraw from the study will be asked to continue use of the gel and to return to the clinic for all remaining study visits per the visit schedule.

Participants who discontinue any part of the home exercise program or no longer wish to attend the monthly health education classes, will no longer be contacted weekly. They will still be asked to participate in the assessment time points if they are willing.

18.3 Early Withdrawal Visit

Participants who are not willing to continue with the assessment schedule will be asked to complete the 6 month visit at 6 months post randomization. If the participant is unwilling to wait until the 6 month time, an abbreviated withdrawal visit will be requested as detailed in the MOP, no matter what assessment time point is due or is coming due. The participant is able to refuse any assessment she doesn’t want to complete.

Section 19: Sample Size and Statistical Power

a. Original Sample Size and Statistical Power (until 6/24/2020)

Power computations are based on two-sided tests at the 0.05 level of significance. They focus on 6MWD, the primary outcome measure. The null hypothesis that reflects the primary goal of this study is that the change from baseline to six months in the EX group in 6MWD equals the corresponding change in the EX+T group.

We do not have preliminary data in females from which to directly estimate the magnitude of the projected differences from adding testosterone to the supervised exercise program. However, we do have such data from a randomized, controlled pilot study conducted at WU using the same exercise procedures involving 25 frail hypogonadal (T level <350 ng/dL) males ≥65 yrs. within 6-16 weeks of recent hip surgery. In that study, EX was associated with a 6-month increase in 6MWD of +42.7 ± 69.2 meters, as compared to +82.2 ± 92.5 meters in the EX+T group. If we assume that the benefit of adding testosterone to EX in female hip fracture patients will be of similar magnitude as the observed benefit in the male study, the sample size requirements are 68 per group for a power of 0.8, and 91 per group for a power of 0.9. After accounting for a 20% dropout rate, these sample sizes increase to 85 and 114, respectively. Our plan to randomize 100 subjects per group provides statistical power of 0.86, for an alpha level of 0.05.

The power computations described above are conservative for two important reasons. First, they are based on 80 subjects per group. This number reflects the assumption that no follow-up data will be available on 20% of the 100 who are randomized into each group and whom we assume will drop out before 6 months. But many of the assumed dropouts will not leave the study until after they have provided 3-month data. The availability of those additional 3-month data combined with the imputation procedures we will employ with respect to the missing 6-month data in these subjects means that power will be greater than what we have calculated when we employ our mixed model analyses using intention-to-treat. Second, all dropouts will be strongly encouraged to provide 6-month primary endpoint data. While some will be unwilling to do so, our experience has been that many will be happy to contribute in this limited way to the scientific goals of the study. This will further enhance statistical power. An added point is that we have computed power for the key comparison between EX and EX+T. However, to the extent that EX is superior to EUC, the power for the comparison between EUC and EX+T will be substantially greater than the 0.86 value calculated above.

Although we believe that a sample size of 300 subjects will be sufficient for the primary analysis, we will discuss the possibility of an interim futility analysis with the Data Monitoring Safety Board (DSMB) prior to initiating the study, to guard against the possibility of wasting resources unnecessarily.

b. Revised Sample Size and Statistical Power (starting 6/24/2020)

The primary goal of this study, as reflected in the specific aims, is to compare the change from baseline to six months in six minute walk distance (6MWD) in the exercise (EX) group with the corresponding change in the exercise plus testosterone (EX+T) group. Prior data from a previous pilot study led us to base those computations on projected changes in 6MWD of 42.7 ± 69.2 meters in the EX group and 82.2 ± 92.5 meters in the EX+T group.

Our original power computations assumed a 20% dropout rate during the 6-month intervention period. However, results to date from current STEP-HI participants are far better than that. As of the end of February, 2020, only two of the 25 women randomized to the EX groups chose not to use the gel (after randomization) and are participating in the study in all other respects; a third subject cannot participate fully in the exercise program due to an ankle fracture. She also continues to participate in all other aspects of the protocol and will provide follow-up data, as able. Impressively, STEP-HI has not had any study participants withdraw to date among the 38 randomized participants, 13 of whom have completed the 6-month intervention period, with 22 having been in the study for four months or more. Based on this experience we perform revised power computations using assumed dropout rates of 5% and 10%.

Because the primary goal of the study involves comparisons between the EX and the EX+T groups, ***we have constructed a revised protocol that changes the randomization ratio such that starting with this version of the protocol, more subjects would be randomized into the EX groups than into the Enhanced Usual Care (EUC) group***. Specifically, we have constructed a revised randomization scheme whose purpose would be to complete randomization with 70 subjects in the EX group, 70 in the EX+T group, and 28 in the EUC group (total N = 168). If the total N turns out to be 120, the randomization scheme will yield 50 subjects in each EX group and 20 in the EUC group.

Using the projected 6MWD changes (42.7 $\pm$ 69.2 meters in the EX group and 82.2 $\pm$ 92.5 meters in the EX+T group), assuming two-sided tests at the 0.05 level of significance, and assuming that the number of non-dropouts will be either 66 / 47 per group (5% dropout rate with the two sample sizes reflecting a total N of 168 or 120) or 63 / 45 (10% dropout rate), we get the following results: For a total N of 168, the power is 0.79 for a 5% dropout rate and 0.77 for a 10% dropout rate. If N=120, the power is 0.64 for a 5% dropout rate and 0.62 if the dropout rate is 10%. These results are summarized in Table 1.

<u>Table 1</u>: Power computations comparing the baseline to six month change in 6MWD in the EX group with the corresponding change in the EX+T group. Results are based on two sided tests at the 0.05 level of significance.

Total sample size	Number randomized to each intervention group	Dropout rate	
		5%	10%
168	70	0.79	0.77
120	50	0.64	0.62

While these power values are less than ideal, we emphasize that they are only marginally below the initially planned 0.8 when the sample size is 168. The reasons for this relatively high power despite a reduction in sample size from the originally proposed n=300 is the imbalanced randomization we are proposing and the very positive results we have experienced thus far with respect to dropout rates.

We note also that if the number of randomized subjects were to turn out to be only 120, the power is reduced to 0.64 and 0.62, depending on the presumed dropout rate. While this is clearly lower than we would like, we emphasize that power values in this range leave a reasonable chance of achieving significance if our effect size projections are accurate. Moreover, when interim analyses are performed in group sequential trials that permit stopping early due to futility, it is standard to stop early when the conditional power is less than 0.2 or 0.3. We are aware of no study where a conditional power as high as 0.62 or 0.64 led to early stopping for reasons of futility.

Section 20: Statistical Analysis

The analysis of data will be based on the Intention-to-Treat principle under which all patients randomized in each group will be analyzed as a part of that group regardless of subsequent events.

20.1 Specific Aim 1

Addresses the question of whether T therapy combined with supervised exercise (EX+T) will induce greater improvements in the 6MWD relative to the EUC and EX conditions alone. The primary goal of this study are to determine if there are between-group differences in the change over time in 6MWD. Analytic strategies are based on the fact that all outcomes, including secondary outcomes, are continuous variables scheduled to be measured on at least three occasions. Initial analyses will include chi square tests and t-tests to compare baseline values of key demographic, clinical, and physiologic parameters. Because we expect the primary outcomes to be normally distributed or transformable to normality, between group comparisons of 6MWD will be performed using mixed model repeated measures analysis of variance. All mixed model analyses will be implemented using PROC MIXED in SAS and will be preceded by an evaluation of the data, intended to determine what covariance structure should be used. Since past experience suggests that correlation coefficients between time points will be greater for time points that are closer together than for time points that are further apart, we anticipate that an autoregressive or a Toeplitz covariance structure will be appropriate for these analyses. If correlation coefficients are approximately constant regardless of the time between assessment points, we expect that a compound symmetric covariance structure will fit the data best. Decisions about which covariance structure will be implemented will be made only after correlation coefficients across time have been evaluated and with appropriate considerations of the Schwarz-Bayesian criterion and Akaike's Information criterion. All other things being equal, we will give preference to correlation structures that require the estimation of the smallest number of parameters. The primary analysis will be a mixed model repeated measures analysis of variance that evaluates statistical contrasts to determine if the change from baseline to 6 months for EX+T are significantly different compared to EX alone and compared to EUC. In addition to the primary analysis, statistical contrasts will be evaluated to determine if there are significant between-group differences in the change from baseline to 3 months. Additional analyses of covariance will be performed that adjust for baseline values and covariates that differed across groups at baseline.

20.2 Specific Aim 2

The mixed model approach discussed above will be applied also to secondary endpoints. In all of the analyses we perform, we will assess the appropriateness of the selected analytic methods by determining whether required distributional assumptions are satisfied. Thus, when t-tests are performed, we will evaluate normality and equal variance assumptions before accepting the results of the test. When analyses of variance and covariance are performed, regression residuals will be evaluated to ensure that normality assumptions are satisfied. When necessary assumptions are violated, potentially useful data transformations will be considered and evaluated. In some circumstances, inappropriate distributional properties in combination with the failure to find an appropriate data transformation may lead to a decision to use non parametric rather than parametric methods of analysis. Thus, when we say above that we will perform a t-test, it is possible that the t-test will be performed on the log transformed data or that we will use Wilcoxon's test as an alternative. It is similarly possible that we will apply our mixed

model analyses to transformed data or that it will be necessary to perform these analyses semi-parametrically using the ranks of the data rather than the data themselves.

Missing data will be a particular concern in our mixed model analyses. To address this issue, we will collect information about reasons for the missingness to facilitate an assessment of whether missing data are ignorable. To formally determine whether data are missing completely at random, we will use a SAS macro to implement the likelihood ratio based method of Little [(117)]. When data are missing completely at random, multiple imputation using PROC MI in SAS will be used to impute missing values and standard mixed model analyses will incorporate these imputed values. Understanding the reasons for missingness and assessments of whether variable values at baseline (when data are complete) are associated with the likelihood that they are subsequently missing will help us decide whether the data are missing at random. Unfortunately, it is not possible to test whether missing data are non-ignorably missing because the very definition of such data implies that the missing data depend on other data that are not available. If we believe that data may be non-ignorably missing, we will adopt a conservative approach to analysis and impute values that bias towards the null hypothesis. For example, if evidence suggests that subjects may have dropped out for reasons related to limited response to the interventions, we will bias towards the null by assuming that the missing data on these subjects are equal to the worst value of the outcome measure that was observed for any subject at the time points that are missing. To minimize the burden of missing data, we will encourage all subjects who withdraw from the study to undergo the final assessments that, at the very least, provide information about the primary endpoints. Our experience suggests that many subjects who choose to drop out are willing to cooperate in this way. Finally, when we suspect non-ignorable missingness, we will perform sensitivity analyses that use more than one approach to imputation in order to evaluate the robustness of our conclusions.

20.3 Specific Aim 3

Specific Aim 3 addresses the question of whether the effects of the interventions persist after three months of discontinuing them. Analyses in this aim will be similar to those described for the primary specific aim in that they will employ mixed model repeated measures analysis of variance. The central differences are that analyses for this aim will include the 9 month time point and will focus on the statistical contrast that performs a between-group comparison of changes from 6 to 9 months for EX+T relative to EX. In addition to p-values, we will generate confidence bounds that quantify the changes from 6 to 9 months within each group and the potential differences between the 6 to 9 month changes in different groups. We omit the details of these analyses and emphasize only that considerations related to determining covariance structures and dealing with missing data are identical to those previously discussed.

Section 21: Clinical Coordinating Center (CCC) Activities

21.1 Study Protocol, Manual of Procedures, and Informed Consent

21.1.1 Study Protocol

The CCC will be responsible for finalizing the study protocol, including securing approval of the DSMB. The CCC will maintain detailed records of all protocol amendments and distribute amendments to clinical sites in a timely manner. They will also ensure each clinical site's compliance with protocol amendment changes.

21.1.2 Manual of Procedures

The CCC will finalize and maintain the study Manual of Procedures (**MOP**). This includes ensuring that the MOP is in agreement with the study protocol and any amendments.

21.1.3 Informed Consent

The CCC will develop a study Informed Consent Form (ICF) for distribution to the clinical sites. Individual sites will submit contact information, injury and HIPAA language that will be placed into the approved consent template by the CCC. Please see Section 23.2 Informed Consent Forms (ICF) for more information on the ICF.

21.2 Clinical Site Coordination

21.2.1 Budgets

Budgets will be negotiated with each clinical site in accordance with NIA guidelines.

21.2.2 Regulatory

Washington University's IRB (WU IRB) will serve as the single IRB (sIRB) for this study. The CCC will assure all reliance documents are collected and the consent form is approved with standard language. The CCC will coordinate with each site to verify local IRB reliance on the sIRB at WU, completion of the Signature and Delegation Log, and submission of Form FDA 1572. Further, the CCC will require that all study staff have completed the appropriate Human Subjects training and that each site maintain records for those staff members. The CCC can audit these records at any time to assure compliance.

The CCC will also maintain a record of the study at the ClinicalTrials.gov website.

21.3 Clinical Site Visits

The CCC will coordinate all visits to study sites, including:

21.3.1 Site Initiation

The initiation visit will serve as verification by the CCC that each clinical site is in compliance with the study protocol and regulatory authorities prior to enrolling participants. The initiation visit will also serve as training verification for study staff at each site. The initiation visit will be conducted at each site prior to the enrollment of the first subject. The initiation visit may be conducted in conjunction with the DCC to ensure that each site is also adequately prepared to collect and transmit accurate study data.

21.3.2 Site Monitoring

Site monitoring visits will be conducted by the CCC and DCC, to ensure that each site is in compliance with study procedures, regulatory approvals and documentation, and data collection, maintenance, and entry. Monitoring visits will be conducted after the participant randomization begins at each site (as scheduling permits) and annually thereafter, unless routine quality control measures determine that a site is having difficulty with some aspect of the protocol, which would warrant more frequent visits. The DCC may be able to perform some visits remotely. Please see Section 22.3.6: Site Visits for more information on site monitoring visits.

21.4 Communication with Clinical Sites

The CCC will provide centralized communication with all clinical sites. With the exception of information specific to the DCC, the CCC will be responsible for coordination of all information sent to sites, including updates to the protocol or Manual of Procedures, scheduling of meetings and/or conference calls, newsletters with updates on study enrollment, and other information as needed.

21.5 Reportable Adverse Event (RAE) Reporting

The CCC will collect all information on RAEs from each clinical site and provide the appropriate follow-up. The CCC will be responsible for communicating between the sites and the ISM, as well as communicating back to the site with changes or recommendations regarding participant safety. The CCC will report RAEs to the DSMB, FDA, NIA, and sIRB per the reporting requirements of each entity. The CCC will also provide reports back to the clinical sites as recommended for participant safety by the ISM, FDA, or sIRB.

21.6 Meetings and Training Sessions

21.6.1 Investigator Meeting

Prior to study commencement, the CCC will conduct an Investigator Meeting at a location central to all sites. The purpose of the meeting is to allow site PIs and select study staff to convene and discuss details of the protocol and conduct of the study, as well as complete relevant training. The CCC will be responsible for all aspects of coordinating the meeting, including but not limited to: travel and accommodations, meeting agenda, and meeting documentation.

21.6.2 Steering Committee and DSMB Calls

The CCC will coordinate calls for the study Steering Committee, DSMB, and any scientific oversight subcommittee of the Steering Committee or CCC. The CCC will be responsible for scheduling calls, providing an agenda, and keeping minutes and making these available to each site. Steering Committee meetings will be held at least monthly, but may be more often during the first year of the project or when issues arise requiring more discussion by the Steering Committee.

21.6.3 Training Sessions

Training sessions for study procedures, as required per study protocol or for the purposes of the DCC, will be coordinated by the CCC. This includes scheduling with appropriate staff members, including any necessary travel arrangements, and creation of training materials. The CCC will also facilitate the transfer of training certifications between each clinical site and the DCC.

21.6.4 Clinical Site Conference Calls

After initiation of the first clinical site, the CCC will conduct regular conference calls at least monthly, with study site coordinators. The calls will allow study coordinators to receive updates about study progress and discuss any issues with recruitment, screening, enrollment, and general conduct of the study. The CCC will also coordinate monthly phone calls with the Supervising Physical Therapists and exercise interventionists. The CCC will be responsible for scheduling calls, providing an agenda, and keeping minutes and making these available to each site.

21.7 Study Equipment and Website

Standardized study equipment and supplies for physical performance testing will be distributed by the CCC. The CCC will procure all equipment/materials and coordinate with each site to ensure timely shipping and receipt.

The CCC will maintain a study website for purposes related to recruitment.

21.8 Collaboration with the Data Coordinating Center

The CCC may conduct study activities with the DCC where necessary to ensure efficient conduct of the study and accurate collection of data. Instances where the CCC and DCC collaborate, where not outlined above, will be at the discretion of the study Steering Committee and the DCC.

21.9 Central Lab, Pharmacy, and DXA Coordination

The CCC will contract with a central lab at the Washington University School of Medicine for laboratory testing procedures. The CCC will coordinate with the lab to detail the types of lab tests needed along with the time frame and reporting responsibilities. The CCC will assure that only the unblinded CCC physician receives specific laboratory test results to preserve blinding.

The CCC will coordinate with the University of Iowa Pharmaceuticals to facilitate the distribution of study drug and placebo to the clinical sites. This will include finalizing contracts with the company and providing timelines for study milestones. The CCC will also create a standardized system for supplying the University of Iowa Pharmaceuticals with information about study enrollment at each site. The CCC will be responsible for coordinating drug/placebo shipment to the sites.

The CCC will provide centralized oversight and analysis of DXA scans at UCD. Detailed procedures are described in the MOP.

Section 22: Data Coordinating Center (DCC) Activities

In addition to its focus discussed in Sample Size and Statistical Power and Statistical Analysis, the DCC will be responsible for data management, quality control, randomization, and scientific oversight as the study progresses. Specifically, DCC activities will include:

1. Designing, testing, maintaining, and providing instructions on the use of a password protected web-based data entry and management system; performing quality control assessments of the data entered into that system, and creating and maintaining the SAS analysis datasets that will serve the data analytic needs of the study;
2. Preparing regular reports which monitor adverse events and the progress of the study for the Steering Committee and the Data and Safety Monitoring Board;
3. Performing ongoing quality control assessments such as annual site visits (in coordination with the CCC) and the sending of queries when inappropriate missing or potentially erroneous data are identified;
4. Developing and maintaining WUSTL Box for report & document storage;
5. Developing and maintaining a web based randomization scheme.

22.1 DCC Responsibilities during the Study Start-Up Period

The study start-up period is the developmental phase of the study that precedes subject recruitment. During that period, the DCC will develop the data management and quality control systems, activities that are discussed in sections Data Management and Quality Control. In this section, we discuss two other DCC activities that will take place during this period.

22.1.1 Forms Design

During the study start-up period, the DCC will work with clinic investigators to design study forms. General principles that will guide forms design and selection include ensuring that the data collected are relevant to study objectives, a physical appearance of forms that facilitates the collection of accurate and complete data, the avoidance of open ended “fill in the blank” questions because they may not be suitable for statistical analysis, the use of validated instruments wherever possible, a focus on simple and unambiguous language, the use of categories like “don’t know” to deal as appropriate with situations where the subject cannot knowledgeably answer “yes” or “no” to a question, and constant attention to the burden of data collection on both subjects and staff.

22.1.2 Training and Certifying Personnel

The DCC will work closely with the CCC and the clinical sites to ensure the appropriate training and certification of personnel who perform study tasks. These efforts will focus on ensuring that (1) investigators, clinical coordinators, and their designated backups have appropriate familiarity with the details of the protocol; (2) all evaluations are carried out by individuals who are certified as being knowledgeable about and experienced with relevant study procedures; and (3) personnel are familiar and comfortable with data entry and management procedures. To accomplish these goals, the following procedures will be carried out:

1. The DCC will work with the CCC to help facilitate the Investigator Meeting. The DCC will be responsible for reviewing the standardization of data collection with attendees and providing hands on experience with data collection procedures.

2. Certification of data entry personnel will require that they establish familiarity with the system by entering sham data from appropriate forms on a set of test subjects.
3. At least two individuals will be certified to perform every task to ensure that backup is always available.
4. The identification number of the individual performing a particular assessment will be included on data forms and entered into the computer. Using a DCC maintained list of certified personnel, it will be routinely confirmed by the DCC that assessments have been performed by certified individuals.
5. Modified data on forms will be initialed and dated by the person making the change.
6. To maximize efficiency, personnel who join the study after it begins will be certified by the clinic at which they work.

22.2 Data Management

A central focus of the DCC will be on the development and testing of a secure web based data entry and management system. In the sections that follow, we discuss the REDCap (Research Electronic Data Capture) system that will serve our needs in this domain.

22.2.1 The REDCap System

REDCap is a comprehensive data entry, management, and quality control system developed at Vanderbilt University. It bridges the gap between data entry systems such as SAS that can be difficult to design and use and systems like Excel that are easy to use but that lack critical quality control capabilities. REDCap is widely used at many Universities throughout the United States and Europe and has been the data entry system of choice for more than 4500 projects at Washington University. The Division of Biostatistics has used REDCap in numerous studies, including four previous multicenter trials we have coordinated.

REDCap provides secure, web-based applications with an intuitive interface for users to enter data with real time validations rules such as range checks for categorical and continuous variables, automatic branching logic, checks for internal consistency within a form, and calculation of derived variables. REDCap includes data access groups to limit user access to data records associated with their clinical site only, audit trails for tracking data manipulation, and data export to common statistical packages.

22.2.2 Testing the Data Management System at the DCC

After the REDCap data entry screens have been created, the first step in testing the system will be internal to the DCC where complete sets of forms containing sham data will be completed and entered into REDCap. The data will then be transferred to SAS datasets and item by item comparisons between forms and SAS printouts will be performed. The preliminary test forms will intentionally contain outlying and missing data to confirm that range checks and requirements for completing certain fields are functioning properly. A second level of testing which will precede the enrollment of subjects will occur when data entry personnel are asked, as part of their training and certification, to enter sham data from simulated study visits. As with the DCC data tests, item by item comparisons between forms and SAS printouts will be performed at the DCC. In addition to confirming the convenience and completeness of the data entry screens, these procedures will evaluate all components of the data management process ranging from edit checking, to skip logic in the REDCap system, to the automated transfer of REDCap data to SAS, to the correctness of the programs that create and store the SAS

datasets. As we discuss in section Quality Control of Forms and the Data Entry System, these procedures will be closely integrated with searches for ambiguous wording, the training of data entry personnel, and the modification of an original set of draft forms into a final version.

22.2.3 Data Security and Confidentiality

Important features of REDCap and standard procedures of the Division of Biostatistics at Washington University provide a high degree of certainty that data will never be lost and that subject confidentiality will be maintained. In accordance with the two key requirements of HIPAA, password protection will be required both for access into study computers and REDCap. Only authorized personnel will be given access to the data entry system and those personnel will only be able to access data from their own clinic site. All web-based information transmission from and to REDCap is encrypted. Standard security and confidentiality measures at the DCC include the requirement that employees sign confidentiality agreements, personal identifiers are included in electronic databases only under strong necessity, and encryption is used when names, addresses, and other primary identifiers are present in SAS datasets. The security of data at the DCC will be ensured by a combination of password protection and rigorous automated policies regarding the backup of data. Access to all systems in our Division is restricted to the Division's own faculty, staff, and collaborators. Access to accounts which store data from this study will be restricted to DCC personnel and the Division's network manager. All file transfers to outside computers use secure transfer methods which ensure that all such traffic is encrypted over the network. Access to all computers and to REDCap is automatically logged. Finally, should problems arise with the system, a full-time, highly qualified, network engineer runs the system and is on call (with backup) 24 hours a day, 7 days a week.

22.2.4 The SAS Data Codebook

All data collected by REDCap and data received as supplemental files will be transferred into SAS using automated procedures for use in future data analyses and reports. Generally, each data collection form/instrument is stored in its own SAS dataset. Each dataset in the database will contain appropriate labels and formats. To facilitate use of this database, the DCC will prepare and maintain a detailed "SAS Data Codebook", which describes the content, structure, and layout of the SAS database. It is intended to be a complete and self-explanatory description of each variable retained in the SAS database. The DCC routinely uses such manuals in multicenter studies and in large program projects for a variety of reasons, the most important being that they greatly facilitate data management and analysis by making it very easy to find where, in a large database, an item on a particular form is stored.

22.2.5 Data/Resource Sharing Plan

In the final year of the award all data will be cleaned and archived for long term storage. After the completion of the final data edits, DCC staff will initiate the process of permitting qualified researchers to gain access to the STEP-HI dataset. Our data/resource sharing plan includes the following components.

1. The DCC will prepare a de-identified database that is consistent with HIPAA requirements. In order to protect the rights and privacy of subjects in the study, no identifiers that could permit linkages to individual research participants, and variables that could lead to deductive disclosure of the identity of individual subjects will not be

included nor will indirect identifiers such as infrequently occurring (e.g., fewer than 20 patients) outwardly manifest characteristics such as limb amputation be included in the data file. Categories that contain small numbers of patients (e.g., unusual fracture types) will be combined. The data for sharing will be provided to the NIA/NIH after the end of the period of funding for use with other investigators according to NIH policy.

2. The de-identified database will include all STEP-HI research data and will be stored in a repository at Washington University at a time that will coincide with the online publication of the STEP-HI primary endpoint paper.
3. The repository will contain documentation designed to facilitate use of the database. That documentation will include a data dictionary that describes the content and structure of each dataset, that contains variable names and labels, and that maps specific variable names to items on questionnaires so users can see the exact wording of questions and precise formats that are attached to each variable.
4. The documentation prepared for the repository will include a list and timetable for secondary and exploratory papers planned by STEP-HI investigators. The purpose of this list is to avoid circumstances where researchers request access to the database in order to perform analyses that are already planned or underway by STEP-HI investigators.
5. STEP-HI investigators will assume responsibility for adjudicating requests for data Researchers seeking access to the data will be required to complete a brief questionnaire. The questionnaire will request information that describes the background and rationale for the work to be undertaken along with pre-specified research questions and/or hypotheses, the goals of the planned analyses, the analytic methods to address those goals, and identifies the specific variables in the database that are required for those analyses. The investigator making the request and the data analyst (if not the same person) will be asked to provide a current CV or NIH-type biosketch.
6. Access to the STEP-HI database will be restricted to those investigators who certify that they will not use the data for commercial purposes or purposes that go beyond the pre-specified research questions and/or hypotheses.

22.3 Quality Control

The DCC has a broad view of quality control in clinical research as a multifaceted process that addresses concerns such as the accuracy and completeness of computerized data, the common administration of protocols across sites, monitoring adherence to protocol requirements, and the training and certification of personnel. Other quality control measures we will implement include:

22.3.1 Ensuring Data Accuracy and Completeness

REDCap has a number of built-in quality control features that are focused on ensuring the accuracy and completeness of the data and that are accomplished at the clinics when the data are entered. Other quality control measures involve actions taken at the DCC, features of the data forms that help facilitate high quality data, and steps taken at the clinic as data forms are completed. Because the system automatically keeps a log of who entered or changed all data, we will be able to discuss with the data enterer any concerns about a particular data item. Other quality control measures which will help ensure the accuracy and completeness of data include:

1. Range checks will flag values that are outside a predefined expected range but that may be plausible;
2. Prevent the entry of values that are considered to be impossible;
3. Accept only a predefined set of values for categorical measures;
4. All data forms will contain the identification number of the person who completed the form, facilitating easy access to the source if there are legibility or other problems with a form.
5. Forms and screens will have a “not done” option for key assessments so the associated missing data will be understood as appropriately missing.
6. Investigators and study coordinators will be expected to do visual checks of all completed forms to confirm legibility, completeness, and reasonableness as each form is filled out.

22.3.2 Data Queries

To ensure data quality, data checks will be implemented through programmed data edit checks implemented by the DCC and reported to the clinical sites as structured data queries. Queries issued by the DCC (a) will include complete data integrity checks that are impractical and/or impossible to generate in REDCap, (b) may overlap with data flags that were overridden in REDCap, but that require additional attention, and (c) will include data issues that require human interpretation and/or judgement. Potentially problematic data will be reported to each clinical site through WUSTL Box for investigation, correction, and resolution. After investigation, the clinical site will perform the necessary corrections and/or verifications and provide resolution documentation within 14 days via WUSTL Box. If data corrections are necessary, edits will be made by the site within REDCap.

22.3.3 Data Audits

The DCC will conduct an annual item by item audit of data. To that end, the DCC will randomly select a subset of the subjects at each clinical site. Depending on logistical considerations, the audit will be performed either during site visits or remotely at the DCC using requested copies of the forms stored at the clinical site. During the audit, every item on the data forms will be compared with the computerized data and records will be kept of error rates at each clinical site. It will be confirmed that changes on forms have been appropriately initialed and documented, and all identified problems will be discussed with the relevant clinic personnel and, if necessary, with the Steering Committee. Following each audit, a detailed report will be prepared and distributed to all clinic sites. Audits will be timed to precede meetings of the DSMB so that the DCC can respond to questions about data quality.

22.3.4 Quality Control of Forms and the Data Entry System

In addition to the testing outlined in Section Testing the Data Management System at the DCC, the following series of steps will precede enrollment of the first subject.

1. Staff at each clinic will be asked to enter data from one set of draft forms in a formal search for ambiguous wording.
2. Data collection personnel will test relevant forms by entering sham data for a simulated study visit. These forms will be used to confirm the familiarity of data entry personnel with the system, as a test of the system itself, and as part of the certification process.

3. After these procedures yield modifications of the draft forms, the DCC will finish developing the REDCap screens and will perform a final set of internal tests focused on ensuring that any needed modifications have been correctly incorporated into the revised system.

22.3.5 Summary Reports

The DCC will prepare summary reports of study progress in response to requests by the Steering Committee and the DSMB. It will also prepare regular reports using automated features to be built into the SAS database. Reports will consider such issues as (1) recruitment rates, (2) the proportion of recruited subjects who are study eligible, (3) the degree to which there are data forms that should be in REDCap, but are not, (4) the missing data rate (5) the time interval between completing a form and entering it in REDCap, (6) rates of compliance to study procedures, and (7) adverse event rates. They will also discuss adherence to the study protocol based on the number of supervised exercise training sessions attended and the pump bottle weights recorded. These reports will be updated routinely and made available online through WUSTL Box. Because they will be site-specific as well as aggregate in their presentation, they will be an important component of our quality control efforts as they will help identify areas of inadequate performance at the clinical sites. The adverse event report will differ from other reports in that severe and potentially study related adverse events will be reported to the sIRB, IRBs and the chair of the DSMB within the required time frame.

22.3.6 Site Visits

Site visits will be coordinated with the CCC. Site visitors will prepare an agenda that will include observations of the performance of study procedures and of the filing system that is used to store completed data forms. Site visitors will confirm that data forms are correctly filed, that required training certificates are available, will do random data audits if these audits are not done remotely, will check the specific forms of subjects whose data may have been problematic in the past, and will confirm that changes on forms are appropriately initialed and dated by the person who made the changes. Site visits will generally be conducted by a staff member from the DCC and a staff member from the CCC.

22.4 WUSTL Box

WUSTL Box is a secure, HIPAA compliant, cloud file storage and collaboration tool offered by Washington University. Password protected file stores on WUSTL Box will be designed for internal use by study personnel only. General access stores will serve as a repository for study documents such as the Manual of Procedures, study forms, a bibliography of study publications, and the regular reports prepared by the DCC. Limited access file stores will be restricted by clinical site and contain site-specific reports and query documentation.

22.5 Randomization

The DCC will create an online, password protected, randomization system that will be used to facilitate the random assignment of subjects to the three study arms. The system will be created using the built-in randomization module of the REDCap system that will be used for data entry. To avoid temporal bias, randomization will be blocked within clinical site using random block sizes in order to preclude the possibility that investigators might know in advance the assignment of the last subject in a particular block.

Section 23: Participant Rights and Confidentiality

23.1 Institutional Review Board (IRB)

Washington University's IRB (WU IRB) will serve as the single IRB (sIRB) for this study. Institutions will sign a reliance agreement to document reliance on the WU sIRB. WU sIRB will be responsible for IRB approval of all protocols, informed consent documents, recruitment documents and any other associated study documents. Investigators will be required to report all reportable events to the CCC, who will in turn alert the WU sIRB within the required reporting timeframes. Modifications to the protocol or informed consent documents are required to be approved by the WU sIRB before initiating the change.

Sites must follow requirements by their local IRB policies to assure the local IRB has the appropriate documentation and are alerted to the pertinent modifications. The study chair will be responsible for sending DSMB recommendations to individual clinical site PIs, who may in turn be required to distribute the report to their local IRBs.

23.2 Informed Consent Forms (ICF)

It will be the sole responsibility of each clinical site PI to ensure that informed consent was properly obtained for every participant who entered into the study at her/his site. The ICF will describe the purpose of the study, the procedures to be followed, alternatives to participation, and the risks and benefits of participation. It will also be explained that signing the consent form allows the study to confirm eligibility before randomization to a treatment group. Written informed consent will be obtained according to procedures reviewed and approved by the WU sIRB. Informed consent will be obtained prior to the screening visit procedures. Consent by a legally authorized representative will not be accepted.

The clinical sites will be responsible for ensuring that the correct version of the ICF is used at their site.

If there is a change in any of the study procedures or risks that may affect the participant, the ICF must be revised and undergo appropriate sIRB review and approval. Any participants enrolled in the study prior to such changes and who are still active in the study must sign the amended consent form.

The study consent form will be provided to a potential participant for review prior to obtaining informed consent. The ICF may also be mailed to the participant and/or a family member so that she has sufficient time to read the document and, if desired, to have a family member or friend review the form before signing. During the informed consent process, study staff will provide participants with adequate information concerning the study procedures, respond to individuals' questions and concerns, and ensure that each individual understands all the information provided by assessing ability to provide informed consent. A more detailed description of the informed consent process can be found in Section 8.3.

23.2.1 Disposition of Informed Consent Forms

Because ICFs contain subject identifiers and protected health information (PHI), these forms will not be submitted with the data collection forms. Originals of the ICF will be filed and maintained by the clinical site coordinator in the participant binder which will be secured in a locked cabinet or office. A copy of the signed consent form will be given to the participant and this fact will be documented in the participant's record.

23.2.2 HIPAA Authorization

The HIPAA Authorization for Research is an individual's signed permission to allow the study investigators to use or disclose the individual's PHI described in the authorization. Once an individual has agreed to participate in the study and written informed consent has been obtained, the HIPAA Authorization for Research must also be explained and signed. The HIPAA Authorization may be a stand-alone document or wording for the HIPAA Authorization may be incorporated into the text of the ICF. The original signed authorization will be submitted to the study office and a signed copy will be given to the participant.

23.3 Participant Confidentiality

Potential participants will be provided with a clear understanding of how the information they provide will be used. All investigators and staff involved in the study will be required to complete training on the protection of human subjects and HIPAA and to maintain proper certifications.

To ensure confidentiality of study data, completed questionnaires and study forms will be kept in participant binders stored in locked offices at each clinical site, no unauthorized person will be permitted to see the binder or forms, names will be used only for the necessary purpose of making sure that the recorded information is for the person to whom it refers, and data will be summarized so that published results cannot be traced to individuals.

On data collection forms, participants will be identified only by a unique study identification number. Clinical sites will record names, contact information, and other direct identifiers to enable them to maintain contact with participants. Logs accessible only to the local clinical site PIs and key study personnel will link the study identification number to names.

To protect study data from theft or unauthorized perusal or alteration, access to all computer files will be restricted to designated personnel through the use of passwords. Access to the database and programs will be on a "need to use" basis (e.g., coordination staff cannot access main system programs).

Computer security procedures, including multiple levels of password protection will be instituted. The study records will be identified by a unique participant identification number. Identification numbers will be recorded on each page of the paper forms. The participant's study status date (e.g., date of randomization) will be used as a second level of check. Final analysis data sets will not contain any directly or indirectly identifying information. Thus, dates of birth will be converted to age, other dates will be changed to counts of days from study entry, identification numbers will be replaced with sequence numbers, variables that could lead to deductive disclosure of the identity of individual participants will not be included nor will indirect identifiers such as infrequently occurring (e.g., fewer than 20 participants), outwardly manifest characteristics.

In the final year of award, after the completion of a final data edit, DCC staff will prepare a data file in SAS containing all study variables intended for use in publications; identifying information and administrative data (e.g., audit trail) will not be included in this data file. A de-identified data set will be created which merges all the essential data from all time points of the study. Documentation (including abstracts of published works and calculated variable definitions) and form images will be included with the data file. The data file will be provided to the NIA after the end of the period of funding for sharing with other investigators according to NIH policy.

Section 24: Research Publication Policy

Publications will be operationally defined as manuscripts for publication; abstracts for platform or poster presentation at scientific and other professional meetings; slides for presentation at scientific and other professional meetings; doctoral dissertations; and master's theses.

The goal of our publication policy is to encourage and facilitate the publication of study results. The purposes of this policy are to ensure the following: 1) STEP-HI publications will be of the highest scientific quality; 2) STEP-HI will be described in a consistent manner across publications; 3) measures are reported in consistent ways across publications; 4) proper acknowledgements are included; and 5) appropriate authorship credit is determined prior to submission of manuscripts for publication consideration.

Publications from STEP-HI will be overseen by the Publications Committee (PC). The PC will be led by the director of the DCC and include at least one representative from the CCC and representatives from at least two participating clinical sites. The PC will make recommendations to the SC.

A document describing the STEP-HI Publication Policy and Procedures appears in the Manual of Procedures.

Section 25: Ancillary Studies Policies

An ancillary study will be defined as a study that 1) uses supplementary data that will be collected on participants who are recruited in STEP-HI, over and above the data collection required by the STEP-HI protocol; 2) collects biological specimens (e.g., blood) or performs diagnostic tests (e.g., bone density scans); and/or 3) collects data on subjects not enrolled in STEP-HI but who may be compared to STEP-HI subjects (e.g., participants who receive an alternate intervention). Ancillary studies will be distinct from databank studies, which use data previously collected on participants who are enrolled in STEP-HI.

Ancillary studies will be reviewed and approved by the Ancillary Studies Committee (**ASC**) and ratified by the SC prior to initiation to ensure that they do not conflict with the main study protocol. All approved ancillary studies will also be reviewed by the DSMB and NIA prior to initiation. If approved, the ancillary study PI will report to the DSMB on the same schedule as the main study. Review by the ASC (and approval by the SC) will also be required for presentation or publication of ancillary study results.

STEP-HI investigators will be encouraged to consider ancillary studies and to involve other investigators, within and outside of STEP-HI personnel. Participation in an ancillary study will be subject to the approval of the STEP-HI ASC, SC, and DSMB. The following factors will be considered in determining approval of a proposed ancillary study:

1. Participant burden
    a. The proposed study must be acceptable to the participants (e.g. in terms of time, discomfort, privacy).
    b. The proposed study must not reduce enrollment or hamper continued participation in the main study.
2. Study interference
    a. The proposed study must not interfere with other parts of the main study.
    b. The proposed study must put no additional demand on STEP-HI resources.
3. The proposed study must be of the highest scientific merit.
4. The investigators must have adequate resources to effectively complete the ancillary study, including:
    a. Sufficient budget (including enough to offset any costs to STEP-HI).
    b. Staff having the requisite expertise to meet the objectives of the project.

25.1 Concurrent Studies

Study investigators agree not to conduct studies which would directly compete with or have a detrimental effect on the conduct of STEP-HI during the period of recruitment and follow-up. However, it is understood that each clinical site has the right to conduct concurrent studies with participants who do not meet criteria for enrollment into STEP-HI. Concurrent studies of patients who meet eligibility criteria for STEP-HI but are not enrolled in STEP-HI must be disclosed and reviewed by the STEP-HI SC.

Section 26: Skeletal Muscle Mass using Creatine Dilution Method

This section describes an optional ancillary study to the main STEP-HI study. Participation is voluntary. This ancillary study follows the STEP-HI protocol regarding the overall study organization, administration, adverse event reporting, participant rights, publication policy, and general activities of the CCC and DCC.

26.1 General Information

Ancillary Study Name: Assessment of Skeletal Muscle Mass Using D3-Creatine Dilution in Frail Female Hip Fracture Patients.

Ancillary Study PI: Ellen Binder, MD at Washington University School of Medicine

Ancillary Study Funding Source: NIH Administrative Supplement

Participating Sites: All STEP-HI Sites are able to participate

Compounding Pharmacy:

Greenpark Compounding Pharmacy
4061-F Bellaire Blvd
Houston, TX 77025

Processing Laboratory:

University of California, Berkeley
Hellerstein Lab/Shubha Shankaran
Dept of Nutritional Sci & Toxicology
54 Mulford Hall
Berkeley, CA 94720
Phone: (510) 642-0646

26.2 Background and Significance

A challenge of research focused on age-associated loss of skeletal mass (sarcopenia), is that the relationship between muscle mass and physical performance and mobility has been inconsistent across a number of studies of older adults. The reported inconsistencies may be due to limitations of current measurement techniques. Computed tomography and magnetic resonance imaging usually measure select muscle groups, which do not provide an assessment of total body skeletal muscle. Dual x-ray absorptiometry does not directly measure muscle mass, but calculates it by subtracting the non-bone, non-fat components of total body mass[118]. Factors such as variations in tissue hydration can also confound accurate measurement of skeletal muscle and lean mass. Thus, the finding that skeletal muscle mass is only weakly associated with functional outcomes in older adults may be due to current muscle-specific or indirect techniques for measurement of skeletal muscle mass.

The deuterated-creatine (D3-creatine, D3Cr) dilution method is a novel measurement of total body muscle mass. A single oral dose of D3Cr is absorbed and then diluted by entry into the endogenous pool of creatine in skeletal muscle. The dilution method uses similar assumptions as 24-hour creatinine assessment of muscle mass, i.e., that approximately 98% of total body creatine is sequestered in skeletal muscle, which is subsequently metabolized to creatinine and

excreted in the urine. Preliminary studies indicate that D3Cr dilution provides a direct and accurate measurement of creatine pool size and skeletal muscle mass[119] and, in a recent study of older men, was associated with functional capacity measures and risk of falls and disability[120]. More studies are necessary to validate the use of D3Cr as an accurate measure of skeletal muscle mass. The STEP-HI study provides a unique opportunity to address this issue.

Experience to date is that the D3Cr dilution protocol has been straightforward to implement in a clinical setting. A recent report examined the accuracy and potential variability of the D3Cr dilution method[121]. The estimate of variability in that report is higher than what would result in the current application as those investigators measured the same individual 3-4 months after the initial assessment, a period of time when body composition may change (subjects were elderly and some with chronic heart failure) but did not account for time in their analyses, likely adding considerable variability to their estimate.

26.3 Study Objectives

26.3.1 Specific Aim 1

To determine whether post-intervention (month 6) D3-creatine (D3Cr) muscle mass is significantly greater for EX+T relative to EX alone, and relative to EUC, after adjusting for baseline D3Cr.

26.3.2 Specific Aim 2

To obtain preliminary estimates of the associations between baseline-adjusted post-intervention D3Cr muscle mass with baseline-adjusted post-intervention: a) skeletal muscle strength, as measured by Leg-Press 1-repetition maximum (1-RM) and Chair Rise time, b) muscular endurance, as measured by Six-Minute Walk Distance (6MWD) and, c) global function, measured by the Modified Physical Performance Test (mPPT) score and the Short Physical Performance Battery (SPB) score.

26.3.3 Specific Aim 3

To estimate the responsiveness of D3Cr muscle mass to change, we will compare post-intervention D3Cr muscle mass with post-intervention DXA total body lean mass, after adjusting for the associated baseline values.

26.3.4 Specific Aim 4

To compare the associations between D3Cr muscle mass and functional measures at baseline, with the associations between DXA total body mass and functional measures at baseline.

26.4 Participants

100 women will be recruited to this ancillary study.

26.4.1 Inclusion Criteria

1. Women eligible for and participating in the STEP-HI study.
2. Women who have signed the consent for this ancillary study.

26.2.2 Exclusion Criteria

1. Women who are ineligible for the STEP-HI Study.
2. Women with a urinary drainage bag.

26.5 Study Design

Participants will be asked to ingest a small tablet containing 30 mg of D3-creatine, which will be administered at the research center by study staff or sent home with instructions to self-administer in their home. Depending on the day of the week that the dose is ingested, participants will be asked to provide a fasting morning void urine sample (prior to consumption of food) 2-6 days after ingesting the dose. Because of the challenges of collecting a fasting urine sample in this frail population, we will offer to obtain the urine sample from the participant at their home or during an assessment visit for the STEP-HI Study. Urine will be collected in a collection cup labeled with the participant's ID number. One mL of the sample will be aliquoted into a 2.0 mL vial which will be frozen in a -20 freezer. The samples will then be batch shipped to the central lab at the University of California-Berkeley for detection of labeled and unlabeled creatinine by LC/MS. Samples will be shipped from each clinical site to the central lab once or twice per year.

The dose of labeled creatine will be compounded by Greenpark Compounding Pharmacy (Houston, TX), and shipped to each participating site.

Ingestion of the D3-creatine pill will occur at 2 time points:

- Baseline visit (or shortly after randomization) for the STEP-HI Study
- Month 6 visit (or shortly after) for the STEP-HI Study

Providing a fasting urine sample will occur at 3 time points:

- Baseline visit (2-6 days after taking the Baseline D3-Creatine pill)
- Prior to the Month 6 research visit (0-5 days before taking the Month 6 D3-creatine pill)
- Month 6 visit (2-6 days after taking the Month 6 D3-Creatine pill)

The first D3-Creatine measure will ideally be collected during the baseline visits, but can be obtained up to 1 week after randomization. The second measure will be collected ideally during the 6 month STEP-HI assessment, but can be obtained up to 1 week after the completion of the Month 6 procedures.

26.6 Recruitment and Retention

26.6.1 Informed Consent

A separate written informed consent document and appropriate HIPAA authorizations will be obtained in compliance with procedures reviewed and approved by the sIRB. A copy of the informed consent form will always be provided to potential participants to allow for adequate review of the information. Prior to obtaining informed consent, time will be given to the participant to review the consent form and ask questions. Study staff will summarize all components of the ICF and remind participants that participation in this ancillary study is voluntary. The consent form for this ancillary study will not be given to participants until after they are deemed eligible for the STEP-HI study. The consent review may occur any time after determining screening eligibility until randomization. For women who provide informed consent, the original will be maintained at the clinical site and a copy will be given to the participant. Informed consent will be completed prior to beginning any research procedures for this ancillary study.

26.6.2 Enrollment

Date of enrollment for this ancillary study is considered the date of consent.

26.6.3 Study Withdrawal

This ancillary study will follow the same protocol for study withdrawal as the STEP-HI study. In the event that a participant voluntarily withdraws from the assessments of the STEP-HI study, she will be unable to continue in this ancillary study. If a participant is willing to complete the 6 month assessment visit or an early withdrawal visit, this ancillary study would be offered as part of those optional assessments.

26.6.4 Study Discontinuation

This ancillary study will follow the same protocol for discontinuation as the STEP-HI study. In the event that a participant is discontinued from the STEP-HI study, she will also be discontinued from this ancillary study.

26.6.5 Replacement of Subjects

Participants who withdraw or are discontinued from this ancillary study will not be replaced.

26.7 Study Measures – University of California-Berkeley

Assessment of urinary D3-creatinine enrichment is completed by LC-MS/MS. The assessments will be performed at the University of California-Berkeley under the supervision of Dr. William Evans. 100 µl aliquots of urine are processed independently for Cr/Crn concentration and D3-Crn enrichment. For Cr/Crn concentration determination, 100 µl of urine or standards for the standard curve is added to 100 µl internal standard and 200 µl acetonitrile. Samples are centrifuged to precipitate proteins and aliquots of the supernatant are diluted 50 fold using 70% acetonitrile for LC/MS analysis. Samples are injected onto a Cogent Diamond Hydride column (4.0x75mm) using an Agilent 1260 UPLC and are separated on a gradient consisting of acetonitrile with 0.1% formic Acid (Solvent A) and water with 0.1% formic acid (Solvent B). Mass spectrometry is performed on a Sciex 6500 QTRAP operating in the multiple reaction monitoring (MRM) mode. For determining the enrichment of D3-Crn, quantitation is performed using a standard curve for D3-Crn and D3-Cr enrichment that spanned from 0% to 0.168% and is measured by MRM transitions (116.1/46.1) corresponding to the M2 peak of Crn and 117.1/47.1 which corresponds to D3-Crn. Samples are run in duplicate and average values are reported. Samples with CVs greater than 10% will be subjected to reanalysis. The total body Cr pool size is calculated as retained (delivered) D3-Cr dose divided by D3-Crn enrichment and muscle mass as described previously.

26.8 Safety

26.8.1 Risks

There are no known risks of taking creatine. Labeled creatine is a stable isotope; there is no radioactivity. Creatine is a normal component of animal protein; the average person consumes 1 gram (1,000 mg) of creatine per day. The dose for the study is 30 mg. There may be other unknown risks or risks that we did not anticipate associated with creatine. To track these risks the study will record and monitor all adverse events as in the main study.

26.8.2 Adverse Event Reporting

This ancillary study will rely to the main study to collect all new or worsening symptoms as reported by participants during STEP-HI baseline and follow-up visits. Participation in this ancillary study will be documented for all RAEs.

26.8.3 Site Monitoring

Enrollment eligibility, consent disposition, and adherence to procedures will be monitored by the STEP-HI DCC using data entered into REDCap and by the CCC during STEP-HI study monitoring visits.

26.9 Statistical Analysis

26.9.1 Approach for Specific Aim 1

Aim 1 addresses the question of whether T therapy combined with supervised exercise (EX+T) will induce greater improvements in D3Cr relative to the EUC and EX conditions alone. Initial analyses will include chi -square tests and t-tests to compare baseline values of key demographic, clinical, and D3Cr measurements. We will compare post-treatment D3Cr muscle mass using analysis of covariance (ANCOVA) where month 6 D3Cr muscle mass is the dependent variable, treatment group is the independent variable, and baseline D3Cr muscle mass is the covariate. Statistical contrasts within the ANCOVA will test whether participants assigned to EX+T have more D3Cr muscle mass at month 6 compared to EX alone and compared to EUC. Analyses will be implemented using SAS and will be preceded by an evaluation of the data. If statistical assumptions are not satisfied, transformations will be explored or nonparametric covariance analysis will be used.

26.9.2 Approach for Specific Aim 2

Aim 2 will estimate the association of month 6 D3Cr muscle mass with month 6 strength, endurance, and functional measures. We will use generalized linear models where month 6 D3Cr muscle mass is the dependent variable and baseline D3Cr muscle mass and treatment group are covariates. Separate models will be developed in which the independent variables are measures of strength, endurance, and function at baseline or for the change between baseline and month 6. Standardized beta coefficients with 95% confidence bounds will be reported.

26.9.3 Approach for Specific Aim 3

Aim 3 will compare D3Cr muscle mass at month 6 with DXA total body lean mass at month 6, after adjusting for baseline. We will use generalized linear models that are analogous to those described in SA 2.

26.9.4 Approach for Specific Aim 4

Aim 4 will compare the strength of associations between D3Cr muscle mass and functional measures at baseline with associations between DXA total body mass and functional measures at baseline. Linear regression modeling and overall model r-squares will be used to quantify associations between variables. There will be one model for each dependent variable (i.e., D3Cr muscle mass and DXA total body mass) and each independent variable (i.e., functional measure). Additional multivariable regression models will be explored to determine if the strength of the associations of D3Cr muscle mass with function, and the strength of the associations of DXA total body mass with function are modified by other potential confounding

variables such as age, race, comorbidities, and nutritional intake. We will perform diagnostics of residuals and collinearity and take corrective action if assumptions are violated.

Section 27: References

1. Gallagher JC, Melton LJ, Riggs BL, Berstrath E. Epidemiology of fractures of the proximal femur in Rochester, Minnesota. Clin Orthop. 1980;150:163-71.
2. Lewinnek GE, Kelsey J, White Iii AA, Kreiger NJ. The significance and a comparative analysis of the epidemiology of hip fractures. Clin Orthop. 1980;152:35-43.
3. Cummings SR, Black DM, Rubin SM. Lifetime risks of hip, Colles', or veterbral fracture and coronary heart disease among white postmenopausal women. Arch Intern Med. 1989;149:2445-8.
4. Cummings SR, Nevitt MC, Browner WS, Stone K, Fox KM, Ensrud KE, et al. Risk factors for hip fracture in white women. N Engl J Med. 1995;332:767-73.
5. Cummings SR, Phillips SL, Wheat ME. Recovery of function after hip fracture: the role of social supports. J Am Geriatr Soc. 1988;36:801-6.
6. Jette AM, Harris BA, Cleary PD, Campion EW. Functional recovery after hip fracture. Arch Phys Med Rehabil. 1987;68:735-40.
7. Koval KJ, Skovron ML, Polatsch D, Aharonoff GB, Zuckerman JD. Dependency after hip fracture in geriatrics patients: a study of predictive factors. J Orthop Trauma. 1996;10:531-5.
8. Magaziner J, Simonsick EM, Kashner TM, Hebel JR, Kenzora JE. Predictors of functional recovery one year following hospital discharge for hip fracture: A prospective study. J Gerontol Med Sci. 1990;45:M101-M7.
9. Marottoli RA, Berkman LF, Cooney LM. Decline in physical function following hip fracture. J Am Geriatr Soc. 1992;40:861-6.
10. Magaziner J, Hawkes W, Hebel JR, Zimmerman SI, Fox KM, Dolan M, et al. Recovery from hip fracture in eight areas of function. J Gerontol Med Sci. 2000;55:498-507.
11. Handoll HH, Cameron ID, Mak JC, Finnegan TP. Multidisciplinary rehabilitation for older people with hip fractures. Cochrane Database Syst Rev. 2009(4):CD007125.
12. Handoll HH, Sherrington C, Mak JC. Interventions for improving mobility after hip fracture surgery in adults. Cochrane Database Syst Rev. 2011(3):CD001704.
13. Binder E, Brown MB, Sinacore DR, Steger-May K, Yarasheski KE, Schechtman KB. Effects of Extended Outpatient Rehabilitation After Hip Fracture, A Randomized Controlled Trial. Journal of the American Medical Association. 2004;292:837-46.
14. Latham NK, Harris BA, Bean JF, Heeren T, Goodyear C, Zawacki S, et al. Effect of a home-based exercise program on functional recovery following rehabilitation after hip fracture: a randomized clinical trial. Journal of the American Medical Association. 2014;311(7):700-8.
15. Cooper C, Barker DJ, Wickham C. Physical activity, muscle strength, and calcium intake in fracture of the proximal femur in Britain. Br Med J. 1988;297:1443-7.
16. Farmer ME, Harris T, Madans JH, Wallace RB, Cornoni-Huntley J, White LR. Anthropometric indicators and hip fracture. J Am Geriatr Soc. 1989;37:9-16.
17. Fox KM, Magaziner J, Hawkes WG, Yu-Yahiro J, Hebel JR, Zimmerman SI, et al. Loss of bone density and lean body mass after hip fracture. Osteoporos Int. 2000;11:31-5.
18. Visser M, Kritchevsky SB, Newman AB, Nevitt M, Stamm E, Harris TB. Leg muscle mass and composition in relation to lower extremity performance in men and women aged 70 to 79: the health, aging and body composition study. J Am Geriatr Soc. 2002;50:897-904.
19. Jozsi AC, Campbell WW, Joseph L, Davey SL, Evans WJ. Changes in power with resistance training in older and younger men and women. J Gerontol Med Sci. 1999;54A:M591-M6.
20. Foldvari M, Clark M, Laviolette LC, Bernstein MA, Kaliton D, et al. Association of muscle power with functional status in community-dwelling elderly women. J Gerontol Biol Med Sci. 2001;55:M192-M9.
21. Melton LJ, 3rd, Khosla S, Crowson CS, O'Connor MK, O'Fallon WM, Riggs BL. Epidemiology of sarcopenia. J Am Geriatr Soc. 2000;48:625-30.

22. Janssen I, Heymsfeld SB, Ross R. Low relative skeletal muscle mass (sarcopenia) in older persons is associated with functional impairment and physical disability. J Am Geriatr Soc. 2002;50:889-96.
23. Bean JF, Kiely DK, Herman S, Leveille SG, Mizer K. The relationship between leg power and physical performance in mobility-limited older people. J Am Geriatr Soc. 2002;50:461-7.
24. Sternfeld B, Ngo L, Satariano WA, Tager IB. Associations of body composition with physical performance and self-reported functional limitations in elderly men and women. Am J Epidemiol. 2002;156:110-21.
25. Basaria S, Dobs A. Hypogonadism and androgen replacement therapy in elderly men. Am J Med. 2001;110(7):563-72.
26. Morley JE, Kaiser FE, Perry HM, Morley PP, Stauber PM, Vellas B, et al. Longitudinal changes in testosterone, lutenizing hormone, and follicle-stimulating hormone in healthy older men. Metabolism. 1997;46:410-3.
27. Tenover JL. Experience with testosterone replacement in the elderly. Mayo Clin Proc. 2000;75:S77-S82.
28. Bhasin S, Buckwalter JG. Testosterone supplementation in older men: A rationale idea whose time has not yet come. J Andrology. 2001;22:718-31.
29. Katznelson L, Finkelstein JS, Schoenfeld DA, Rosenthal DI, Anderson EJ, Klibanski A. Increase in bone density and lean body mass during testosterone administration in men with acquired hypogonadism. J Clin Endocrinol Metab. 1996;81:4358-65.
30. Wang C, Swerdloff RS, Iranmanesh A, Dobs A, Snyder PJ, Cunningham G, et al. Effects of transdermal testosterone gel on bone turnover markers and bone mineral density in hypogonadal men. Clin Endocrinol (OXF). 2001;54:739-50.
31. Bhasin S, Woodhouse L, Storer TW. Proof of the effect of testosterone on skeletal muscle. J Endocrinol. 2001;170:27-38.
32. Doherty TJ. Invited review: Aging and sarcopenia. J.Appl.Physiol. 2003;95(4):1717-27.
33. Padero MM, Bhasin S, Friedman TC. Androgen supplementation in older women: too much hype, not enough data. J Am Geriatr Soc. 2002;50:1131-40.
34. Lobo RA. Androgens in postmenopausal women: production, possible role, and replacement options. Obstet Gynecol Surv. 2001;56(6):361-76.
35. Judd HL, Judd GE, Lucas WE, Yen SSC. Endocrine function of the postmenopausal ovary: concentration of androgens and estrogens in ovarian and peripheral vein blood. J Clin Endocrinol Metab. 1974;39:1020-4.
36. Burger HG, Dudley EC, Cui J, Dennerstein L, Hopper JL. A prospective longitudinal study of serum testosterone, dehydroepiandrosterone sulfate, and sex hormone-binding globulin levels through the menopause transition. J Clin Endocrinol Metab. 2000;85:2832-8.
37. Khosla S, Melton LJ, Atkinson EJ, O'Fallon WM, Klee GG, Riggs BL. Relationship of serum sex steroid levels and bone turnover markers with bone mineral density in men and women: a key role for bioavailable estrogen. J Clin Endocrinol Metab. 1998;83:2266-74.
38. Roger M, Nahoul K, Schjoller R. Evolution with ageing of four plasma androgens in postmenopausal women. Maturitas. 1980;2:171-7.
39. Longcope C. Adrenal and gonadal androgen secretion in normal females. Clin Endocrinol Metab. 1986;15:213-28.
40. Davison SL, Bell R, Donath S, Montalto JG, Davis SR. Androgen levels in adult females: changes with age, menopause, and oophorectomy. J.Clin.Endocrinol.Metab. 2005;90(7):3847-53.
41. Rariy CM, Ratcliffe SJ, weinstein R, Bhasin S, Blackman MR, Cauley JA, et al. Higher serum free testosterone concentration in older women is associated wih greater bone mineral density, lean body mass, and total fat mass: The Cardiovascular Health Study. J Clin Endocrinol Metab. 2011;96:989-96.

42. Khosla S, Melton LJ, Achenbach SJ, Oberg AL, Riggs BL. Hormonal and biochemical determinants of trabecular microstructure at the ultradistal radius in women and men. J Clin Endocrinol Metab. 2005;91(3):885-91.
43. Roddam AW, Appleby P, Neale R, Dowset M, Folkerd E, Tipper S, et al. Association between endogenous plasma hormone concentrations and fracture risk in men and women: the EPIC-Oxford prospective cohort study. J Bone Miner Metab. 2009;27:485-93.
44. Lee JS, LaCroix AZ, Wu L, Cauley JA, Jackson RD, Kooperberg C, et al. Associations of serum sex hormone-binding globulin and sex hormone concentraions with hip fracture risk in postmenopausal women. J Clin Endocrinol Metab. 2008;93:1796-803.
45. Davidson BJ, Ross RK, Paganini-Hill A, Hammond GD, Siiteri PK, Judd HL. Total and free estrogens and androgens in postmenopausal women with hip fractures. J Clin Endocrinol Metab. 1982;54:115-20.
46. Jassal SK, Barrett-Connor E, Edelstein SL. Low bioavailable testosterone levels predict future height loss in postmenopausal women. J Bone Min Res. 1995;10(4):650-4.
47. Dubin NH, Monahan LK, Yu-Yahiro JA, Michael RH, Zimmerman SI, Hawkes W, et al. Serum concentrations of steroids, parathyroid hormone, and calcitonin in postmenopausal women during the year following hip fracture: effect of location of fracture and age. J Gerontol Med Sci. 1999;54:M467-M73.
48. Douchi T, Yamamoto S, Yoshimitsu N, Andoh T, Nagata Y. Relative contribution of aging and menopause to changes in lean and fat mass in segmental regions. Maturitas. 2002;42:301-6.
49. Aloia JF, Vaswani A, Russo L, Sheehan M, Flaster E. The Influence of menopause and hormonal replacement therapy on body cell mass and body fat mass. Am J Obstet Gynecol. 1995;172:896-900.
50. Hakkinen K, Pakarinen A. Muscle strength and serum testosterone, cortisol and SHBG concentrations in middle-aged and elderly men and women. Acta Physiol Scand. 1993;148:199-207.
51. Gower BA, Nyman L. Associations among oral estrogen use, free testosterone concentration, and lean body mass among postmenopausal women. J Clin Endocrinol Metab. 2000;85:4476-80.
52. Iannuzzi-Sucich M, Prestwood KM, Kenny AM. Prevalence of sarcopenia and predictors of skeletal muscle mass in healthy, older men and women. J Gerontol Biol Med Sci. 2002;57A:M772-M7.
53. Morley JE, Perry HM. Androgens and women at the menopause and beyond. J Gerontol Med Biol Sci. 2003;58A:M409-M16.
54. Bhasin S, Storer TW, Berman N, Callegari C, Clevenger B, Phillips J, et al. The effects of supraphysiologic doses of testosterone on muscle size and strength in normal men. N Engl J Med. 1996;335(1):1-7.
55. Wang C, Swerdloff RS, Iranmanesh A, Dobs A, Snyder PJ, Cunningham G, et al. Transdermal testosterone gel improves sexual function, mood, muscle strength, and body composition parameters in hypogonadal men. J Clin Endocrinol Metab. 2000;85:2839-53.
56. Brodsky IG, Balagopal P, Nair KS. Effects of testosterone replacement on muscle mass and muscle protein synthesis in hypogonadal men - A clinical resarch center study. J Clin Endocrinol Metab. 1996;81:3469-78.
57. Snyder PJ, Peachey H, Berlin JA, Hannoush P, Haddad G, Dlewati A, et al. Effects of testosterone replacement in hypogonadal men. J Clin Endocrinol Metab. 2000;85:2670-7.
58. Snyder PJ, Peachey H, Hannoush P, Berlin JA, Loh L, Lenrow DA, et al. Effect of testosterone treatment on body composition and muscle strength in men over 65 years of age. J Clin Endocrinol Metab. 1999;84:2647-53.
59. Isidori AM, Giannetta E, Greco EA, Gianfrilli D, Bonifacio V, Isidori A, et al. Effects of testosterone on body composition, bone metabolism and serum lipid profile in middle-aged men: a meta-analysis. Clin.Endocrinol.(Oxf). 2005;63(3):280-93.

60. Sattler FR, Castaneda-Sceppa C, Binder EF, Schroeder ET, Wang Y, Bhasin S, et al. Testosterone and growth hormone improve body composition and muscle performance in older men. J Clin Endocrinol Metab. 2009;94(6):1991-2001.
61. Tenover JL. Testosterone replacement therapy in older adult men. Int J Androl. 1999;22:300-6.
62. Tenover JSA. Androgen replacement therapy to reverse and/or prevent age-associated sarcopenia in men. Bailliers's Clin Endrocrinol Metab. 1998;12:419-25.
63. Bakhshi V, Elliott M, Gentili A, Godschalk M, Mulligan T. Testosterone improves rehabilitation outcomes in ill older men. J Am Geriatr Soc. 2000;48:550-3.
64. Amory JK, Chansky HA, Chansky KL, Camuso MR, Hoey CT, et al. Preoperative supraphysiological testosterone in older men undergoing knee replacement. J Am Geriatr Soc. 2002;50:1698-701.
65. Sheffield-Moore M, Urban RJ, Wolf SE, Jiang J, Catlin DH, Herndon DN, et al. Short-term oxandrolone administration stimulates net muscle protein synthesis in young men. J Clin Endocrinol Metab. 1999;84:2705-11.
66. Wolfe R, Ferrando A, Sheffield-Moore M, Urban R. Testosterone and muscle protein metabolism. Mayo Clin Proc. 2000;75:S55-S60.
67. Strawford A, Barbieri T, Van Loan M, Parks E, Catlin D, Barton N, et al. Resistance exercise and supraphysiologic androgen therapy in eugonadal men with HIV-related weight loss: A randomized controlled trial. Journal of the American Medical Association. 1999;281(14):1282-90.
68. Demling RH, DeSanti L. Oxandrolone, an anabolic steroid, significantly increases the rate of weight gain in the recovery phase after major burns. J Trauma. 1997;43:47-51.
69. Demling RH, DeSanti L. The rate of restoration of body weight after burn injury, using the anabolic agent oxandrolone, is not age dependent. Burnes. 2001;17:S46-S51.
70. Yeh S, DeGuzman B, Kramer T. Reversal of chronic obstructive pulmonary disease associated weight loss using the anabolic agent oxandrolone. Chest. 2002;122:421-8.
71. Srinivas-Shankar U, Roberts SA, Connolly MJ, O'Connell MDL, Adams JE, Oldham JA, et al. Effects of testosterone on muscle strength, physical function, body composition, and quality of life in intermediate-frail and frail elderly men: A randomized, double-bline, placebo-controlled study. J Clin Endocrinol Metab. 2010;95:639-50.
72. Basaria S, Coviello AD, Travison TG, Storer TW, Farwell WR, Jette AM, et al. Adverse events associated with testosterone administration. N Engl J Med. 2010;363(2):109-22.
73. Lovejoy JC, Bray GA, Bourgereois MO, Macchiavelli R, Rood JC, Greeson C, et al. Exogenous androgens influence body composition and regional body fat distribution in obese postmenopausal women: a clinical research center study. J Clin Endocrinol Metab. 1996;81:2198-203.
74. Davis SR, Walker KZ. Effects of estradiol with and without testosterone on body composition and relationships with lipids in postmenopausal women. Menopause. 2000;7:395-401.
75. Dobs A, Nguyen T, Pace T, Roberts CR. Differential effects of oral estrogen versus oral estrogen-androgen replacement therapy on body composition in postmenopausal women. J Clin Endocrinol Metab. 2002;87:1509-16.
76. Huang G, Basaria S, Travison TG, Ho MH, Davda M, Mazer NA, et al. Testosterone dose-response relationships in hysterectomized women with or without oophorectormy: effects on sexual function, body composition, muscle performance and physical function in a randomized trial. Menopause. 2014;21(6):1-11.
77. Miller K, Corcoran C, Armstrong C, Caramelli KE, Anderson E, et al. Transdermal testosterone administration in women with acquired immunodeficiency syndrome wasting: a pilot study. J Clin Endocrinol Metab. 1998:2717-25.
78. Dolan S, Wilkie S, Aliabadi N, Sullivan MP, Basgoz N, Davis B, et al. Effects of testosterone administration in human immunodeficiency virus-infected women with low weight: a randomized placebo-controlled study. Arch.Intern.Med. 2004;164(8):897-904.

79. Choi HH, Gray PB, Storer TW, Calof OM, Woodhouse L, Singh AB, et al. Effects of testosterone replacement in human immunodeficiency virus-infected women with weight loss. J.Clin.Endocrinol.Metab. 2005;90(3):1531-41.
80. Wierman ME, Arlt W, Basson R, Davis SR, Miller KK, Murad MH, et al. Androgen therapy in women: a reappraisal: an Endocrine Society clinical practice guideline. J Clin Endocrinol Metab. 2014;99(10):3489-510.
81. Elraiyah T, Sonbol MB, Wang Z, Khairalseed T, Asi N, Undavalli C, et al. Clinical review: The benefits and harms of systemic testosterone therapy in postmenopausal women with normal adrenal function: a systematic review and meta-analysis. J Clin Endocrinol Metab. 2014;99(10):3543-50.
82. Ganesan K, Habboush Y, Sultan S. Transdermal Testosterone in Female Hypoactive Sexual Desire Disorder: A Rapid Qualitative Systematic Review Using Grading of Recommendations Assessment, Development and Evaluation. Cureus. 2018;10(3).
83. Achilli C, Pundir J, Ramanathan P, Sabatini L, Hamoda H, Panay N. Efficacy and safety of transdermal testosterone in postmenopausal women with hypoactive sexual desire disorder: a systematic review and meta-analysis. Fertility and sterility. 2017;107(2):475-82. e15.
84. Lord SR, Menz HB. Physiologic, psychologic, and health predictors of 6-minute walk performance in older people. Archives of physical medicine and rehabilitation. 2002;83(7):907-11.
85. Enright PL, McBurnie MA, Bittner V, Tracy RP, McNamara R, Arnold A, et al. The 6-min walk test*: A quick measure of functional status in elderly adults. Chest. 2003;123(2):387-98.
86. Brown M, Sinacore DR, Binder EF, Kohrt WM. Physical and performance measures for the identification of mild to moderate frailty. J Gerontol A Biol Sci Med Sci. 2000;55(6):M350-5.
87. Guralnik JM, Simonsick EM, Ferrucci L, Glynn RJ, Berkman LF, Blazer DG, et al. A short physical performance battery assessing lower extremity function: association with self-reported disability and prediction of mortality and nursing home admission. Journal of gerontology. 1994;49(2):M85-M94.
88. Guralnik JM, Ferrucci L, Simonsick EM, Salive ME, Wallace RB. Lower-extremity function in persons over the age of 70 years as a predictor of subsequent disability. New England Journal of Medicine. 1995;332(9):556-62.
89. Jette AM, Cleary PD. Functional disability assessment. Physical Therapy. 1987;67(12):1854-9.
90. Fillenbaum GG. Multidimensional functional assessment of older adults: The Duke Older Americans Resources and Services procedures: Psychology Press; 2013.
91. Johanson N, Charlson M, Szatrowski T, Ranawat C. A self-administered hip-rating questionnaire for the assessment of outcome after total hip replacement. The Journal of bone and joint surgery. American volume. 1992;74(4):587-97.
92. Hays RD, Bjorner JB, Revicki DA, Spritzer KL, Cella D. Development of physical and mental health summary scores from the patient-reported outcomes measurement information system (PROMIS) global items. Quality of Life Research. 2009;18(7):873-80.
93. Smith BW, Dalen J, Wiggins K, Tooley E, Christopher P, Bernard J. The brief resilience scale: assessing the ability to bounce back. International journal of behavioral medicine. 2008;15(3):194-200.
94. Katzman R, Brown T, Fuld P, Peck A, Schechter R, Schimmel H. Validation of a short Orientation-Memory-Concentration test of cognitive impairment. Am J Psychiatry. 1983;140:734-49.
95. Blessed G, Tomlinson B, Roth M. The association between quantitative measures of dementia and of senile change in the cerebral grey matter of elderly subjects. Br J Psychiatry. 1968:797-811.
96. Binder EF, Williams DB, Schechtman KB, Jeffe DB, Kohrt WM. Effects of hormone replacement on serum lipids in elderly women: results of a randomized, placebo-controlled trial. Ann Intern Med. 2001;134:754-60.
97. Brown M, Holloszy JO. Effects of a low intensity exercise program on selected physical performance characteristics of 60- to 71-year olds. Aging (Milano). 1991;3(2):129-39.

98. Fiatarone Singh MA. Exercise, nutrition and managing hip fracture in older persons. Curr Opin Clin Nutr Metab Care. 2014;17(1):12-24.
99. Paddon-Jones D, Short KR, Campbell WW, Volpi E, Wolfe RR. Role of dietary protein in the sarcopenia of aging. Am J Clin Nutr. 2008;87(5):1562S-6S.
100. Mifflin MD, St Jeor ST, Hill LA, Scott BJ, Daugherty SA, Koh YO. A new predictive equation for resting energy expenditure in healthy individuals. The American journal of clinical nutrition. 1990;51(2):241-7.
101. Gloth FM, Tobin JD. Vitamin D deficiency in older people. J Am Geriatr Soc. 1995;43(7):822-8.
102. Boland R. Role of vitamin D in skeletal muscle function. Endocr Rev. 1986;7(4):434-48.
103. Gallagher JC, Sai A, Templin T, Smith L. Dose response to vitamin D supplementation in postmenopausal women: a randomized trial. Ann Intern Med. 2012;156(6):425-37.
104. Pahor M, Guralnik JM, Ambrosius WT, Blair S, Bonds DE, Church TS, et al. Effect of structured physical activity on prevention of major mobility disability in older adults: the LIFE study randomized clinical trial. JAMA. 2014;311(23):2387-96.
105. McDaniel MA, Binder EF, Bugg JM, Waldum ER, Dufault C, Meyer A, et al. Effects of cognitive training with and without aerobic exercise on cognitively demanding everyday activities. Psychol Aging. 2014;29(3):717-30.
106. Bellg AJ, Borrelli B, Resnick B, Hecht J, Minicucci DS, Ory M, et al. Enhancing treatment fidelity in health behavior change studies: best practices and recommendations from the NIH Behavior Change Consortium. Health Psychol. 2004;23(5):443-51.
107. Resnick B, Inguito P, Orwig D, Yahiro JY, Hawkes W, Werner M, et al. Treatment fidelity in behavior change research: a case example. Nurs Res. 2005;54(2):139-43.
108. Swerdloff RS, Wang C, Cunningham G, Dobs A, Iranmanesh A, et al. Long-term pharmacokinetics of transdermal testosterone gel in hypogonadal men. J Clin Endocrinol Metab. 2000;85:4500-10.
109. Swerdloff RS, Wang C, Iranmanesh A, Dobs A, Snyder PJ, et al. Transdermal testosterone (T) gel is efficacious and safe in older compared to young men. Abstract P-648 presented at Endocrine Society's 84th Annual Meeting. 2002.
110. Wang C, Berman N, Longstreth JA, Chuapoco B, Hull L, et al. Pharmacokinetics of transdermal testosterone gel in hypogonadal men: application of gel at one site versus four sites: a General Clinical Research Center Study. J Clin Endocrinol Metab. 2000;85:964-9.
111. Haskell WL. The efficacy and safety of exercise programs in cardiac rehabilitation. Med Sci Sports Exerc. 1994;26(7):815-23.
112. Gordon NF, Kohl HW, Pollock ML, Vaandrager H, Gibbons LW, Blair SN. Cardiovascular safety of maximal strength testing in healthy adults. Am J Cardiol. 1995;76(11):851-3.
113. Wegner N, Froelicher E, Smith L, al e. Cardiac Rehabilitation as Secondary Prevention. Clinical Practice Guideline No. 17. Rockville, MD: US Dept of Health and Human Services, Public Health Service, Agency for Health Care Policy and Research and the National Heart, Lung, and Blood Institute; 1995.
114. Williams MA, Haskell WL, Ades PA, Amsterdam EA, Bittner V, Franklin BA, et al. Resistance exercise in individuals with and without cardiovascular disease: 2007 update: a scientific statement from the American Heart Association Council on Clinical Cardiology and Council on Nutrition, Physical Activity, and Metabolism. Circulation. 2007;116(5):572-84.
115. Gill TM, DiPietro L, Krumholz HM. Role of exercise stress testing and safety monitoring for older persons starting an exercise program. JAMA. 2000;284(3):342-9.
116. Prior JC, Barr SI, Chow R, Faulkner RA. Prevention and management of osteoporosis: consensus statements from the Scientific Advisory Board of the Osteoporosis Society of Canada. 5. Physical activity as therapy for osteoporosis. CMAJ. 1996;155(7):940-4.
117. Little RJA. A test of missing completely at random for multivariate data with missing values. Journal of the American Statistical Association. 1988;83(404):1198-202.
118. Proctor, D.N., et al., Comparison of techniques to estimate total body skeletal muscle mass in people of different age groups. Am J Physiol, 1999. 277(3): p. E489-95.

119. Evans, W.J., et al., D3 -Creatine dilution and the importance of accuracy in the assessment of skeletal muscle mass. J Cachexia Sarcopenia Muscle, 2019. 10(1): p. 14-21.
120. Cawthon, P.M., et al., Strong Relation Between Muscle Mass Determined by D3-creatine Dilution, Physical Performance, and Incidence of Falls and Mobility Limitations in a Prospective Cohort of Older Men. J Gerontol A Biol Sci Med Sci, 2019. 74(6): p. 844-852.
121. Shankaran, M., et al., Dilution of oral D3 -Creatine to measure creatine pool size and estimate skeletal muscle mass: development of a correction algorithm. J Cachexia Sarcopenia Muscle, 2018. 9(3): p. 540-546.

Protocol Version 5.0 Summary of Changes

Protocol dated September 4, 2018, sIRB approved September 6, 2018

The following changes were made from Version 4.0:

1. **Revised** Roles to standardized across MOP and Protocol
2. **Revised** site order by their assigned numbers
3. **Changed** CON (control) to EUC (Enhanced Usual Care) Group
4. **Revised** protocol violations to minor and major deviations
5. **Deleted** "Registered" before dietician
6. **Changed** outside laboratory results to be used for screening results only
7. **Clarified** between discontinuation (when they study removes a participant) and Withdrawal (when a participant asks to be removed)
8. **Changed** 8 foot walk to 4 meter walk for SPPB component
9. **Added** Physical exam to Baseline and 3 month visits
10. **Clarified** time frame for completing the Baseline visits
11. **Clarified** missed exercise session language to allow make-up sessions any time prior to the end of the intervention
12. **Changed** Training and Certification for the Exercise Interventionists
13. **Changed** monitoring fidelity of exercise sessions
14. **Changed** CBC results to blinded
15. **Changed** Independent Safety Monitor to Internal Safety Monitor
16. **Removed** CPR requirement and replaced with familiarity with site policies to provide or active immediate care when faced with medical emergencies
17. **Removed** detailed module names for health education sessions
18. **Clarified** that the first intervention visit will also be used to orient participants to the equipment, it is not a separate visit
19. **Clarified** there are 48 sessions for exercise, any less will be considered missed.
20. **Clarified** timeline in Study Design overview
21. **Removed** requirement for EUC group to have mammogram following intervention since they are not using gel (similar to the transvaginal ultrasound)

Protocol Version 6.0 Summary of Changes

Protocol dated October 8, 2018, sIRB approved October 31, 2018

The following changes have been made from Version 5.0:

1. **Removed** reference about anthropometric phantom in DXA section (9.3.3)
2. **Revised** heal to heel (14.1)
3. **Changed** screening to allow abbreviated mPPT in home
4. **Deleted** specific Phase 1 and Phase 2 exercises from the exercise protocol
5. **Added** references to the safety background section to justify changes to the consent risk language
6. **Changed** risks to match new references and consent form

Protocol Version 7.0 Summary of Changes

Protocol dated January 15, 2019, sIRB approved February 4, 2019

The following changes were made from Version 6.0:

1. **Changed** Exclusion Criteria #7 to: History of: a) Breast, ovarian, endometrial or cervical cancer with diagnosis within the previous 5 years; b) Breast, ovarian, endometrial or cervical cancer of Stage 2 or higher
2. **Changed** Exclusion Criteria #20 to: History of idiopathic deep venous thrombosis or pulmonary embolus (i.e., not related to period or immobilization or surgery), recurrent or multiple venous thrombi; history of a hypercoagulable state such as Factor V Leiden thrombophilia

Protocol Version 7.1 Summary of Changes

Protocol dated March 22, 2019, sIRB approved March 28, 2019

The following changes were made from Version 7.0:

1. **Changed** (per FDA communication) Exclusion Criteria #7 to: History of a hormone dependent neoplasia
2. **Added** (per FDA communication) breast examination to the Screening physical examination

Protocol Version 8.0 Summary of Changes

Protocol dated April 15, 2019, sIRB approved April 15, 2019

The following changes have been made from Version 7.1:

1. **Clarified** 1-RM testing is done with both legs simultaneously
2. **Clarified** a screening manual breast exam is only needed for women who have had a mammogram within the past 6 months and will not get one during Baseline assessments
3. **Clarified** the Nutritional Assessment will be administered if a participant loses 5% or more of her body weight between *attended* study visits
4. **Changed** the temperature for drug storage to match the definition from the United States Pharmacopeia as suggested by the University of Iowa manufacturing and distributing the gel
5. **Changed** the initial dose of testosterone to 12.5 mg/day (1 pump depression) with the ability to increase to 2 pumps or decrease to 1 pump every other day based on the serum T level
6. **Deleted** the order of assessments from the Early Withdrawal visit

Protocol Version 8.1 Summary of Changes

Protocol dated April 29, 2019, sIRB approved May 6, 2019

The following changes have been made from Version 8.0:

1. **Changed** recruitment period from 16 weeks to 20 weeks post-surgery
2. **Changed** screening period from 14 weeks to 17 weeks post-surgery
3. **Changed** the time between the completion of screening and randomization to 3 weeks combined, deleting the separate time frame for baseline and randomization
4. **Added** "Femoral Neck" to Intracapsular Fracture description
5. **Added** CCC contact for participants who refuse

Protocol Version 9.0 Summary of Changes

Protocol dated June 20, 2019, sIRB approved July 11, 2019

The following changes have been made from Version 8.1:

1. **Changed** Exclusion Criteria #7 (following FDA approval) to: History of: a) Breast, ovarian, endometrial or cervical cancer with diagnosis within the previous 10 years; b) Breast, ovarian, endometrial or cervical cancer of Stage 2 or higher.
2. **Changed** Exclusion Criteria #8 to: Elevated liver transaminase *or ALK* levels ≥ 2.5 times above normal range.
3. **Changed** Data/Resource Sharing Plan (per NIA suggestion)

Protocol Version 10.0 Summary of Changes

Protocol dated August 1, 2019, sIRB approved August 30, 2019

The following changes have been made from Version 9.0:

1. **Clarified** timing of the assessment visits
2. **Revised** Data/Resource Sharing Plan to replace "qualified user" with items needed to determine if data will be shared (background, rationale, research questions, and analytic methods of proposal and CV of researcher)
3. **Added** new Section (#26) for the Skeletal Muscle Mass using D3Cr Measure of Muscle Mass Ancillary study with new references in Section 27 (#118-121)

Protocol Version 11.0 Summary of Changes

Protocol dated January 17, 2020, sIRB approved February 26, 2020

The following changes have been made from Version 10.0:

1. **Added** University of Utah (UofU) and University of Pittsburg Medical Center (UPMC) as participating sites and changed all references from six clinical sites to multiple clinical sites.
2. **Changed** recruitment period from 17 weeks to 22 weeks post-surgery.
3. **Changed** screening period from 17 weeks to 22 weeks post-surgery.

4. **Changed** randomization period from 20 weeks to 24 weeks post-surgery.
5. **Clarified** that assessments are based on weeks since randomization and are approximate (to allow for staff/participant/facility scheduling) according to the windows defined in the MOP.
6. **Changed** inclusion criteria 2 to: Surgical repair of a non-pathologic fracture of the proximal femur (Including: femoral neck or intracapsular, intertrochanteric, and subtrochanteric fractures), with a surgical repair date that is within 24 weeks at randomization. If a revision of such a fracture is performed due to failure of the repair, that surgery revision date may be used to calculate the time frame for the screening and randomization dates.
7. **Changed** inclusion criteria 4 to: Serum total testosterone level <60 ng/dL.
8. **Clarified** exclusion criteria 8 to replace ALK (lab abbreviation) with alkaline phosphatase
9. **Changed** exclusion 9 to add UofU: Erythrocytosis defined as hematocrit $\geq 51\%$ at all sites but UCD and UofU, or $\geq 52\%$ at the UCD and UofU sites.
10. **Clarified** inclusion criteria 15: Levels outside of the given range require site physician documentation addressing treatment or absence of thyroid disease and approval by the CCC.
11. **Changed** inclusion criteria 20 to: History of idiopathic deep venous thrombosis or pulmonary embolus (i.e., not related to period or immobilization or surgery), any pulmonary embolus less than 12 weeks prior to the first screening visit, recurrent or multiple venous thrombi; history of a hypercoagulable state such as Factor V Leiden thrombophilia.
12. **Changed** all screening laboratories to required.
13. **Deleted** redraw from screening to baseline of CBC, CMP, & Serum Testosterone (Total & Free)**.**
14. **Added** Activas/Teva as a manufacturer of Testosterone 1% gel.
15. **Changed** the frequency of gel bottle return from monthly, to every other month is supply allows.
16. **Changed** the reviewer off all screening labs to the site physician, instead of having the unblinded CCC physician review CBC and Testosterone results.
17. **Changed** Specific Aim 1 to read: The primary analysis will be a mixed model repeated measures analysis of variance that evaluates statistical contrasts to determine if the change from baseline to 6 months for EX+T are significantly different compared to EX alone and compared to EUC. In addition to the primary analysis, statistical contrasts will be evaluated to determine if there are significant between-group differences in the change from baseline to 3 months.
18. **Changed** Specific Aim 3 to read: The central differences are that analyses for this aim will include the 9 month time point and will focus on the statistical contrast that performs a between-group comparison of changes from 6 to 9 months for EX+T relative to EX. In addition to p-values, we will generate confidence bounds that quantify the changes from 6 to 9 months within each group and the potential differences between the 6 to 9 month changes in different groups.

Protocol Version 12.0 Summary of Changes

Protocol dated June 11, 2020, sIRB approved June 24, 2020

The following changes have been made from Version 11.0:

1. **Changed** the randomization goal from 300 participants to 168 participants.

2. **Changed** the number randomized to each group from 100 to 70 in the EX+T group, 70 in the EX+P group, and 28 in the EUC group. These means that newly randomized participants have roughly a 1 in 9 chance of being randomized to the EUC group with the other 8 being assigned to an EX group (4 to T and 4 to P).
3. **Added** a revised randomization strategy using equipoise stratified randomization.
4. **Changed** the sample size and statistical power section to reflect the new changes to group size and distribution allowed by low dropout rates.
5. **Changed** the Central laboratory from Northwest Research Laboratory to Washington University Core Laboratory for Clinical Studies.
6. **Added** option for participants who refuse participation due to COVID-19 exposure concerns the ability to choose a remote training option if they have a reliable home internet connection. Selection of the remote exercise versus onsite exercise does not guarantee randomization into an exercise group, EUC is still a possibility. If randomized to exercise, limited equipment would be provided to conduct HIPAA compliant video assisted exercise sessions in their homes. The screening, baseline, and monthly assessments would still be conducted onsite.
7. **Added** safety considerations for exercise sessions completed remotely.
8. **Added** the opportunity for certain questionnaire data to be collected via remove video call or telephone interview to limit in-person contact.
9. **Added** the ability to perform blood draws in the participant's home.

Protocol Version 12.1 Summary of Changes

Protocol dated July 24, 2020, sIRB approved August 13, 2020

The following changes have been made from Version 12.0:

1. **Deleted** ability to randomize to a remote exercise group. However, documentation was left for participants to perform some exercise sessions at home in the event of center shutdown or COVID-19 restrictions.
2. **Deleted** revised randomization strategy using equipoise stratified randomization.

Protocol Version 12.2 Summary of Changes

Protocol dated September 16, 2020, sIRB approved September 23, 2020

The following changes have been made from Version 12.1:

1. **Changed** the number of D3-Creatine participants from 50 per group to 100 participants regardless of group assignment.
2. **Changed** the days a sample can be collected after ingestion of the D3-creatine capsule from 3-6 days to 2-6 days.
3. **Added** a urine collection point prior to ingesting the 2nd D3-creatine capsule.

Protocol Version 12.3 Summary of Changes

Protocol dated December 1, 2021, sIRB approved December 1, 2021

The following changes have been made from Version 12.2:

1. **Changed** the number of randomized participants to read between 120 and 168 throughout.
2. **Added** "similar pharmacy" to the University of Iowa Pharmaceuticals to be able to supply the placebo.
3. **Changed** the overall recruitment period from 32 to 38 months.
4. **Added** the following table to clarify the number of participants in each study group based on the number of randomized participants:

Randomized Participants	Participants in EX+T	Participants in EX+P	Participants in EUC
300	100	100	100
168	70	70	28
120	50	50	20

5. **Changed** the Research Publication Policy to include a representative from at least 2 participating clinical sites instead of each participating clinical site.

Protocol Version 12.4 Changes

Dated May 31, 2022, sIRB approved June 6, 2022

The following changes have been made from Version 12.3:

1. **Changed** the overall recruitment period from 38 to 45 months.

Protocol Version 12.5 Changes

Dated November 15, 2022, sIRB approved November 16, 2022

The following changes have been made from Version 12.4:

1. **Added** the discontinuation of gel in EX participants who experience adverse events due to an acute cardiovascular event.
