# Supplementary material for: Combining Exercise Training and Testosterone Therapy in Older Women After Hip Fracture: The STEP-HI Randomized Clinical Trial
Source: JAMA Netw Open. 2025 May 15;8(5):e2510512. doi: 10.1001/jamanetworkopen.2025.10512 (PMC12082367; doi:10.1001/jamanetworkopen.2025.10512)
Supplement: Supplement 2. — eFigure 1. Six-Minute Walk Distance at Baseline, Week 12, and Week 24 eFigure 2. Short Physical Performance Battery (SPPB) Score at Baseline, Week 12, and Week 24 eTable 1. Participants With Adverse Events from Falls by Treatment Group eTable 2. List of Serious Adverse Events by System Organ Class and Preferred Term by Treatment Group eTable 3. List of Serious Adverse Events by System Organ Class and Preferred Term by Severity eTable 4. Laboratory Test Results by Study Visit and Treatment Group eTable 5. Ferriman-Gallwey Hirsutism Scores by Study Visit and Treatment Group [file jamanetwopen-e2510512-s002.pdf]

Supplemental Online Content

Binder EF, Bartley JM, Berry SD, et al. Combining exercise training and testosterone therapy in older women after hip fracture: the STEP-HI randomized clinical trial. *JAMA Netw Open*. 2025;8(5):e2510512. doi:10.1001/jamanetworkopen.2025.10512

eFigure 1. Six-Minute Walk Distance at Baseline, Week 12, and Week 24

eFigure 2. Short Physical Performance Battery (SPPB) Score at Baseline, Week 12, and Week 24

eTable 1. Participants With Adverse Events from Falls by Treatment Group

eTable 2. List of Serious Adverse Events by System Organ Class and Preferred Term by Treatment Group

eTable 3. List of Serious Adverse Events by System Organ Class and Preferred Term by Severity

eTable 4. Laboratory Test Results by Study Visit and Treatment Group

eTable 5. Ferriman-Gallwey Hirsutism Scores by Study Visit and Treatment Group

This supplemental material has been provided by the authors to give readers additional information about their work.

© 2025 Binder EF et al. *JAMA Network Open*

eFigure 1: Six-Minute Walk Distance at Baseline, Week 12, and Week 24

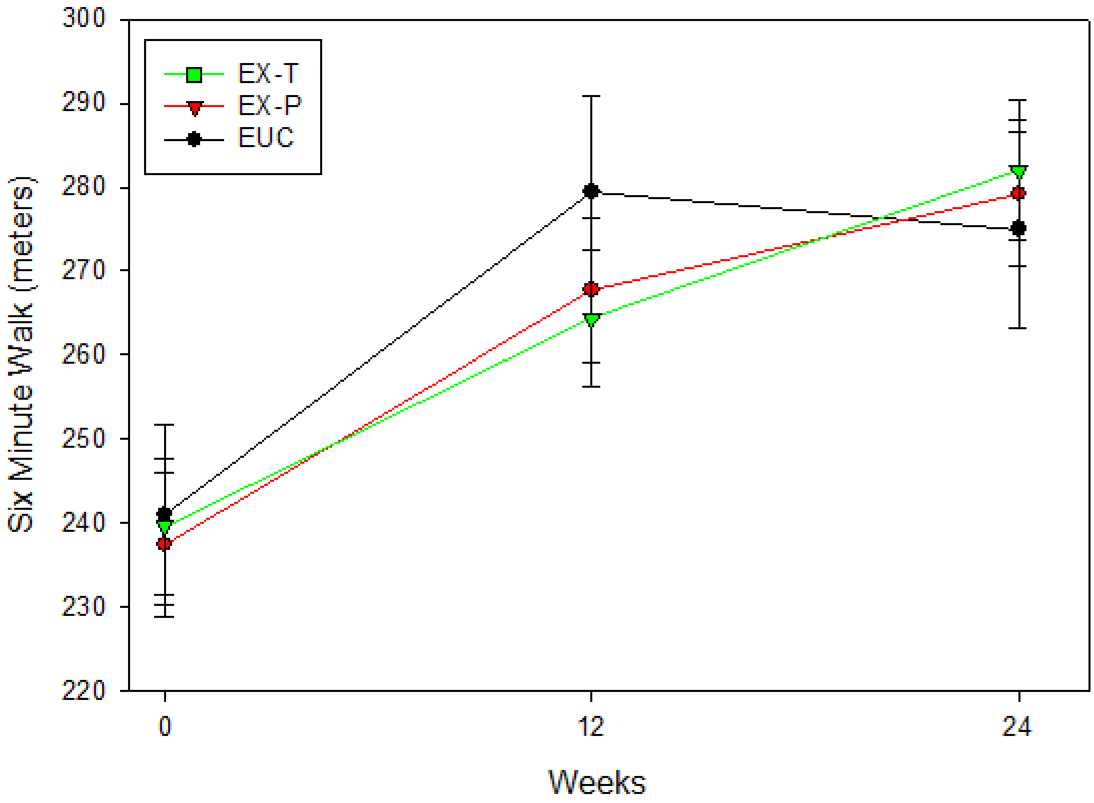

© 2025 Binder EF et al. *JAMA Network Open*

eFigure 2: Short Physical Performance Battery (SPPB) Score at Baseline, Week 12, and Week 24

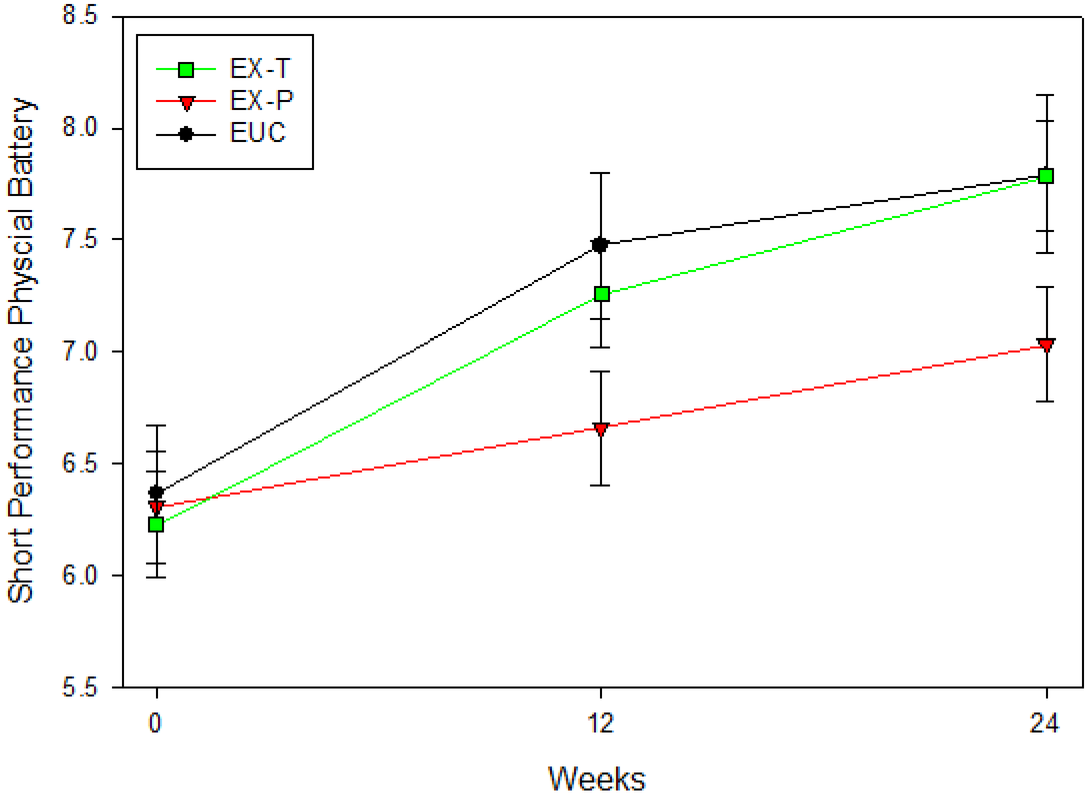

© 2025 Binder EF et al. *JAMA Network Open*

eTable 1. Participants With Adverse Events from Falls by Treatment Group

Characteristic	No. (%)		
	EX + P<br>N=54	EX + T<br>N=55	EUC<br>N=20
**Patients with falls**[a]	11 (20.4)	16 (29.1)	4 (20.0)
**No. of Falls per participant**			
1	6 (11.1)	14 (25.5)	1 (5.0)
2	3 (5.6)	2 (3.6)	3 (15.0)
≥3	2 (3.7)	0	0
**Falls by injury classification**[b]			
With injury (SAE)	1 (1.8)	2 (3.6)	0 (0.0)
With injury (AE)	6 (11.1)	10 (18.5)	1 (5.0)
Without injury	4 (7.4)	4 (7.3)	3 (15.0)

[a] P value >.05.

[b] Participants who had multiple falls of different injury severity were classified according to the most severe adverse event resulting from the fall.

© 2025 Binder EF et al. *JAMA Network Open*

eTable 2. List of Serious Adverse Events by System Organ Class and Preferred Term by Treatment Group (n=129)

There were 11 randomized participants with 20 serious adverse events.

	Treatment Group			
**System Organ Class and Preferred Term**	**EX + P**	**EX + T**	**EUC**	**Total**
**Gastrointestinal disorders**				
Constipation	0	1	0	1
Diarrhea	1	0	0	1
Diverticulum intestinal	0	1	0	1
Large intestine perforation	0	1	0	1
Vomiting bile	0	1	0	1
**General disorders**				
Chest pain	0	1	0	1
Medical device complication	1	0	0	1
**Hepatobiliary disorders**				
Cholelithiasis	0	1	0	1
**Infections and infestations**				
Pneumonia	0	0	1	1
Sepsis	0	1	0	1
Urinary tract infection	1	0	0	1
**Injury, poisoning, and procedural complications**				
Ankle fracture	1	0	0	1
Fractured sacrum	0	1	0	1
Hip fracture	0	1	0	1
Lumbar vertebral fracture	0	1	0	1
Tibia fracture	1	0	0	1
**Musculoskeletal and connective tissue disorders**				
Musculoskeletal pain	0	1	0	1
**Nervous system disorders**				
Transient ischemic attack	0	1	0	1
**Renal and urinary disorders**				
Kidney fibrosis	0	1	0	1
**Respiratory, thoracic, and mediastinal disorders**				
Acute respiratory failure	0	1	0	1
Total	5	14	1	20

© 2025 Binder EF et al. *JAMA Network Open*

eTable 3. List of Serious Adverse Events by System Organ Class and Preferred Term by Severity (N=129).

There were 11 randomized participants with 20 serious adverse events.

	Severity			
**System Organ Class and Preferred Term**	**Moderate**	**Severe**	**Fatal**	**Total**
**Gastrointestinal disorders**				
Constipation	1	0	0	1
Diarrhea	0	1	0	1
Diverticulum intestinal	0	1	0	1
Large intestine perforation	0	1	0	1
Vomiting bile	1	0	0	1
**General disorders**				
Chest pain	0	1	0	1
Medical device complication	0	1	0	1
**Hepatobiliary disorders**				
Cholelithiasis	1	0	0	1
**Infections and infestations**				
Pneumonia	0	1	0	1
Sepsis	0	0	1	1
Urinary tract infection	0	1	0	1
**Injury, poisoning, and procedural complications**				
Ankle fracture	0	1	0	1
Fractured sacrum	0	1	0	1
Hip fracture	0	1	0	1
Lumbar vertebral fracture	1	0	0	1
Tibia fracture	0	1	0	1
**Musculoskeletal and connective tissue disorders**				
Musculoskeletal pain	1	0	0	1
**Nervous system disorders**				
Transient ischemic attack	0	1	0	1
**Renal and urinary disorders**				
Kidney fibrosis	1	0	0	1
**Respiratory, thoracic, and mediastinal disorders**				
Acute respiratory failure	0	0	1	1
Total	6	12	2	20

© 2025 Binder EF et al. *JAMA Network Open*

eTable 4. Laboratory Test Results by Study Visit and Treatment Group

	Study Visit	EX + P (n=54)	EX + T (n=55)	EUC (n=20)
Alanine aminotransferase (ALT), mean (SD), U/L				
Reference range 12-49 U/L	Baseline	14.3 (5.9)	16.8 (10.4)	16.3 (5.4)
	3 Month	19.1 (27.4)	17.0 (7.9)	16.3 (4.6)
	6 Month	14.8 (5.5)	17.1 (9.9)	15.4 (5.1)
	3M - BL	4.9 (27.7)	-0.04 (8.0)	-0.27 (4.0)
	6M - BL	0.71 (4.0)	0.56 (4.4)	-0.31 (5.7)
Aspartate aminotransferase (AST), mean (SD), U/L				
Reference range 18-36 U/L	Baseline	20.8 (5.7)	22.3 (8.5)	21.2 (5.8)
	3 Month	21.7 (6.7)	21.6 (6.1)	21.9 (6.0)
	6 Month	20.2 (5.1)	21.7 (6.7)	21.6 (5.8)
	3M - BL	0.89 (5.9)	-0.42 (7.0)	0.67 (3.8)
	6M - BL	-0.21 (3.4)	0.50 (6.2)	0.19 (6.2)
Hematocrit, mean (SD), %				
Reference Range 36-45%	Baseline	38.4 (4.1)	38.2 (3.5)	39.6 (2.5)
	3 Month	39.1 (4.1)	39.3 (3.5)	39.5 (2.8)
	6 Month	39.2 (4.2)	39.1 (3.3)	39.8 (2.7)
	6M - BL	.6 (2.8)	.97 (2.1)	-0.4 (1.8)
Hemoglobin, mean (SD), g/DL				
Reference range11.5-15.5 g/DL	Baseline	12.6 (1.4)	12.6 (1.2)	13.0 (0.8)
	3 Month	12.9 (1.3)	13.0 (1.1)	13.0 (0.9)
	6 Month	13.0 (1.4)	13.1 (1.1)	13.3 (0.8)
	3M - BL	0.31 (0.7)	0.40 (0.9)	0.03 (0.9)
	6M - BL	0.31 (0.9)	0.44 (0.8)	0.11 (0.6)
HDL Cholesterol, mean (SD), mg/dL				
Optimal 50-60 mg/dL[a]	Baseline	58.2 (14.3)	66.3 (19.1)	72.0 (21.7)
	6 Month	58.2 (15.5)	65.9 (21.3)	70.9 (18.8)
	6M - BL	-0.47 (7.5)	0.45 (8.4)	-2.8 (10.2)
LDL Cholesterol, mean (SD), mg/dL				
Optimal <100 mg/dL[a]	Baseline	92.2 (26.5)	91.7 (34.0)	102.7 (32.0)
	6 Month	92.3 (27.1)	93.9 (34.1)	105.3 (32.4)
	6M - BL	1.8 (14.7)	1.8 (14.7)	0.75 (11.4)
Total Cholesterol, mean (SD), mg/dL				
Optimal <200 mg/dL[a]	Baseline	176.2 (30.7)	182.8 (41.4)	197.4 (42.8)
	6 Month	177.5 (32.3)	186.2 (46.5)	197.3 (39.8)
	6M - BL	3.0 (16.2)	2.5 (25.0)	-3.6 (15.3)
Triglycerides, mean (SD), mg/dL				
Optimal < 150 mg/dL[a]	Baseline	128.8 (67.9)	124.2 (68.0)	113.2 (43.3)
	6 Month	135.3 (72.0)	131.7 (118.9)	106.1 (32.2)
	6M - BL	8.6 (58.5)	5.5 (91.2)	-6.4 (31.3)

© 2025 Binder EF et al. *JAMA Network Open*

eTable 4. Laboratory Test Results by Study Visit and Treatment Group (Continued).

	Study Visit	EX + P (n=54)	EX + T (n=55)	EUC (n=20)
Testosterone Total [b, c]				
Reference Range for Females ≥ 19 years 8-60 ng/dL	Screening	17.2 (12.5)	15.7 (9.2)	13.0 (5.8)
	Baseline	19.4 (16.2)	15.8 (6.6)	12.8 (4.7)
	Week 2	16.1 (12.4)	229.0 (200.6)	NA
	1 Month	17.0 (13.6)	193.9 (153.4)	
	2 Month	16.2 (13.0)	164.1 (126.0)	
	3 Month	18.4 (14.5)	164.4 (194.7)	15.0 (7.3)
	4 Month	17.9 (14.5)	162.6 (136.2)	NA
	5 Month	16.4 (13.2)	169.4 (176.7)	
	6 Month	16.9 (13.1)	259.9 (1021.8)	13.8 (6.9)
Testosterone Free[c], mean (SD), ng/dL				
Reference Range for Females<br>65 < 70 years 0.06-0.84 ng/dL<br>70 < 75 years 0.06-0.82 ng/dL<br>75 < 80 years 0.06-0.79 ng/dL<br>80 < 85 years 0.06-0.76 ng/dL<br>85 < 90 years 0.06-0.73 ng/dL<br>90 < 95 years 0.06-0.71 ng/dL<br>95 – 100+ years 0.06-0.68 ng/dL	Screening	0.2 (0.1)	0.2 (0.2)	0.2 (0.1)
	Baseline	0.3 (0.1)	0.2 (0.1)	0.3 (0.1)
	Week 2	0.3 (0.2)	3.4 (3.6)	NA
	1 Month	0.2 (0.2)	2.8 (3.2)	
	2 Month	0.3 (0.2)	2.3 (2.4)	
	3 Month	0.3 (0.2)	1.9 (1.4)	0.2 (0.1)
	4 Month	0.3 (0.2)	2.2 (1.8)	NA
	5 Month	0.3 (0.2)	2.8 (4.0)	
	6 Month	0.3 (0.2)	3.2 (9.0)	0.2 (0.1)

Abbreviations: NA, not applicable.
SI conversion factors: To convert AST and ALT to µkat/L, multiply values by 0.0167.
SI conversion factors: To convert cholesterol to mmol/L, multiply values by 0.0259.
SI conversion factors: To convert hematocrit to proportion of 1.0, multiply values by 0.38.
SI conversion factors: To convert hemoglobin to g/L, multiply values by 10.
SI conversion factors: To convert triglycerides to mmol/L, multiply values by 0.0113.
SI conversion factors: To convert testosterone to nmol/L, multiply values by 0.0347.
[a] Lipid optimal ranges APTIII Guidelines for Adults greater than or equal to 18 years old, The American Heart Association.
[b] Results reported as "<7" were set at 6.9 for calculations.
[c] Per protocol, testosterone labs were measured only at screening, baseline, month 3, and month 6 in the EUC treatment group.

© 2025 Binder EF et al. *JAMA Network Open*

eTable 5. Ferriman-Gallwey Hirsutism Scores by Study Visit and Treatment Group[a]

	Study Visit	EX + P (n=54)	EX + T (n=55)	EUC (n=20)
Hormonal score (excludes legs and forearms), mean (SD)	Baseline	2.6 (2.3)	2.3 (2.4)	3.2 (2.6)
	3 Month	2.3 (2.1)	2.1 (2.0)	2.0 (1.8)
	6 Month	2.5 (2.0)	2.6 (2.5)	1.9 (2.2)
Indifferent score (forearms and legs), mean (SD)	Baseline	1.5 (1.5)	1.1 (1.4)	1.1 (1.5)
	3 Month	1.3 (1.5)	1.2 (1.4)	0.8 (1.1)
	6 Month	1.4 (1.6)	1.0 (1.2)	0.8 (0.9)

[a] F-G Hirsutism scores can range from 0 (no excessive terminal hair growth visible) to 4 (extensive hair growth visible) for each body part evaluated. The hormonal score includes nine body regions. Therefore, a total score can range from 0 to 36 and a score of ≥ 8 typically indicates hirsutism.

© 2025 Binder EF et al. *JAMA Network Open*
