# Supplementary material for: Combining Exercise Training and Testosterone Therapy in Older Women After Hip Fracture: The STEP-HI Randomized Clinical Trial
Source: JAMA Netw Open. 2025 May 15;8(5):e2510512. doi: 10.1001/jamanetworkopen.2025.10512 (PMC12082367; doi:10.1001/jamanetworkopen.2025.10512)
Supplement: Supplement 3. — Data Sharing Statement [file jamanetwopen-e2510512-s003.pdf]

Data Sharing Statement

Binder. Combining Exercise Training and Testosterone Therapy in Older Women After Hip Fracture. *JAMA Netw Open*. Published May 15, 2025.
doi:10.1001/jamanetworkopen.2025.10512

Data
Additional Information: ClinicalTrials.gov Identifier: NCT02938923
Data available: Yes
Data types: Deidentified participant data, Data dictionary
How to access data: Before a public use data set is available in anonymized file or for data not included in that file, investigators will need to submit a data request to Peter Dore at pmdore@wustl.edu
When available: With publication

Supporting Documents
Document types: None

Additional Information
Who can access the data: Data will be made available to those investigators who certify that they will not use the data for commercial purposes or purposes that go beyond the pre-specified research questions and/or hypotheses.
Types of analyses: Analyses that address research questions within the scope of the pre-specified research questions and/or hypotheses.
Mechanisms of data availability: Before a public use data set is available in anonymized file or for data not included in that file, investigators will need to submit a data request that will be reviewed for content and feasibility by the STEP HI Publications Committee. Once approved, a budget estimate will be provided by the STEP-HI Data Coordinating Center to cover costs of preparing a data set. If accepted by both user and study leader, a data use agreement (DUA) will need to be executed.
Any additional restrictions: None
